# Asymmetric Reductive Carbocyclization Using Engineered Ene Reductases

**DOI:** 10.1002/anie.201802962

**Published:** 2018-05-14

**Authors:** Kathrin Heckenbichler, Anna Schweiger, Lea Alexandra Brandner, Alexandra Binter, Marina Toplak, Peter Macheroux, Karl Gruber, Rolf Breinbauer

**Affiliations:** ^1^ Institute of Organic Chemistry Graz University of Technology Stremayrgasse 9 8010 Graz Austria; ^2^ Institute of Biochemistry Graz University of Technology Petersgasse 10–12 8010 Graz Austria; ^3^ Austrian Centre of Industrial Biotechnology (ACIB) Petersgasse 14 8010 Graz Austria; ^4^ Institute of Molecular Biosciences University of Graz Humboldtstraße 50 8010 Graz Austria

**Keywords:** asymmetric synthesis, biocatalysis, C−C-bond formation, enoate reductases, protein engineering

## Abstract

Ene reductases from the Old Yellow Enzyme (OYE) family reduce the C=C double bond in α,β‐unsaturated compounds bearing an electron‐withdrawing group, for example, a carbonyl group. This asymmetric reduction has been exploited for biocatalysis. Going beyond its canonical function, we show that members of this enzyme family can also catalyze the formation of C−C bonds. α,β‐Unsaturated aldehydes and ketones containing an additional electrophilic group undergo reductive cyclization. Mechanistically, the two‐electron‐reduced enzyme cofactor FMN delivers a hydride to generate an enolate intermediate, which reacts with the internal electrophile. Single‐site replacement of a crucial Tyr residue with a non‐protic Phe or Trp favored the cyclization over the natural reduction reaction. The new transformation enabled the enantioselective synthesis of chiral cyclopropanes in up to >99 % *ee*.

Over the last decades, the power of enzymatic catalysis has been recognized as an important tool for the stereoselective synthesis of active pharmaceutical ingredients, agrochemicals, and flavor compounds on a laboratory and industrial scale. However, biocatalysis has been largely applied for functional‐group transformations that follow the natural reactivity of the applied enzymes, such as hydrolases, reductases, oxidases, or transaminases.^1^ The use of enzymes for C−C bond formation is less well established, although notable exceptions exist. Aldolases, hydroxynitrile lyases, thiamine diphosphate dependent enzymes, and terpene cyclases have been used for this type of reactions.^2^ There is a strong desire to expand the biocatalytic toolbox with reactions that surpass the established metabolic pathways. Therefore, engineered and artificial metalloenzymes have been developed for biocatalytic C−C bond formations, including olefin cyclopropanation,^3^ Suzuki coupling,^4^ Diels–Alder reaction,^5^ and others.^6^ Herein, we report a new type of enzymatic C−C‐bond formation in which a combination of substrate design and protein engineering enabled asymmetric reductive cyclization using ene reductases. Ene reductases from the Old Yellow Enzyme (OYE) family are enzymes that reduce electron‐deficient alkenes, as present in α,β‐unsaturated carbonyl compounds. The synthetic potential of this enzyme family for biocatalytic applications has been recognized in recent years.^7^, ^8^ According to the accepted mechanism, a hydride is delivered from the reduced flavin mononucleotide (FMN) cofactor to the β‐carbon to form an enolate, which is subsequently protonated with the assistance of Tyr‐OH as a proton source.^9^ We envisioned the use (or modification) of this enzyme family for reductive carbocyclizations by offering substrates that exhibit an additional internal electrophile and thus react intramolecularly with the generated enolate intermediate to produce cyclized products.^10^, ^11^ Toward this goal, carbonyl groups, alkylhalides, or epoxides could be used as the electrophile (Scheme 1).

**Scheme 1:**
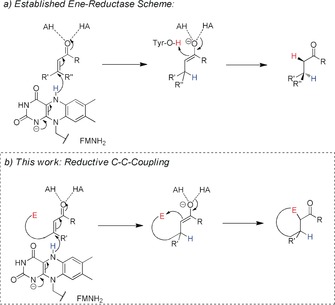
a) Reduction of an activated C=C bond by ene reductases. Activation of the double bond by hydrogen‐bond formation is enabled, for example, by two His residues (shown as “AH”/“HA”). The FMN hydride (shown in blue) is transferred to the β‐C. The resulting enolate is stabilized by two hydrogen bonds. Reprotonation at the α‐carbon occurs through a conserved tyrosine residue. b) Proposed mechanism of reductive C−C coupling. In the absence of the Tyr residue, the enolate attacks the internal electrophilic carbon, thereby enabling the formation of cyclic compounds.

Since the wild‐type (WT) enzymes OPR3 (12‐oxophytodienoic acid reductase 3) from tomato (*Solanum lycopersicum*) and YqjM from *Bacillus subtilis* have already shown their value in asymmetric reduction of alkenes in biocatalysis,^12^ we used these enzymes to explore reductive carbocyclization reactions with substrates bearing various electron‐withdrawing substituents and featuring ω‐halo alkyl groups with different chain lengths, which should give rise to different ring sizes (Scheme 2; Table 1). Interestingly, even the wild‐type enzymes displayed measurable levels of reductive cyclization when **1 a‐Br** was offered as a substrate (Table 1, entries 1 and 2). However, when (*E*)‐4‐chlorobut‐2‐enal (**1 a‐Cl**) was converted with wild‐type OPR3 and YqjM, the natural reduction pathway was dominant over the reductive cyclization reaction (Table 1, entries 3 and 4), thus indicating that with a chlorine leaving group, the γ‐carbon lacks sufficient electrophilicity. The α,β‐unsaturated ketone substrate **1 b‐Br** was also transformed into a cyclic product though in lower amounts when compared to the more reactive aldehyde substrate **1 a‐Br** (Table 1, entries 5 and 6).

**Scheme 2:**
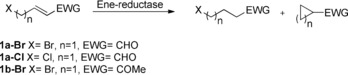
Reductive C−C bond formations using substrates **1 a‐Br**–**1 b‐Br**. Reaction conditions: 300 μL stock solution (10 mm substrate, 1 vol% DMF, 1,2‐DME as internal standard, and 15 mm NADH in 50 mm sodium phosphate‐buffer at pH 7.5 and 150 mm NaCl) and enzyme (5 μm) in 300 μL sodium phosphate‐buffer (50 mm, pH 7.5, 150 mm NaCl) per tube, 180 min and 25 °C at 300 rpm. DMF=*N,N*‐dimethylformamide, 1,2‐DME=1,2‐dimetheoxyethane.

**Table 1:** Biocatalytic conversions of substrates **1 a‐Br**–**1 b‐Br**.

Entry	Enzyme	Substrate	Conv. [%]^[a]^	Selectivity [red/c]
1	OPR3 WT	**1 a‐Br**	74	66:34
2	YqjM WT	**1 a‐Br**	>99	59:41
3	WT OPR3	**1 a‐Cl**	98	85:15
4	WT YqjM	**1 a‐Cl**	>99	83:17
5	WT OPR3	**1 b‐Br**	97	72:28
6	WT YqjM	**1 b‐Br**	>99	93:7
7	OPR3 Y190F	**1 a‐Br**	93	12:88
8	OPR3 Y190W	**1 a‐Br**	45	22:78
9	YqjM Y169F	**1 a‐Br**	>99	2:98
10	YqjM Y169W	**1 a‐Br**	3	50:50
11	OPR3 Y190F	**1 a‐Cl**	99	73:27
12	OPR3 Y190W	**1 a‐Cl**	85	82:18
13	YqjM Y169F	**1 a‐Cl**	>99	66:34
14	WT OPR3	**1 b‐Br**	97	72:28
15	OPR3 Y190F	**1 b‐Br**	97	14:86
16	OPR3 Y190W	**1 b‐Br**	85	16:84
17	WT YqjM	**1 b‐Br**	>99	93:7
18	YqjM Y169F	**1 b‐Br**	>99	2:98
19	YqjM Y169W	**1 b‐Br**	28	25:75

[a] Conversions were determined by GC‐FID analysis of the crude reaction mixture by using 1,2‐DME as an internal standard; red=reduction product; c=cyclization product; n.d.=not detected.

These initial experiments showed that alkene reduction and enolate trapping are competing pathways. Following our proposal (Scheme 1), we reasoned that the desired enolate alkylation could be favored if protonation of the enolate could be prevented or suppressed by removing proton donors and residual water molecules from the active site. We assumed that exchanging the proton donor (Tyr190 in OPR3)9c for an aprotic and apolar amino acid (Phe, Trp) should lead to beneficial effects because of an inability to directly reprotonate the intermediate as well as displacement of water from the active site. Indeed, the OPR3 variants Y190F and Y190W showed a 2‐fold improvement in the formation of cyclopropane carbaldehyde from **1 a‐Br** compared to the wild‐type enzyme (Table 1, entries 7 and 8). For engineering YqjM, the critical Tyr residue at position 169^13^ was replaced with Phe or Trp. The Phe variant of YqjM converted (*E*)‐4‐bromobut‐2‐enal (**1 a‐Br**) smoothly in more than 99 % conversion with a high preference for the C−C bond‐forming reaction over the natural reduction reaction (Table 1, entry 9). In order to demonstrate the practical value of this transformation, a (*E*)‐4‐bromobut‐2‐enal (10 mm) was transformed with YqjM Y169F (0.05 mol %) on a preparative scale. The very volatile cyclopropane carbaldehyde was further converted in a subsequent chemical reaction into the corresponding 2,4‐dinitrophenylhydrazone in 79 % yield of isolated product over two steps.

While the WT enzymes showed mainly alkene reduction of α,β‐unsaturated ketones (substrate **1 b‐Br**), YqjM Y169F turned out to be an excellent enzyme for the cyclization, producing acetylcyclopropane in 98 % conversion (Table 1, entry 18).

Ester substrates (**1 c‐Br** and **1 d‐Br**) were not converted at all by either the wild‐type ene reductase or the variants, and ring formation of 4‐ or 5‐membered rings was not observed (see Table S1 in the Supporting Information), since the less favorable kinetics of cyclization of such rings in comparison to three‐membered rings could not compete with the faster natural reduction reaction.^14^

For the α‐substituted substrate (*E*)‐4‐chloro‐2‐methylbut‐2‐enal (**4 a‐Cl**), the Phe variant of YqjM showed highest cyclopropanation activity (up to 76 %; Table S2). In order to test for the possibility of performing asymmetric reductive carbocyclizations, (*E*)‐4‐chloro‐3‐methylbut‐2‐enal (**5‐Cl**) and (*E*)‐4‐bromo‐3‐methylbut‐2‐enal (**5‐Br**) were used as substrates for wild‐type OPR3 and YqjM and the variants thereof (Scheme 3).

**Scheme 3:**
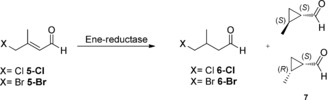
Biocatalytic conversion of substrates **5‐Cl** and **5‐Br**. Reaction conditions as for Scheme 2.

All of the enzymes converted both substrates equally well in up to >99 % conversion, with the OPR3 and YqjM variants showing a higher preference for cyclopropanation activity (Table 2). With the more electrophilic substrate **5‐Br**, these variants delivered almost exclusively the desired cyclopropanation product (Table 2, entries 7–8, 10). Interestingly, WT YqjM and its Phe variant showed a reversal in diastereoselectivity when switching from a chlorine to the bromine substrate (Table 2, entries 4,5 vs. 9,10).

**Table 2:** Biocatalytic conversion of substrates **5‐Cl** and **5‐Br**.

Entry	Enzyme	X	Conv. [%]^[a]^	Selectivity [red/c]	*de trans/cis* [%]^[b]^	*ee* [%]^[b]^ (*S*,*S*)‐**7**	*ee* [%]^[b]^ (*R*,*S*)‐**7**
1	WT OPR3	Cl	>99	57:43	68	87	>99
2	OPR3 Y190F	Cl	>99	19:81	57	61	54
3	OPR3 Y190W	Cl	>99	22:78	64	31	24
4	WT YqjM	Cl	95	69:31	67	52	56
5	YqjM Y169F	Cl	>99	12:88	72	−80	−55
6	WT OPR3	Br	>99	13:87	14	92	n.d.
7	OPR3 Y190F	Br	>99	1:99	58	22	n.d.
8	OPR3 Y190W	Br	70	1:99	13	39	n.d.
9	WT YqjM	Br	>99	35:65	−54	81	n.d.
10	YqjM Y169F	Br	>99	1:99	−30	−67	n.d.

[a] Conversions were determined by GC‐FID analysis of the crude reaction mixture by using 1,2‐DME as an internal standard; n.d.=not detected. [b] For assignment of *de* and *ee* products were analyzed as the corresponding alcohols after reduction of the samples using NaBH_4_.

Very good enantioselectivity was observed for WT OPR3, which had a strong preference for the (*R*,*S*)‐**7** enantiomer (>99 % *ee*; Table 2, entry 1). The YqjM Y169F variant showed reversed as well as enhanced enantioselectivity compared to the wild‐type enzyme, thus indicating that subtle changes in the binding site might have large consequences for substrate recognition (Table 2, entry 5). We were able to rationalize the observed asymmetric induction through docking studies (Figure S1 in the Supporting Information).

We reasoned that the enantiodiscrimination in the ene reductase enabled carbocyclization could be enhanced by offering a substrate bearing a sterically demanding phenyl substituent at C‐β to the wild‐type and variant ene reductases (Scheme 4). WT OPR3 converted substrate **8‐Cl** into cyclopropane **10** with moderate conversion (48 %), whereas OPR3 Y190F and OPR3 Y190W led to good conversion to **10** (75 % and 71 %; Table 3, entries 1 to 3). Replacement of Tyr169 with Phe in YqjM is key to achieving a high carbocyclization activity and excellent diastereoselectivity that strongly favors the *trans* diastereomer (*R*,*R*‐**10**, *de* 94 %; Table 3, entries 4–5). Both OPR3 and YqjM (WT and variants) showed a high preference for the (1*R*,2*R*)‐2‐phenylcyclopropyl)methanol (*R*,*R*‐**10**), giving >99 % *ee*.

**Scheme 4:**
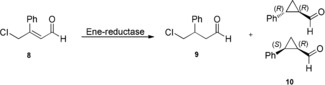
Biocatalytic conversion of substrate **8**. Reaction conditions as for Scheme 2.

**Table 3:** Biocatalytic conversion of substrate **8**.

Entry	Enzyme	Conv. [%]^[a]^	Selectivity [red/c]	*de trans/cis* [%]	*ee* [%]^[b]^ (*R*,*R*)‐**10**	*ee* [%]^[b]^ (*S*,*R*)‐**10**
1	WT OPR3	>99	52:48	72	>99	54
2	OPR3 Y190F	>99	25:75	71	>99	−43
3	OPR3 Y190W	94	29:71	71	>99	−51
4	WT YqjM	81	28:72	−30	>99	−8
5	YqjM Y169F	89	5:95	94	>99	−29

[a] Conversions were determined by GC‐FID analysis of the crude reaction mixture by using 1,2‐DME as an internal standard. [b] For assignment of *ee*, products were analyzed as the corresponding alcohols after reduction of the samples using NaBH_4_.

In conclusion, we have presented the first biocatalytic reductive carbocyclization using ene reductases. For our cyclization approach, two strategies proved useful to suppress the natural reduction reaction. First, engineering of the enzymes by replacing the critical proton donor (Tyr190 in OPR3, Tyr169 in YqjM) with Phe or Trp favored the preference for the cyclization reaction. Second, substrate engineering could be used in a synergistic way.

While an increase in the electrophilicity of the γ‐carbon favored the cyclization, this approach was often limited by the intrinsic instability of these substrates. Decoration of the scaffold with substituents at the α‐ and β‐position (as present in synthetically more attractive compounds) appeared to be a more universal strategy, leading to improved product selectivity by removing water molecules that could serve as potential alternative proton donors in the active site. We were able to demonstrate that prochiral substrates can be cyclized to form chiral 1,2‐disubstituted cyclopropanes with excellent enantioselectivity. Current efforts in our laboratory aim to expand the scope to larger ring systems by using protein and substrate engineering.

## Conflict of interest

The authors declare no conflict of interest.

## Supporting information

As a service to our authors and readers, this journal provides supporting information supplied by the authors. Such materials are peer reviewed and may be re‐organized for online delivery, but are not copy‐edited or typeset. Technical support issues arising from supporting information (other than missing files) should be addressed to the authors.

Supplementary
