# Supplementary material for: Asymmetric Reductive Carbocyclization Using Engineered Ene Reductases
Source: Angew Chem Int Ed Engl. 2018 May 14;57(24):7240–4. doi: 10.1002/anie.201802962 (PMC6033016; doi:10.1002/anie.201802962)
Supplement: Supplementary file 1 — Supplementary [file ANIE-57-7240-s001.pdf]

Angewandte Chemie
Eine Zeitschrift der Gesellschaft Deutscher Chemiker

Supporting Information

Asymmetric Reductive Carbocyclization Using Engineered Ene Reductases

*Kathrin Heckenbichler, Anna Schweiger, Lea Alexandra Brandner, Alexandra Binter, Marina Toplak, Peter Macheroux, Karl Gruber, and Rolf Breinbauer**

anie_201802962_sm_miscellaneous_information.pdf

Supporting Information

1 Tables.......1

2 Quantum chemical calculations.......2

3 Docking.......2

4 Experimental Part.......4

4.1 General aspects.......4

4.2 Dry Solvents.......4

4.3 Analytical Methods.......5

4.3.1 Achiral GC-FID.......5

4.3.2 Chiral GC-FID.......5

4.3.3 Achiral GC-MS.......6

4.3.4 Reversed phase HPLC-MS.......6

4.3.5 Chiral normal phase HPLC.......6

4.3.6 Nuclear Magnetic Resonance Spectroscopy.......7

4.3.7 High resolution mass spectrometry.......7

4.3.8 Determination ofmelting points.......7

4.3.9 Specific Optical Rotation.......7

4.3.10 Thin layer chromatography.......7

4.4 Flash column chromatography.......8

4.5 Hydrogenation.......8

4.6 General considerations when synthesizing α,β-unsaturated aldehydes and performing reactions with α,β-unsaturated aldehydes.......9

4.7 Experimental Procedures.......10

4.7.1 Synthesis of α,β-unsaturated esters and ketones.......10

4.7.1.1 (2-Ethoxy-2-oxoethyl)triphenylphosphoniumbromide (**13**).......10

4.7.1.2 Ethyl 2-(triphenyl-λ^5-phosphanylidene)acetate (**14**).......11

4.7.1.3 Ethyl (*E*)-4-hydroxybut-2-enoate (**15**).......12

4.7.1.4 Ethyl (*E*)-4-chlorobut-2-enoate (**16**).......12

4.7.1.5 Ethyl (*E*)-5-hydroxypent-2-enoate (**17**) 14
4.7.1.6 Ethyl (*E*)-5-bromopent-2-enoate (**18**) 14
4.7.1.7 Ethyl (*E*)-5-iodopent-2-enoate (**19**) 16
4.7.1.8 1-(Triphenyl-λ^5-phosphanylidene)propan-2-one (**20**) 17
4.7.1.9 (*E*)-5-Bromopent-3-en-2-one (**21**) 18
4.7.2 Synthesis of α,β-unsaturated α-methyl substituted esters 19
4.7.2.1 (1-Ethoxy-1-oxopropan-2-yl)triphenylphosphonium bromide (**22**) 19
4.7.2.2 Ethyl 2-(triphenyl-λ^5-phosphanylidene)propanoate (**23**) 20
4.7.2.3 Ethyl (*E*)-4-hydroxy-2-methylbut-2-enoate (**24**) 21
4.7.2.4 Ethyl (*E*)-4-chloro-2-methylbut-2-enoate (**25**) 21
4.7.2.5 Ethyl (*E*)-4-bromo-2-methylbut-2-enoate (**26**) 22
4.7.3 Synthesis of α,β-unsaturated β-methyl substituted esters 24
4.7.3.1 Ethyl (*E*)-4-hydroxy-3-methylbut-2-enoate (**27**) 24
4.7.3.2 Ethyl (*E*)-4-chloro-3-methylbut-2-enoate (**28**) 24
4.7.3.3 Ethyl (*E*)-4-bromo-3-methylbut-2-enoate (**29**) 25
4.7.4 Synthesis of α,β-unsaturated β-aryl substituted esters 27
4.7.4.1 1-Phenyl-2-((tetrahydro-2*H*-pyran-2-yl)oxy)ethan-1-one (**30**) 27
4.7.4.2 Ethyl (*E*)-3-phenyl-4-((tetrahydro-2*H*-pyran-2-yl)oxy)but-2-enoate (**31**).. 28
4.7.4.3 Ethyl (*E*)-4-hydroxy-3-phenylbut-2-enoate (**32**) 29
4.7.4.4 Ethyl (*E*)-4-chloro-3-phenylbut-2-enoate (**33**) 30
4.7.5 Synthesis of saturated aldehydes and ketones 31
4.7.5.1 4-Bromobutanal (**34**) 31
4.7.5.2 4-Chlorobutanal (**35**) 32
4.7.5.3 5-Bromopentan-2-one (**36**) 32
4.7.5.4 Ethyl 4-hydroxy-3-methylbutanoate (**37**) 33
4.7.5.5 Ethyl 4-chloro-3-methylbutanoate (**38**) 34
4.7.5.6 Ethyl 4-hydroxy-2-methylbutanoate (**39**) 35
4.7.5.7 Ethyl 4-chloro-2-methylbutanoate (**40**) 35

4.7.5.8 Ethyl 4-bromo-3-methylbutanoate (**41**) 36

4.7.5.9 Ethyl 4-hydroxy-3-phenylbutanoate (**42**).... 36

4.7.5.10 Ethyl 4-chloro-3-phenylbutanoate (**43**).... 37

4.7.5.11 General Procedure for the reduction of saturated esters (**38**, **40**, **41**, **43**) to the corresponding aldehydes (**6-Cl**, **6-Br**, **46**, **9**) 39

4.7.5.12 4-Bromo-3-methylbutanal (**6-Br**) 39

4.7.5.13 4-Chloro-3-methylbutanal (**6-Cl**).... 40

4.7.5.14 4-Chloro-2-methylbutanal (**46**).... 40

4.7.5.15 4-Chloro-3-phenylbutanal (**9**) 41

4.7.6 Synthesis of racemic and chiral cyclopropanes.... 43

4.7.6.1 1-Methylcyclopropane-1-carbaldehyde (**48**).... 43

4.7.6.2 *trans*-(2-Phenylcyclopropyl)methanol (*rac*)(***trans*-49**) 43

4.7.6.3 *trans*-2-Phenylcyclopropane-1-carbaldehyde (*rac*) (***trans*-10**).... 44

4.7.6.4 (*Z*)-2-Phenylethen-1-ol (**51**).... 45

4.7.6.5 *cis*-(2-Phenylcyclopropyl)methanol (*rac*) (***cis*-49**) 46

4.7.6.6 *cis*-2-Phenylcyclopropane-1-carbaldehyde (*rac*) (***cis*-10**) 46

4.7.6.7 (*Z*)-But-2-en-1-ol (***cis*-52**) 47

4.7.6.8 (*E*)-But-2-en-1-ol (***trans*-52**).... 48

4.7.6.9 *cis*-(2-Methylcyclopropyl)methanol (*rac*) (***cis*-53**).... 48

4.7.6.10 *trans*-(2-Methylcyclopropyl)methanol (*rac*) (***trans*-53**) 49

4.7.6.11 ((1*S*,2*R*)-2-Methylcyclopropyl)methanol(**(*S*,*R*)-53**) 50

4.7.6.12 ((1*S*,2*S*)-2-Methylcyclopropyl)methanol (**(*S*,*S*)-53**) 52

4.7.6.13 (4*S*,5*S*)-2-Butyl-N^4,N^4,N^5,N^5-tetramethyl-1,3,2-dioxaborolane-4,5-dicarboxamide (**(4*S*,5*S*)-54**) 54

4.7.6.14 (4*R*,5*R*)-2-Butyl-N^4,N^4,N^5,N^5-tetramethyl-1,3,2-dioxaborolane-4,5-dicarboxamide (**(4*R*,5*R*)-54**) 54

4.7.7 Synthesis of aldehydes for reductive cyclization reactions.... 55

4.7.7.1 General procedure for reduction with DIBAL-H for unsaturated esters **1d-Br**, **16**, **25**, **28**, **29**, **33** 55

4.7.7.2 General oxidation procedure with $MnO_2$to substrates **1a-Br**, **1a-Cl**, **2a-Br**, **2a-I**, **3a-Br**, **3a-I** 56

4.7.7.3 (*E*)-4-Bromobut-2-enal (**1a-Br**) 57

4.7.7.4 (*E*)-4-Chlorobut-2-enal (**1a-Cl**) 57

4.7.7.5 (*E*)-4-Chloro-3-methylbut-2-enal (**5**) 58

4.7.7.6 (*E*)-4-Bromo-3-methylbut-2-enal (**7**) 58

4.7.7.7 (*E*)-4-Chloro-3-phenylbut-2-enal (**10**) 59

4.7.7.8 (*E*)-4-Chloro-2-methylbut-2-enal (**4a-Cl**) 60

4.7.7.9 Ethyl (*E*)-5-bromopent-2-enoate (**2a-Br**) 60

4.7.7.10 Ethyl (*E*)-5-iodopent-2-enoate (**2a-I**) 61

4.7.7.11 (*E/Z*)-6-Bromohex-2-enal (***E*/Z 3a-Br**) 62

4.7.7.12 (*E*)-6-Bromohex-2-enal (**3a-Br**) 62

4.7.7.13 5-Iodo-1-pentene (**55**) 63

4.7.7.14 (*E/Z*)-6-Iodohex-2-enal (***E*/Z 3a-I**) 64

4.7.7.15 (*E*)-6-Iodohex-2-enal (**3a-I**) 64

4.8 Biocatalysis Section 66

4.9 Analytical scale procedure for enzymatic reductive cyclization reactions 66

4.10 Determination of diastereomericexcess, enantiomeric excess and assignment of absolute configuration 66

4.10.1 Diastereomericexcess and enantiomeric excess of (2-methylcyclopropyl)methanol 67

4.10.2 Diastereomericexcess and enantiomeric excess of (2-phenylcyclopropyl)methanol 69

4.11 Preparative scale procedure for enzymatic reductive cyclization reactions 75

4.11.1 1-(Cyclopropylmethylene)-2-(2,4-dinitrophenyl)hydrazine (**55**) 75

4.12 Generation of enzyme variants of OPR3 and YqjM 76

4.12.1 DNA sequences and translated amino acid sequenesof ene-reductase variants. 77

4.12.2 Preparation of buffers 83

4.12.3 Expression of OPR3 and YqjM wild types and variants 83

4.12.4 Purification of OPR3 wild type and variants 84

4.12.5 Purification of YqjM wild type and variants 84

4.12.6 Estimation of enzyme concentrations based on absorption spectra 85

5 References 89

6 NMR-Appendix 91

1 Tables

Table S1: Conversions of substrates **2a-I**, **2a-Br**, **3a-I**, **3a-Br**, **1d-Br** and **1c-Br**.

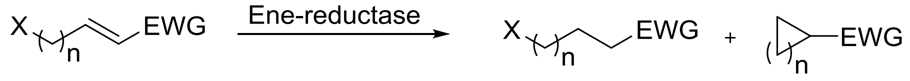

1c-Br X= Br, n=1, EWG= CO_2Me
1d-Br X= Br, n=1, EWG= CO_2Et
2a-Br X= Br, n=2, EWG= CHO
2a-I X= I, n=2, EWG= CHO
3a-Br X= Br, n=3, EWG= CHO
3a-I X= I, n=3, EWG= CHO

Entry	Enzyme	Substrate	Conv.[%][a]	Selectivity [red:c]
20	OPR3 Y190F	**2a-I**	>99	100: 0
21	OPR3 Y190F	**2a-Br**	>99	100: 0
22	OPR3 Y190F	**3a-I**	26	100: 0
23	OPR3 Y190F	**3a-Br**	34	100: 0
24	all enzymes	**1d-Br**	<1	n.d.
25	all enzymes	**1c-Br**	<1	n.d.

[a] Conversions were determined by GC-FID analysis of the crude reaction mixture by using 1,2-DME as an internal standard; red = reduction product; c = cyclization product; n.d.= not detected.

Table S2: Conversion of substrate **4a-Cl**.

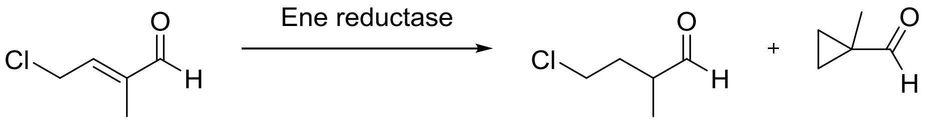

4a-Cl

Entry	Enzyme	Conversion [%][a]	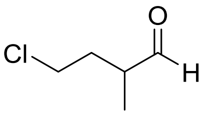	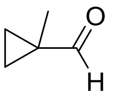
1	OPR3 WT	>99	54	46
2	OPR3 Y190F	>99	32	68
3	OPR3 Y190W	>99	35	65
4	YqjM WT	>99	44	56
5	YqjM Y169F	>99	24	76
6	YqjM Y169W	nd	nd	nd

[a]Conversions were determined by GC-FID analysis of the crude reaction mixture by using 1,2-DME as an internal standard; nd= not detected.

2 Quantum chemical calculations

Transition states for the reductive cyclization of **5-Cl** were calculated using the program Jaguar employing DFT calculations with the B3LYP functional and the 6-31g*+ basis set.[1] Suitable starting structures for the transition state searches were generated using relaxed coordinate scans starting from optimized structures of the respective enolate intermediates resulting from a hydride transfer to the C-β-atom. The nature of the thus identified stationary points were checked by frequency calculations. In all cases a single imaginary frequency was found which corresponded to the supposed reaction coordinate (formation of the C-C bond and cleavage of the C-Cl bond). Transition state structures were calculated for formation of both the *cis*- and the *trans*-cyclopropane derivatives.

3 Docking

Coordinates for OPR3 and YqjM were obtained from the PDB (entry-codes: 2hsa and 1z41) and were prepared for the docking calculations by the addition of polar hydrogen atoms, the addition of partial charges as well as by assigning the appropriate atom types. The structures of the active site variants (OPR3-Y190F and YqjM-Y169F) were generated with the program PyMOL (http://www.pymol.org). Structures of the substrate **5-Cl** were built using the program Maestro (Schrödinger Inc.).

Docking calculations were performed with the program AutoDockVina using the default parameters except for an "exhaustiveness" of 32.[2] The cubic energy grid was centered on the N5 atom of the FMN cofactor and had an extension of 22.5 Å in each direction. Whereas all ligand structures were treated as flexible to ensure a maximum degree of freedom during the calculations, all protein residues were kept rigid. The resulting docking poses were analyzed using the program PyMOL (http://www.pymol.org).

In an attempt to rationalize the enantiodiscrimination observed in the asymmetric cyclization reaction substrate **5-Cl** was docked into the active sites of OPR3 and YqjM using the program AutoDock Vina. In both cases, structures of the wild type enzymes as well as of the Tyr to Phe variants were used. Figure 18 shows the modeled complex of OPR3 WT with **5-Cl**. The binding mode of the compound is typical for OYE substrates with the carbonyl group forming hydrogen bonds to His185 and His188. The C-β-atom is located close to N5 of the flavin

cofactor (ideally placed to receive a hydride). The methyl group is oriented toward a small hydrophobic pocket, whereas the chloro-methyl group is exposed to the solvent.

We also calculated ring closure transition state structures starting from the corresponding enolate compound derived from **5-Cl** using the program Jaguar[17] and again docked these structures into the active sites of OPR3 and YqjM. Figure 1 shows the transition state structure for the formation of the *(R,S)*-**7** bound to OPR3 wild type, which exhibits an ee of >99% in this conversion (Table 2). The modeled binding mode is very similar to the binding mode of **5-Cl** and only small conformational changes are necessary to reach the transition state after hydride transfer to C-β. The chloride leaving group may interact with the side chains of His244 and/or Tyr370.

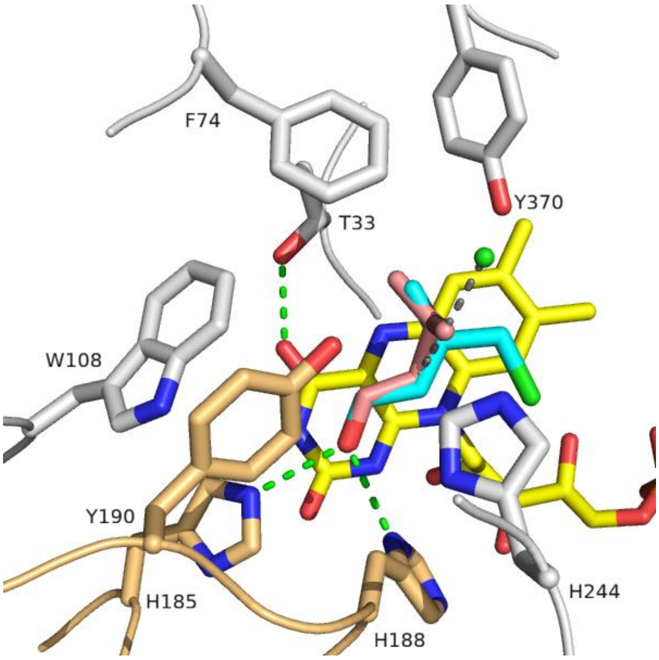

Figure S1: Close-up view of the active site of OPR3 WT in complex with the substrate **5-Cl** (cyan) and the transition state leading to *(R,S)*-**7** (pink). Hydrogen bonding interactions are shown as green dashed lines, the bonds broken and formed in the transition state are shown as grey lines. The figure was prepared using the program PyMOL (https://www.pymol.org/).

4 Experimental Part

4.1 General aspects

Commercially available reagents and solvents were purchased from Sigma Aldrich, Alfa Aesar, Roth, Lactan, abcr, VWR, Fisher Scientific or Acros Organics and were used without further purification unless otherwise mentioned. All experiments were carried out using standard Schlenk techniques under an inert atmosphere of argon or nitrogen. When applying Schlenk techniques the glass apparatus was dried under oil pump vacuum by heating with a heat gun, cooled to RT, and flushed with inert gas. Dry solvents were prepared by the below-mentioned procedures and stored under an inert atmosphere. The stated temperatures generally refer to the oil bath or the cooling bath temperature. The water bath temperature of the rotary evaporator was usually set to 35 °C unless otherwise noted.

4.2 Dry Solvents

Dry solvents were stored over3 Å or 4 Å molecular sieves (Sigma Aldrich, beds, 8-12 mesh). The molecular sieves were activated by filling a 500 mL round bottom flask to one third of its volume and heating the flask in a heating mantle at level 1 under oil pump vacuum for 2 d until complete dryness was obtained.

Dry **dichloromethane** was prepared in two steps. First EtOH-stabilized dichloromethane was heated under reflux for 1 d over phosphorous pentoxide and distilled, then heated under reflux for 2 d over calcium hydride, and then distilled under argon atmosphere into a dry, brown 1 L Schlenk bottle with activated 4 Å molecular sieves.

Dry **dimethylsulfoxide** was purchased from Acros Organics and stored over activated 4 Å molecular sieves in a dry, brown 1 L Schlenk bottle under an argon atmosphere.

Dry **toluene** was purchased from Sigma Aldrich and was dried using an aluminum oxide column (Pure Solv® by Innovative Technology) and was stored over activated 4 Å molecular sieves in a dry, brown 1 L Schlenk bottle under an argon atmosphere.

Triethylaminewas dried over calcium hydride and then distilled under argon atmosphere into a dry, brown 1 L Schlenk bottle with activated 4 Å molecular sieves.

Pyridine was purchased in anhydrous quality and stored over molecular sieves in a dark bottle under argon.

Non dry **diethylether** was purchased from Roth and the stabilizer was removed by distillation using a rotary evaporator. The solvent was stored in a brown glass bottle over KOH.

Dry **THF** was heated under reflux over sodium until benzophenone indicated the dryness of the solvent. It was stored in a brown 1 L Schlenk bottle with activated 4 Å molecular sieves. Non dry **THF** was distilled to remove the stabilizer using a rotary evaporator. It was stored over KOH in a brown glass-bottle.

Non dry solvents, i.e. cyclohexane, ethylacetate, methanol, dichloromethane, toluene, acetone, ethanol were purchased from VWR, Fisher Scientific, or Sigma Aldrich and used as obtained.

4.3 Analytical Methods

4.3.1 Achiral GC-FID

Achiral GC-FID measurements were performed on an Agilent Technologies 6890N GC system with an Agilent Technologies J&W GC-column DB-17-01 ((14%-cyanopropylphenyl) methylpolysiloxane; 30 m x 250 µm x 0.25 µm). For injection, an Agilent Technologies 7683 Series auto sampler (split mode) was used. Nitrogen 5.0 served as carrier gas and hydrogen 5.0 and air were used for detection together with a flame ionization detector (FID).

Temperature program:

KH_80_30_280: 1 min at 80 °C, ramp 30 °C min^{-1} linear to 280 °C.

4.3.2 Chiral GC-FID

Chiral GC-FID measurements were performed on a Hewlett Packard 6890 GC System with an Agilent CP-Chirasil-Dex CB column (25 m×320 µm×0.25m). For injection, a Hewlett Packard 7683 Series autosampler (split mode) was used. Nitrogen 5.0 served as carrier gas and hydrogen 5.0 and air were used for detection together with a flame ionization detector (FID). Injector temperature was set to 200 °C and detector temperature was set to 250 °C, split ratio was set to 80:1.

Temperature program:

KH_40_60_155: Start at 40 °C, ramp 2 °C min^{-1} linear to 60 °C, 10 min at 60 °C, ramp 20 °C min^{-1} linear to 155 °C.

4.3.3 Achiral GC-MS

GC-MS measurements were performed on an Agilent Technologies 7890A (G3440A) GC system equipped with anAgilent Technologies J&W GC-column HP-5MS ((5%-phenyl) methylpolysiloxane; length: 30m; inner-diameter: 0.250 mm; film: 0.25 µm) at a constant helium flow rate with He 5.0 as carrier gas. The sample was injected in split mode using an Agilent Technologies 7683 Seriesautosampler and an Agilent Technologies 7683B Series injector. The GC was coupled to a 5975C inert mass sensitive detector with triple-axis detector (MSD, EI, 70 eV; transfer line:300°C; MS source: 240°C; MS quad: 180°C).

Temperature programm:

MT_50_S: 50°C 1 min, ramp: 40°C·min$_{-1}$ linear to 300°C, 300°C 5 min, solventdelay: 4.0 min.

4.3.4 Reversed phase HPLC-MS

Analytical HPLC-MS measurements were performed on an Agilent 1200 Series MWD SL UV detector. Signals were detected at λ = 210 nm. The separation of the analytes was carried out using a "C-18 reversed-phase" column of the type „Poroshell® 120 EC-C18, 3.0 x 100 mm, 2.7 µm". The following methods were used for performing the separations:

method_1: 0.0 – 6.0 min 98% water/0.1% HCOOH and 2% CH_3CN linear decrease to 100% CH_3CN, 6.0 – 8.0 min 100% CH_3CN; 0.7 mL/min, 30 °C.

4.3.5 Chiral normal phase HPLC

Chiral HPLC measurements were performed on an Agilent 1100 Series HPLC system equipped with a temperature controlled column oven. Detection of the substances was accomplished with a Diode Array Detector at a wavelength of λ = 210 nm. The separations were carried out either on a Daicel Chiralcel OD-H column (0.46 cm×25 cm) or a Daicel Chiralcel OJ-H column (0.46 cm×25 cm). The following methods were used for the separation:

90_n-hexane_ODH: *n*-hexane: iPrOH 9:1, flow= 0.5 mL min^{-1}.

95_n-hexane_OJH:*n*-hexane: iPrOH 95:5, flow= 0.5 mL min^{-1}.

4.3.6 Nuclear Magnetic Resonance Spectroscopy

The described nuclear magnetic resonance spectra were recorded on a BrukerAvanceIII spectrometer (300.36 MHz for ^{1}H-NMR, 75.53 MHz for ^{13}C-NMR) with autosampler or a Varian Unity Inova NB high resolution spectrometer (499.90 MHz for ^{1}H-NMR, 125.71MHz for ^{13}C-NMR). The residual solvent peak was set as internal standard. Coupling constants (J) are reported as absolute values in Hertz (Hz) and chemical shifts are reported in parts per million (ppm). To confirm and identify a structure additional NMR experiments (NOESY, H,H-COSY, HSQC, APT, HMBC) were measured.

4.3.7 High resolution mass spectrometry

The measurement of high resolution mass spectrometry spectra was performed on a “Waters GCT Premier” system after ionisation with an EI ionisation source of a potential of 70 V. The corresponding calculated and measured masses are noted for each compound.

4.3.8 Determination ofmelting points

Melting points were determinated using a Mel-Temp® melting point apparatus with integrated microscopical support from Electrothermal in open capillary tubes. Melting points were not corrected.

4.3.9 Specific Optical Rotation

The specific optical rotation was determined on a Perkin Elmer Polarimeter 341 with an integrated sodium vapor lamp. All samples were measured at the D-line of the sodium light (λ = 589 nm) under tempered conditions 20 °C. Concentrations between 5.0 $g.L^{-1}$ (c = 0.50) and 20.0 $g.L^{-1}$ (c = 2.00) depending on the solubility of the sample were chosen, CH_2Cl_2 and $CHCl_3$ were used as solvents.

4.3.10 Thin layer chromatography

Reactions were monitored by thin layer chromatography on commercial silica gel plates (TLC, aluminium foil, Merck, 60 F_{254}). The detection occurred by using an UV lamp (254 or 360 nm) or a stain reagent was applied and the plates were developed using a stream of hot air.

$KMnO_4$: 0.3 g $KMnO_4$ and 20 g K_2CO_3 were dissolved in 300 mL H_2O. Under stirring 5 mL 5 % aqueous NaOH were added.

CAM: 50 g Ammonium molybdate, 2.0 g $Ce(SO_4)_2$ and 50 mL concentrated sulfuric acid were dissolved in 400 mL distilled water.

Developing solvents and R_f-values are stated for each compound.

4.4 Flash column chromatography

For preparative flash column chromatography silica gel from Acros Organics (silica gel, forchromatography 0.035 – 0.070 mm, 60 Å, nitrogen flushed) was used. The amount of silica depends on the specific separation problem and was in general the 20 to 100 fold amount of crude product. The column diameter was chosen to give a filling level between 15 and 25 cm.As fraction size, typically one third of the volume of the used silica gel volume waschosen. The eluent was selected to result in aR_f-value between 0.2 and 0.3 of the to-be-isolated substance. If the crude product was not soluble in the eluent, the sample was dissolved in a proper solvent (DCM or EtOAc) and the 1.5 fold amount of silica gel was added, followed by removing the solvent using a rotary evaporator and drying invacuum.

4.5 Hydrogenation

High pressure hydrogenation experiments were performed, utilizing an H-Cube^{TM} continuoushydrogenation unit (HC-2.SS) from Thales Nanotechnology Inc. with a KnauerSmartline pump 100, equipped with a 10 mL ceramic pump head. As hydrogenation catalyst a 10 % palladiumon carbon powder cartridge (Thales Nanotechnology Inc., THS 01111, 10 % Pd/C CatCart^{TM}), or a Raney-Nickel cartridge (Thales Nanotechnology Inc., THS 01112, Raney-Nickel CatCart^{TM}) was used.

The workup of batch hydrogenation experiments utilizing metal catalysts was performed by filtering off the catalyst using a pad of SiO_2 under inert conditions and elution with MeOH or EtOAc. The hydrogenation catalyst was washed with H_2O under inert gas and then stored in a glass bottle covered with water.

4.6 General considerations when synthesizing α,β-unsaturated aldehydes and performing reactions with α,β-unsaturated aldehydes

The reactions described in 4.7.7.1, 4.7.7.2, 4.8 and 4.11 had to be performed under the exclusion of light. The light in the laboratory and the fume hood was switched off. The glass windows of the doors were covered with aluminum-foil and doors were closed during experiments. The windows were covered by blinds and the glass of the closed fume hood was covered by paper card boards. Working in the fume hood was achieved by opening just one window. Round bottom flask were covered with aluminum-foil or black garbage bags. When evaporation was needed a big paper card box was used to cover the water bath of the evaporator. Column chromatography was performed in the completely covered fume hood. α,β-unsaturated aldehydes were prepared freshly when biocatlytic experiments were performed. NMR experiments had to be done immediately when α,β- unsaturated aldehydes were isolated. It was not possible to store the α,β-unsaturated aldehydes.

4.7 Experimental Procedures

Methyl (*E*)-4-bromobut-2-enoate (**1c-Br**), methyl 4-bromobutanoate, ethyl (*E*)-4-bromobut-2-enoate (**1d-Br**), ethyl 4-bromobutanoate, ethylcyclopropanecarboxylate, methylcyclopropanecarboxylate, 1-cyclopropylethan-1-one, cyclopropanecarbaldehyde, cyclobutanecarbaldehyde and cyclopentanecarbaldehyde were purchased from Sigma Aldrich.

4.7.1 Synthesis of α,β-unsaturated esters and ketones

4.7.1.1 (2-Ethoxy-2-oxoethyl)triphenylphosphoniumbromide (13)

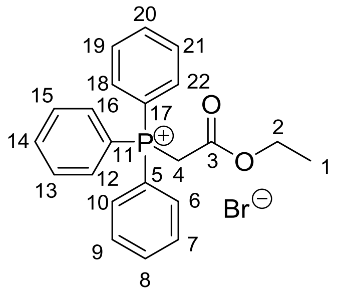

13

In a flame dried 250 mL two-neck round-bottom flask equipped with nitrogen inlettriphenylphosphine (42.15 g, 0.16 mol) was dissolved in 100 mL dry toluene. Ethylbromoacetate (16.2 mL, 0.14 mol) was added dropwise via a syringe in a N_2 counter-stream and a colourless precipitate was formed immediately. The suspension was stirred at 22 °C overnight. The colourless solid was washed with toluene (3×100 mL) and cyclohexane (3×100 mL) and was dried under oil pump vacuum.

yield: 62.25 g (99 %), white powder, $C_{22}H_{22}BrO_2P$, [429.29 g/mol].

m.p.= 153 °C.

^{1}H-NMR (300.36 MHz, DMSO-d$_6$): δ= 7.84 (ddd, $^3J_{HP}$=19.1 Hz, $^4J_{HP}$=12.0 Hz, $^5J_{HP}$=5.5 Hz, 15H, H-6, H-7, H-8, H-9, H-10, H-12, H-13, H-14, H-15, H-16, H-18, H-19, H-20, H-21, H-22), 5.35 (d, $^2J_{HP}$= 14.5 Hz, 2H, H-4), 4.04 (q, $^3J_{HH}$= 7.1 Hz, 2 H, H-2), 0.97(t, $^3J_{HH}$= 7.1 Hz, 3H, H-1) ppm.

^{13}C-NMR (75.53 MHz, $CDCl_3$): δ= 164.6 (d,$^2J_{CP}$= 3.8 Hz, C-3), 135.1 (d, $^4J_{CP}$=3.0 Hz, C-8, C-14, C-20), 133.7 (d, $^3J_{CP}$= 10.7 Hz, C-7, C-9, C-13, C-15, C-19, C-21), 130.1 (d, $^2J_{CP}$= 13.0 Hz, C-6, C-10, C-12, C-16, C-18, C-22), 118.2 (d, $^1J_{CP}$= 88.9 Hz, C-5, C-11, C-17), 62.3 (C-2), 29.6 (d,$^1J_{CP}$= 56.0 Hz, C-4), 13.5 (C-1) ppm.

The recorded spectra are in accordancewith the reported in literature.[3]

4.7.1.2 Ethyl 2-(triphenyl-λ⁵-phosphanylidene)acetate (14)

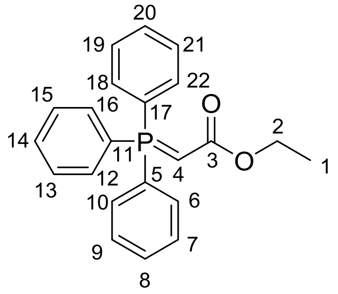

14

In a 1000 mL round bottom flask (2-ethoxy-2-oxoethyl)triphenylphosphonium bromide (**14**) (58.42 g, 136 mmol) was dissolved in 350 mL distilled H_2O and 400 mL toluene. Under stirring 10 % aqueous NaOH solution (50 mL) was added until a pH of 10 was achieved. The layers were separated and the aqueous phase was washed with dichloromethane (3×150 mL). The combined organic layers were dried over Na_2SO_4 and the solvent was removed under reduced pressure. After addition of 140 mL cyclohexane to the yellow oil, colourless crystals precipitated. The crystals were collected by filtration and washed with cold cyclohexane (3×150 mL). The white crystals were dried under oil pump vacuum.

yield: 27.95 g (59 %), colourless, crystalline solid, $C_{22}H_{21}O_2P$, [348.13 g/mol].

m.p.= 126-130 °C.

^{1}H-NMR (300.36 MHz, DMSO-d$_6$): δ= 7.64-7.57 (m, 15 H, H-6, H-7, H-8, H-9, H-10, H-12, H-13, H-14, H-15, H-16, H-18, H-19, H-20, H-21, H-22), 3.83 (q, $^3J_{HH}$= 6.9 Hz, 2H, H-2), 2.76 (d, $^2J_{HP}$= 22.7 Hz, 1H, H-4), 1.10(t, $^3J_{HH}$= 7.0 Hz, 3H, H-1) ppm.

^{13}C-NMR (75.53 MHz, $CDCl_3$): δ= 170.4 (d,$^2J_{CP}$= 14.6 Hz, C-3), 132.5 (d, $^4J_{CP}$=10.0 Hz, C-8, C-14, C-20), 132.1 (d, $^3J_{CP}$= 9.1 Hz, C-7, C-9, C-13, C-15, C-19, C-21), 128.9 (d, $^2J_{CP}$= 12.0 Hz, C-6, C-10, C-12, C-16, C-18, C-22), 127.4 (d, $^1J_{CP}$=91.2 Hz, C-5, C-11, C-17), 56.6 (C-2), 28.8 (d,$^1J_{CP}$=127.9 Hz, C-4), 14.7 (C-1) ppm.

The recorded spectra are in accordancewith the reported in literature.[3]

4.7.1.3 Ethyl (*E*)-4-hydroxybut-2-enoate (15)

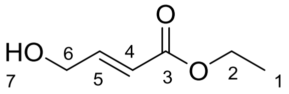

15

In a 250 mL round-bottom flask glycolaldehyde dimer(1.98 g,16.5 mmol) was dissolved in CH_2Cl_2. Ethyl 2-(triphenyl-λ^5-phosphanylidene)acetate (**14**)(11.48 g, 33.0 mmol) wasadded and the yellow reaction mixture was stirred under reflux at 45 °C for 4 h. After full conversion was indicated by TLC the solvent was removed under reduced pressure and the product was purified via flash chromatography (500 g silica gel, 20×7.5 cm, cyclohexane/ethylacetate 2/1, fraction size: 250 mL).

yield:2.62 g (61 %), yellow oil, $C_6H_{10}O_3$, [130.14 g/mol]

R_f= 0.38 (cyclohexane/ethylacetate 1/1) ($KMnO_4$)

GC-FID (KH_80_30_280): t_R= 4.6 min

^{1}H-NMR (300.36 MHz, $CDCl_3$): δ= 7.00 (q, $^3J_{HH}$= 3.9 Hz, 1H, H-5), 6.07 (d, $^3J_{HH}$= 15.7 Hz, 1H, H-4), 4.31 (d, $^3J_{HH}$= 1.4 Hz, 2H, H-6), 4.17 (q, $^3J_{HH}$= 7.1 Hz, 2H, H-2), 1.27 (t, $^3J_{HH}$= 7.1 Hz, 3H, H-1) ppm.

^{13}C-NMR (75.53 MHz, $CDCl_3$): δ =166.3 (C-3), 146.81 (C-4), 119.7 (C-5), 61.4 (C-6), 60.2 (C-2), 13.9 (C-1) ppm.

The recorded spectra are in accordancewith the reported in literature.3

4.7.1.4 Ethyl (*E*)-4-chlorobut-2-enoate (16)

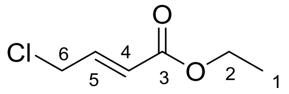

16

In a flame dried 250 mL two-neck round-bottom flask with nitrogen inlet and a bubble counter ethyl (*E*)-4-hydroxybut-2-enoate (**15**) (5.42 g, 41.6 mmol) was dissolved in 115 mL dry CH_2Cl_2 and cooled to -20 °C using an acetone/dry ice bath. Dry pyridine (23.5 mL, 23.0 g, 291 mmol) and PPh_3 (43.70 g, 166.6 mmol) were added and the clear, colorless solution was stirred for 20 min at -20 °C. NCS (11.12 g, 83.2 mmol) was added in small portions within 60 min and the reaction solution turned dark brown. After slowly warming the reaction mixture to 22 °C (2 h), TLC indicated complete consumption of starting material.

The reaction mixture was quenched by the addition of 1M HCl (100 mL). Addition of CH_2Cl_2 (50 mL) and centrifugation at 5000 rpm for 15 min produced two phases which were separated. The aqueous phase was extracted with CH_2Cl_2 (3x50 mL) and the combined organic layers were washed with 10 w% aq. $CuSO_4$ solution (3x100 mL) and brine (1x 100 mL). The organic phase was dried over Na_2SO_4 and the solvent was removed under reduced pressure. The product was purified via flash chromatography (250 g silica gel, 12×7.5 cm, cyclohexane/ethylacetate 20/1, fraction size: 125 mL).

yield:3.15 g (51 %), slightly yellowliquid, $C_6H_9ClO_2$, [148.59 g/mol]

R_f= 0.58(cyclohexane/ethylacetate 1/1) ($KMnO_4$)

^{1}H-NMR (300.36 MHz, $CDCl_3$): δ= 6.97 (q, $^3J_{HH}$= 6.1 Hz, 1H, H-5), 6.09 (d, $^3J_{HH}$= 15.4 Hz, 1H, H-4), 4.22 (d, $^3J_{HH}$= 7.1 Hz, 2H, H-6), 4.15 (q, $^3J_{HH}$= 4.1 Hz, 2H, H-2), 1.29 (t, $^3J_{HH}$= 7.1 Hz, 3H, H-1) ppm.

^{13}C-NMR (75.53 MHz, $CDCl_3$): δ= 165.6 (C-3), 141.6 (C-4), 124.1 (C-5), 60.7 (C-6), 42.5 (C-2), 14.2 (C-1) ppm.

The recorded spectra are in accordancewith the reported in literature.[4]

4.7.1.5 Ethyl (*E*)-5-hydroxypent-2-enoate (17)

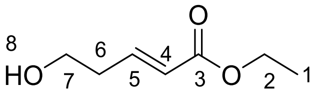

17

In a 100 mL round-bottom flask propane-1,3-diol (0.14 mL, 1.9 mmol) was dissolved in 50 mL CH_2Cl_2. Ethyl 2-(triphenyl-λ^5-phosphanylidene)acetate (**14**)(1.59 g, 4.6 mmol) and activated MnO_2 (3.3 g, 38 mmol) were added. The black reaction mixture was stirred at 24 °C for 12 h. After full conversion was indicated by TLC, MnO_2 was removed via filtration through a pad of silica and rinsed with CH_2Cl_2 (3×50 mL). The solvent was evaporated under reduced pressure. Flash chromatography (38 g silica gel (50-fold excess), cyclohexane/EtOAc = 2/1, fraction size: 25 mL) gave the desired product.

yield: 0.21 g (1.4 mmol, 75 %), orange oil, $C_7H_{12}O_3$ [144.17 g/mol]

R_f = 0.76 (EtOAc) ($KMnO_4$)

^{1}H NMR (300.36 MHz; $CDCl_3$) δ = 6.95 (dt, $^3J_{HH}$ = 15.5, 7.1 Hz, 1H, H-5), 5.91 (d, $^3J_{HH}$ = 15.7 Hz, 1H, H-4), 4.17 (q, $^3J_{HH}$ = 7.1 Hz, 2H, H-2), 3,76 (t, $^3J_{HH}$= 6.3 Hz, 2H, H-7), 2.46 (td, $^3J_{HH}$ = 7.3, 1.1 Hz, 2H, H-6), 1.27 (t, $^3J_{HH}$ = 7.1 Hz, 3H, H-1) ppm.

^{13}C NMR (75.53 MHz; $CDCl_3$) δ = 166.5 (C-3), 145.2 (C-5), 123.9 (C-4), 61.2 (C-2), 60.5 (C-7), 35.6 (C-6), 14.4 (C-1) ppm.

The recorded spectra are in accordancewith the reported in literature.[5]

4.7.1.6 Ethyl (*E*)-5-bromopent-2-enoate (18)

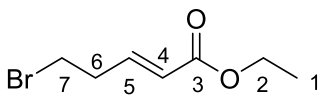

18

In a 250 mL round-bottom flask ethyl (*E*)-5-hydroxypent-2-enoate (**17**)(1.12 g, 7.76 mmol) was dissolved in 15 mL CH_2Cl_2. After addition of CBr_4 (3.89 g, 11.7 mmol) to the light orange solution, the reaction mixture was cooled to 0 °C using an ice/water bath. A solution of PPh_3 (2.70 g, 10.3 mmol, 1.3 eq) in 15 mL CH_2Cl_2 was added to the reaction mixture via dropping funnel within 10 min to give a colorless solution. As TLC indicated full conversion

of the starting material after 15 min, the solvent was removed under reduced pressure. The product was purified via flash chromatography (425 g silica gel (50-fold excess), cyclohexane/EtOAc = 30/1, fraction size: 300 mL).

yield: 1.42 g (6.86 mmol, 88 %), light yellow oil, $C_7H_{12}O_2Br$ [207.06 g/mol]

R_f = 0.86 (cyclohexane/EtOAc = 1/1) ($KMnO_4$)

GC-FID: t_R = 4.98 min

^{1}H NMR (300.36 MHz; $CDCl_3$) δ = 6.89 (dt, $^3J_{HH}$ = 15.6, 6.9 Hz, 1H, H-5), 5.91 (d, $^3J_{HH}$ = 15.7 Hz, 1H, H-4), 4.20 (q, $^3J_{HH}$ = 7.1 Hz, 2H, H-2), 3.45 (t, $^3J_{HH}$= 6.8 Hz, 2H, H-7), 2.77 (tt, $^3J_{HH}$ = 6.8, 3.4 Hz, 2H, H-6), 1.29 (t, $^3J_{HH}$ = 7.1 Hz, 3H, H-1) ppm.

^{13}C NMR (75.53 MHz; $CDCl_3$) δ = 166.2 (C-3), 144.6 (C-5), 124.0 (C-4), 60.6 (C-2), 35.2 (C-6), 30.0 (C-7), 14.4 (C-1) ppm.

The recorded spectra are in accordancewith the reported in literature.[6]

4.7.1.7 Ethyl (*E*)-5-iodopent-2-enoate (19)

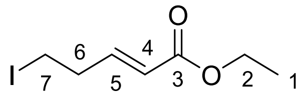

19

In a 250 mL two-neck round-bottom flask ethyl (*E*)-5-bromopent-2-enoate (**18**) (1.98 g, 9.6 mmol) was dissolved in 60 mL distilled acetone. After addition of NaI (3.60 g, 24 mmol) the colorless solution turned yellow and a colorless precipitate was formed. The reaction mixture was stirred at 60 °C for 7 h until full conversion was indicated by GC-FID. After cooling to 22 °C the precipitate was dissolved in 150 mL of distilled H_2O. The reaction solution was extracted with *n*-pentane (3x150 mL). The combined organic layers were washed with brine and dried over Na_2SO_4. The solvent was evaporated under reduced pressure. The product was used in the next step without further purification.

yield: 2.22 g (8.7 mmol, 91%), light yellow oil, $C_7H_{11}IO_2I$ [254.063 g/mol]

GC-FID: t_R = 5.45 min

^{1}H NMR (300.36 MHz; $CDCl_3$) δ = 6.84 (dt, $^3J_{HH}$ = 15.5, 6.8 Hz, 1H, H-5), 5.89 (d, $^3J_{HH}$ = 15.7 Hz, 1H, H-4), 4.20 (q, $^3J_{HH}$ = 7.1 Hz, 2H, H-2), 3.21 (t, $^3J_{HH}$= 7.1 Hz, 2H, H-7), 2.79 (q, $^3J_{HH}$ = 7.0 Hz, 2H, H-6), 1.29 (t, $^3J_{HH}$ = 7.1 Hz, 3H, H-1) ppm.

^{13}C NMR (75.53 MHz; $CDCl_3$) δ = 166.3 (C-3), 146.2 (C-5), 123.5 (C-4), 60.6 (C-2), 36.0 (C-6), 14.4 (C-1), 1.6 (C-7) ppm.

The recorded spectra are in accordancewith the reported in literature.[7]

4.7.1.8 1-(Triphenyl-λ^5-phosphanylidene)propan-2-one (20)

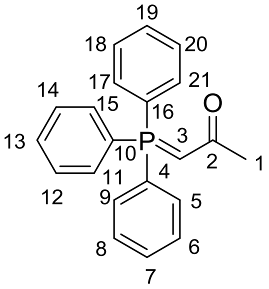

20

In a flame dried 250 mL three-neck round-bottom flask with nitrogen inlet triphenylphosphine (19.7 g, 75.2 mmol) and 1-chloropropan-2-one (5.0 mL, 5.8 g, 62.7 mmol) were dissolved in 50 mL dry chloroform. The mixture was heated to 65 °C under a nitrogen atmosphere for 10 h. A colourless precipitate was formed, which was washed with ice-cold Et_2O (3×50 mL). The solid was dissolved in a mixture of250 mL distilled H_2O/CH_2Cl_2(1/1) and under stirring 10 % aqueousNaOHsolution was added (60 mL). The phases were separated and the aqueous phase was extracted with dichloromethane (1×50 mL). The combined organic phases were washed with brine (2×100 mL), dried over Na_2SO_4, filtrated, and concentrated under reduced pressure. The colourless crystals were dried under oil pump vacuum for 5 h.

yield: 8.47 g (44 %), colourless, crystalline solid, $C_{21}H_{19}OP$, [318.26 g/mol].

m.p.= 202-205 °C

^{1}H-NMR (300.36 MHz, $CDCl_3$): δ= 7.90-7.29 (m, 15H, H-5, H-6, H-7, H-8, H-9. H-11, H-12, H-13, H-14, H-15, H-17, H-18, H-19, H-20, H-21), 3.63 (d, $^3J_{HH}$= 26.8 Hz, 1H, H-3), 2.02 (d, $^3J_{HH}$= 1.4 Hz, 3H, H-1) ppm.

^{13}C-NMR (75.53 MHz, $CDCl_3$): δ= 190.7 (d, $^2J_{CP}$= 1.7 Hz, C-2), 133.0 (d, $^3J_{CP}$= 2.7 Hz, C-6, C-8, C-12, C-14, C-18, C-20), 131.8 (d, $^4J_{CP}$= 2.7 Hz,C-7, C-13, C-19), 128.7 (d, $^2J_{CP}$=12.1 Hz, C-5, C-9, C-11, C-15, C-17, C-21), 127.2 (d, $^1J_{CP}$= 90.7 Hz, C-4, C-10, C-16), 51.4 (d, $^1J_{CP}$= 107.5 Hz, C-3), 28.4 (d, $^3J_{CP}$= 15.6 Hz, C-1) ppm.

The recorded spectra are in accordancewith the reported in literature.[8]

4.7.1.9 (*E*)-5-Bromopent-3-en-2-one (21)

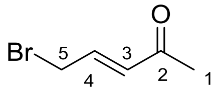

21

A 250 mL one-neck round-bottom flask was charged with 2-bromo-1,1-diethoxyethane (3.0 mL, 3.81 g, 19.3 mmol) and TFA (30.0 mL, 44.1 g, 387 mmol).The slightly yellow solution was stirred at 22 °C until GC-FID indicated complete consumption of starting material (4 h). The reaction mixture was diluted by the addition of distilled H_2O (40 mL), the phases were separated, and the aqueous phase was extracted with CH_2Cl_2 (3x40 mL). The combined organic layers were washed with brine (2x40 mL), dried over Na_2SO_4, and the solution was concentrated at a pressure of 600 mbar (with care to not exceed 35 °C bath temperature) until a final volume of 100 mL.Then 1-(triphenyl-λ^5-phosphanylidene)propan-2-one (**20**) (7.37 g, 23.2 mmol) was added and the reaction solution turned bright yellow. After stirring the reaction mixture for 2 h at 22 °C full conversion of the starting material was observed by GC-FID and the solvent was evaporated under reduced pressure. The crude product was purified via flash chromatography (500 g silica gel, 20×7.5 cm, cyclohexane/ethylacetate 6/1, fraction size: 250 mL).

yield: 406 mg (11 %), slightly yellow liquid, C_5H_7BrO, [163.01 g/mol]

R_f= 0.32 (cyclohexane/ethylacetate 4/1) ($KMnO_4$)

GC-FID (KH_80_30_280): t_R= 3.9 min

^{1}H-NMR (300.36 MHz, $CDCl_3$): δ= 6.82 (dt, $^3J_{HH}$= 15.7, 6.1 Hz, 1H, H-4), 6.23 (d, $^3J_{HH}$= 15.7 Hz, 1H, H-3), 4.03 (d, $^3J_{HH}$= 7.3 Hz, 2H, H-5), 2.29 (s, 3H, H-1) ppm.

^{13}C-NMR (75.53 MHz, $CDCl_3$): δ= 197.8 (C-2), 140.5 (C-4), 133.1 (C-3), 29.6 (C-5), 27.6 (C-1) ppm.

HRMS (EI): Calcd. (m/z) for C_5H_6O [M-HBr]: 82.0419; found: 82.0420.

4.7.2 Synthesis of α,β-unsaturated α-methyl substituted esters

4.7.2.1 (1-Ethoxy-1-oxopropan-2-yl)triphenylphosphonium bromide (22)

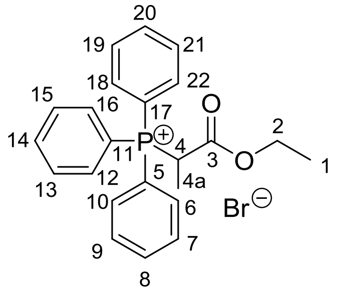

22

In a flame dried 250 mL three-neck round-bottom flask with nitrogen inlet triphenylphosphine (50.0 g, 190 mmol) was dissolved in ethyl 2-bromopropanoate (37 mL, 286 mmol). The mixture was heated to 51 °C under a nitrogen atmosphere for 10 h. Then cyclohexane (250 mL) was added to the solid and the solid cake was broken up with a spatula into smaller pieces. The colourless solid was washed with cyclohexane (3×150 mL) and dried under oil pump vacuum.

yield: 80.49 g (64 %), colourless powder, $C_{23}H_{24}BrO_2P$, [443.32 g/mol].

m.p.= 129-131 °C.

^{1}H-NMR (300.36 MHz, $CDCl_3$): δ= 7.93 (dd, $^3J_{HP}$=12.6 Hz, $^3J_{HH}$=7.4 Hz, 6H, H-6, H-10, H-12, H-16, H-18, H-22), 7.79-7.56 (m, 9H, H-7, H-8, H-9, H-13, H-14, H-15, H-19, H-20, H-21), 6.78 (dq, $^2J_{HP}$= 14.3 Hz, $^3J_{HH}$= 7.1 Hz, 1H, H-4), 3.95 (m, 2H, H-2),1.63 (dd, $^3J_{HP}$= 18.5 Hz,$^3J_{HH}$= 7.1 Hz, 3H, H-4a),0.94(t, $^3J_{HH}$= 7.1 Hz, 3H, H-1) ppm.

^{13}C-NMR(75.53 MHz, $CDCl_3$): δ= 168.0 (C-3), 134.9 (d, $^4J_{CP}$=3.0 Hz, C-8, C-14, C-20), 134.3 (d, $^3J_{CP}$= 10.0 Hz, C-7, C-9, C-13, C-15, C-19, C-21), 130.2 (d, $^2J_{CP}$= 12.8 Hz, C-6, C-10, C-12, C-16, C-18, C-22), 117.9 (d, $^1J_{CP}$= 86.3 Hz, C-5, C-11, C-17), 62.9 (C-2), 36.7 (d, $^1J_{CP}$= 50.3 Hz, C-4), 29.6 (d,$^1J_{CP}$= 56.0 Hz, C-4), 13.6 (C-1), 13.0 (d, $^2J_{CP}$= 2.8 Hz, C-4a) ppm.

The recorded spectra are in accordancewith the reported in literature.[9]

4.7.2.2 Ethyl 2-(triphenyl-λ^5-phosphanylidene)propanoate (23)

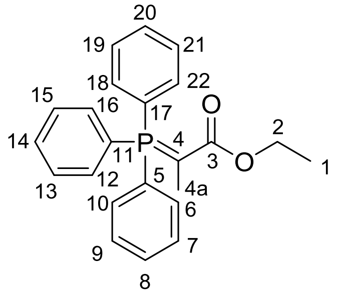

23

In a 500 ml round bottom flask NaOH (14.9 g, 373 mmol) was dissolved in 150 mL distilled H_2O. The solution was cooled to 0 °C using an ice/water bath and a solution of (1-ethoxy-1-oxopropan-2-yl)triphenylphosphonium bromide (**22**) (80.49 g, 182 mmol) in 75 mL dichloromethane was added slowly. The biphasic yellow solution was warmed to 22 °C over 2 h. The phases were separated and the aqueous phase was extracted with CH_2Cl_2(1×50 mL). The combined organic layerswere washed with brine (2×100 mL), dried over Na_2SO_4, and concentrated under reduced pressure. The yellow crystals were dried under oil pump vacuum for 5 h.

yield: 60.35 g (92 %), yellow, crystalline solid, $C_{23}H_{23}O_2P$, [362.41 g/mol].

m.p.=155-157 °C

^{1}H-NMR (300.36 MHz, $CDCl_3$): δ= 7.65-7.36 (m, 15H, H-6, H-7, H-8, H-9, H-10. H-12, H-13, H-14, H-15, H-16, H-18, H-19, H-20, H-21, H-22), 4.03 (q, $^3J_{HH}$= 6.9 Hz, 2H, H-2_{minor}), 3.69 (q, $^3J_{HH}$= 7.0 Hz, 2H, H-2_{major}), 1.59 (dd, $^3J_{HP}$= 14.0 Hz,$^3J_{HH}$= 5.0 Hz, 3H, H-4a),1.22(t, $^3J_{HH}$= 6.9 Hz, 3H, H-1_{minor}),0.43(t, $^3J_{HH}$= 7.1 Hz, 3H, H-1_{major}) ppm.

^{13}C-NMR (75.53 MHz, $CDCl_3$): δ= 133.7 (d, $^4J_{CP}$= 9.5 Hz, C-8, C-14, C-20), 132.2 (d, $^3J_{CP}$= 9.9 Hz, C-7, C-9, C-13, C-15, C-19, C-21), 131.6 (C-6, C-10, C-12, C-16, C-18, C-22), 117.9 (d, $^1J_{CP}$=12.0 Hz, C-5, C-11, C-17), 57.4 (C-2), 36.4(C-4), 14.2 (C-1), 13.0 (d, $^2J_{CP}$= 13.0 Hz, C-4a) ppm.

The recorded spectra are in accordancewith the reported in literature.9

4.7.2.3 Ethyl (*E*)-4-hydroxy-2-methylbut-2-enoate (24)

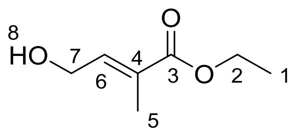

24

In a 250 mL one-neck round-bottom flask 1,4-dioxane-2,5-diol (1.98 mg, 33.0 mmol) was dissolved in 170 mL CH_2Cl_2. Ethyl 2-(triphenyl-λ^5-phosphanylidene)propanoate (**23**) (11.96 g, 33.0 mmol) was added in one portion and the reaction mixture was heated to 45 °C until TLC indicated full conversion of the starting material after 2 h. Then the solvent was removed under reduced pressure and the product was purified via flash chromatography (400 g silica gel, 18 × 7.5 cm, cyclohexane/ethylacetate 3/1, fraction size: 250 mL).

yield:4.06 g (85 %), slightly yellowliquid, $C_7H_{12}O_3$, [144.17 g/mol]

R_f= 0.64 (cyclohexane/ethylacetate 1/1) ($KMnO_4$)

GC-FID (KH_80_30_280): t_R= 4.9 min

^{1}H-NMR (300.36 MHz, $CDCl_3$): δ= 6.82 (dd,$^3J_{HH}$= 6.0, 4.9 Hz, 1H, H-4), 4.34 (d, $^3J_{HH}$= 5.7 Hz, 2H, H-7), 4.19 (q, $^3J_{HH}$= 7.1 Hz, 2H, H-2), 1.90 (dd, $^3J_{HH}$= 12.9, 8.2 Hz, 1H, H-5), 1.83 (s,3H, H-6), 1.29 (t, $^3J_{HH}$= 7.1 Hz, 3H, H-1) ppm.

^{13}C-NMR (75.53 MHz, $CDCl_3$): δ= 167.4 (C-3), 139.6 (C-4), 128.4 (C-5), 60.5 (C-2), 59.4 (C-7), 13.9 (C-1), 12.4 (C-6) ppm.

The recorded spectra are in accordance with the reported in literature.[10]

4.7.2.4 Ethyl (*E*)-4-chloro-2-methylbut-2-enoate (25)

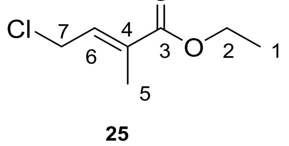

25

In a flame dried 250 mL two-neck round-bottom flask with nitrogen inlet and a bubble counterethyl (*E*)-4-hydroxy-2-methylbut-2-enoate (**24**) (1.56 g, 10.8 mmol) was dissolved in 36 mL dry CH_2Cl_2 and cooled to -20 °C using an acetone/dry ice bath. Dry pyridine(6.10 mL, 5.98 g, 75.7 mmol) and PPh_3 (11.35 g, 43.3 mmol) were added and the clear, colorless

solution was stirred for 20 min at -20 °C. NCS (2.89 g, 21.6 mmol) was added in small portions within 30 min and the reaction solution turned dark brown. After slowly warming the reaction mixture to 22 °C (2 h), TLC indicated complete consumption of starting material. The reaction mixture was quenched by the addition of 1M HCl (50 mL). Addition of CH_2Cl_2 (50 mL) and centrifugation at 5000 rpm for 15 min produced two phases which were separated. The aqueous phase was extracted with CH_2Cl_2 (3x50 mL) and the combined organic layers were washed with 10 w% aq. $CuSO_4$ solution (3x60 mL) and brine (1x 100 mL). The organic phase was dried over $Na_2SO_4$and the solvent was removed under reduced pressure. The product was purified via flash chromatography (250 g silica gel, 12×7.5 cm, cyclohexane/ethylacetate 20/1, fraction size: 125 mL).

yield:1.04 g (59 %), slightly yellowliquid, $C_7H_{11}ClO_2$, [162.61 g/mol]

R_f= 0.28 (cyclohexane/ethylacetate 20/1) ($KMnO_4$)

^{1}H-NMR (300.36 MHz, $CDCl_3$): δ= 6.82 (t, $^3J_{HH}$= 7.2 Hz, 1H, H-6), 4.25-4.15 (m, 4H, H-2, H-7), 1.92 (s, 3H, H-5), 1.30 (t, $^3J_{HH}$= 7.1 Hz, 3H, H-1) ppm.

^{13}C-NMR (75.53 MHz, $CDCl_3$): δ= 166.8 (C-3), 134.4 (C-6), 131.5 (C-4), 60.6 (C-2), 38.8 (C-7), 13.8 (C-1), 12.0 (C-5) ppm.

Configuration of the double bond was confirmed via the observation of an NOE effect between the protons at C-5 and the protons at C-7.

HRMS (EI): Calcd. (m/z) for $C_7H_{10}O_2$ [M-HCl]: 126.0681; found: 126.0682.

4.7.2.5 Ethyl (*E*)-4-bromo-2-methylbut-2-enoate (26)

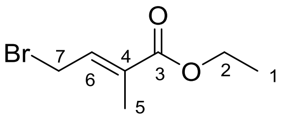

26

A 250 mL one-neck round-bottom flask was charged with ethyl (*E*)-4-hydroxy-2-methylbut-2-enoate (**24**) (4.80 g, 33.3 mmol) dissolved in 35 mL CH_2Cl_2 and was cooled using an ice/water bath. CBr_4 (16.6 g,50.0 mmol) was added and a solution of PPh_3 (5.76 g, 40.0 mmol) in 40 mL CH_2Cl_2 was added dropwise to the reaction mixture at 0 °C. After 30 min stirring at 22 °C TLC indicated full conversion of starting material and the reaction mixture was concentrated to dryness using a rotary evaporator. The product was purified via

flash chromatography (375 g silica gel, 15×8 cm, *n*-pentane/Et_2O 50/1, fraction size: 100 mL).

yield: 3.61 g (52 %), colourless liquid, $C_7H_{11}BrO_2$, [207.07 g/mol]

R_f= 0.35 (cyclohexane/ethylacetate 50/1) ($KMnO_4$)

^{1}H-NMR (300.36 MHz, $CDCl_3$): δ= 6.92 (td, $^3J_{HH}$= 8.5, 1.5 Hz, 1H, H-6), 4.20 (q, $^3J_{HH}$= 7.1 Hz, 2H, H-2), 4.03 (d, $^3J_{HH}$= 8.5 Hz, 2H, H-7), 1.92 (s, 3H, H-5), 1.30 (t, $^3J_{HH}$= 7.1 Hz, 3H, H-1) ppm.

^{13}C-NMR (75.53 MHz, $CDCl_3$): δ= 167.4 (C-3), 134.9 (C-6), 132.4 (C-4), 61.2 (C-2), 26.2 (C-7), 14.3 (C-1), 12.3 (C-5) ppm.

The recorded spectra are in accordance with the reported in literature.[11]

Configuration of the double bond was confirmed via the observation of an NOE effect between the protons at C-5 and the protons at C-7.

4.7.3 Synthesis of α,β-unsaturated β-methyl substituted esters

4.7.3.1 Ethyl (*E*)-4-hydroxy-3-methylbut-2-enoate (27)

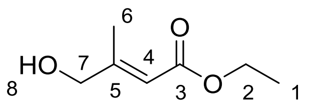

27

In a 250 mL one-neck round-bottom flask hydroxyacetone (3.0 mL, 3.18 g, 42.9 mmol) was dissolved in 100 mL MeCN. Ethyl 2-(triphenyl-λ^5-phosphanylidene)acetate (**14**) (18.3 g,2.5 mmoL, 1.2 eq) was added in one portion and the reaction mixture was heated to 70 °C until TLC indicated full conversion of starting material after 3 h. Then the solvent was removed under reduced pressureand the product was purified via flash chromatography (225 g silica gel, 15×6.5 cm, cyclohexane/ethylacetate 8/1, fraction size: 100 mL).

yield: 3.45 g (55 %), slightly yellowliquid, $C_7H_{12}O_3$, [144.17 g/mol]

R_f= 0.23 (cyclohexane/ethylacetate 8/1) ($KMnO_4$)

^{1}H-NMR (300.36 MHz, DMSO-d_6): δ= 5.88 (s, 1H, H-8), 5.20 (t, $^3J_{HH}$= 5.6 Hz, 1H, H-4), 4.07 (q, $^3J_{HH}$= 7.1 Hz, 2H, H-2), 3.94 (d, $^3J_{HH}$= 5.0 Hz, 2H, H-7), 1.98 (s,3H, H-6), 1.20 (t, $^3J_{HH}$= 7.1 Hz, 3H, H-1) ppm.

^{13}C-NMR (75.53 MHz, DMSO-d_6): δ= 166.0 (C-3), 159.6 (C-5), 112.0 (C-4), 65.2 (C-7), 59.0 (C-2), 15.3 (C-6), 14.2 (C-1) ppm.

The recorded spectra are in accordance with the reported in literature.[12]

4.7.3.2 Ethyl (*E*)-4-chloro-3-methylbut-2-enoate (28)

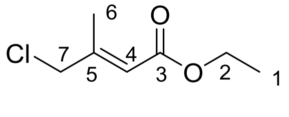

28

In a 100 mL one-neck round-bottom flask 1-chloropropan-2-one (0.8 mL, 925 mg, 9.94 mmol) was dissolved in 20 mL MeCN. Ethyl 2-(triphenyl-λ^5-phosphanylidene)acetate (**14**) (3.8 g, 10.9 mmoL) was added in one portion and the reaction mixture was heated to 70 °C until TLC indicated full conversion of starting material after 24 h. Then the solvent was

removed under reduced pressureand the product was purified via flash chromatography (125 g silica gel, 12.5×5.0 cm, cyclohexane/ethylacetate 50/1, fraction size: 25 mL).

yield: 350 mg (22 %), slightly yellowliquid, $C_7H_{11}ClO_2$, [162.61 g/mol]

R_f= 0.23 (cyclohexane/ethylacetate 50/1) ($KMnO_4$)

GC-FID (KH_80_30_280): t_R= 4.1 min

^{1}H-NMR (300.36 MHz, $CDCl_3$): δ= 5.95 (d, $^3J_{HH}$= 0.9 Hz, 1H, H-4), 4.17 (q, $^3J_{HH}$= 7.1 Hz, 2H, H-2), 4.03 (s, 2H, H-7), 2.23 (s, 3H, H-6), 1.28 (t, $^3J_{HH}$= 7.1 Hz, 3H, H-1) ppm.

^{13}C-NMR (75.53 MHz, $CDCl_3$): δ= 166.1 (C-3), 152.4 (C-5), 119.1 (C-4), 60.3 (C-2), 50.0 (C-7), 16.8 (C-6), 14.4 (C-1) ppm.

The recorded spectra are in accordance with the reported in literature.[13]

Configuration of the double bond was confirmed via the observation of an NOE effect between the proton at C-4 and the protons at C-7.

4.7.3.3 Ethyl (*E*)-4-bromo-3-methylbut-2-enoate (29)

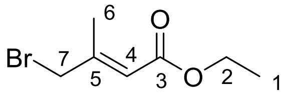

29

A 250 mL one-neck round-bottom flask was charged with ethyl (*E*)-4-hydroxy-3-methylbut-2-enoate (**27**) (6.31 g, 43.8 mmol) dissolved in 50 mL CH_2Cl_2 and was cooled using an ice/water bath. CBr_4 (19.9 g 60.0 mmol) was added and a solution of PPh_3 (15.7 g.60.0 mmol) in 150 mL CH_2Cl_2 was added dropwise to the reaction mixture at 0 °C. After 30 min stirring at 22 °C TLC indicated full conversion of starting material and the reaction mixture was concentrated to dryness using a rotary evaporator. The product was purified via flash chromatography (375 g silica gel, 15×8 cm, cyclohexane/ethylacetate 50/1, fraction size: 100 mL).

yield: 4.60 g (51 %), slightly yellow liquid, $C_7H_{11}BrO_2$, [207.07 g/mol]

R_f= 0.25 (cyclohexane/ethylacetate 50/1) ($KMnO_4$)

GC-FID (KH_80_30_280): t_R= 4.6 min

^{1}H-NMR (300.36 MHz, $CDCl_3$): δ= 5.95 (s, 1H, H-4), 4.17 (q, $^{3}J_{HH}$= 7.1 Hz, 2H, H-2), 3.94 (s, 2H, H-7), 2.27 (s, 3H, H-6), 1.28 (t, $^{3}J_{HH}$= 7.1 Hz, 3H, H-1) ppm.

^{13}C-NMR (75.53 MHz, $CDCl_3$): δ= 166.0 (C-3), 152.4 (C-5), 119.7 (C-4), 60.3 (C-2), 38.4 (C-7), 17.3 (C-6), 14.4 (C-1) ppm.

The recorded spectra are in accordance with the reported in literature.[12]

Configuration of the double bond was confirmed via the observation of an NOE effect between the proton at C-4 and the protons at C-7.

4.7.4 Synthesis of α,β-unsaturated β-aryl substituted esters

4.7.4.1 1-Phenyl-2-((tetrahydro-2*H*-pyran-2-yl)oxy)ethan-1-one (30)

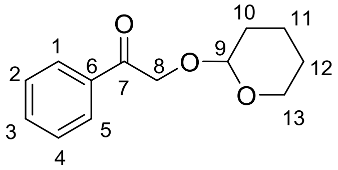

30

In a flame dried 250 mL two-neck round-bottom flask with nitrogen inlet 2-hydroxyacetophenone (2.5 g, 18.4 mmol) was dissolved in 120 mL dry CH_2Cl_2. The colorless reaction solution was cooled in an ice/water bath and Amberlyst 15 (467 mg, 12 mol%) was added. 3,4-Dihydro-2*H*-pyran (8.30 mL, 7.72 g, 91.8 mmol) was added via dropping funnel within 20 min and the reaction mixture was stirred at 0 °C for 2 h until TLC indicated complete consumption of starting materials. The reaction mixture was quenched by the addition of sat. aq. $NaHCO_3$ solution (1×100 mL) and the aqueous phase was extracted with CH_2Cl_2 (3x30 mL). The combined organic layers were washed with brine (1x100 mL), dried over Na_2SO_4, and the solvent was removed under reduced pressure.The crude product was purified via flash chromatography (500 g silica gel, 20×7.5 cm, cyclohexane/ethylacetate 20/1, fraction size: 250 mL).

yield: 2.92 g (72 %), slightly yellowliquid, $C_{13}H_{16}O_3$, [220.27 g/mol]

R_f= 0.47 (cyclohexane/ethylacetate 4/1) ($KMnO_4$)

^{1}H-NMR (300.36 MHz, $CDCl_3$): δ= 7.94 (d, $^3J_{HH}$= 7.3 Hz, 2H, H-1, H-5), 7.58 (t, $^3J_{HH}$= 7.4 Hz, 1H, H-3), 7.46 (t, $^3J_{HH}$= 7.5 Hz, 2H, H-2, H-4), 4.91 (q, $^3J_{HH}$= 17.0 Hz, 2H, H-8), 4.78 (d, $^3J_{HH}$= 3.3 Hz, 1H, H-9), 3.88 (m, 1H, H-13), 3.53 (m, 1H, H-13), 1.88 (m, 3H, H-10, H-12), 1.62 (m, 3H, H-10, H-11) ppm.

^{13}C-NMR (75.53 MHz, $CDCl_3$): δ= 196.2 (C-7), 135.2 (C-6), 133.6 (C-3), 128.8 (C-2, C-4), 128.0 (C-1, C-5), 98.8 (C-9), 69.5 (C-8), 62.4 (C-13), 30.4 (C-12), 25.5 (C-11), 19.2 (C-10) ppm.

The recorded spectra are in accordance with the reported in literature.[14]

4.7.4.2 Ethyl (*E*)-3-phenyl-4-((tetrahydro-2*H*-pyran-2-yl)oxy)but-2-enoate (31)

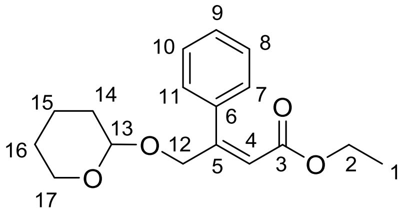

31

In a 100 mL one-neck round-bottom flask 1-phenyl-2-((tetrahydro-2*H*-pyran-2-yl)oxy)ethan-1-one (**30**) (13.3 mmol) was dissolved in 35 mL toluene. Ethyl 2-(triphenyl-λ^5-phosphanylidene)acetate (**14**) (6.0 g, 17.2 mmoL) was added in one portion and the reaction mixture was heated to 135 °C until TLC indicated full conversion of starting material after 24 h. Then the solvent was removed under reduced pressure.The crude product was purifiedvia flash chromatography (500 g silica gel, 20.0×7.5 cm, cyclohexane/ethylacetate 15/1, fraction size: 250 mL).

yield:3.24 g (84 %), slightly yellowliquid, $C_{17}H_{22}O_4$, [290.36 g/mol]

R_f= 0.42 (cyclohexane/ethylacetate 9/1) ($KMnO_4$)

^{1}H-NMR (300.36 MHz, $CDCl_3$): δ= 7.49-7.07 (m, 5H, H-7, H-8, H-9, H-10, H-11), 6.21 (s, 1H, H-4), 4.72 (d, $^3J_{HH}$= 3.1 Hz, 1H, H-13), 4.47 (dd, $^3J_{HH}$= 16.4, 1.6 Hz, 1H, H-12), 4.19 (dd, $^3J_{HH}$= 16.4, 1.6 Hz, 1H, H-12), 4.01 (q $^3J_{HH}$= 7.1 Hz, 2H, H-2), 3.83 (m, 1H, H-17), 3.52 (m, 1H, H-17), 2.0-1.5 (m, 6H, H-14, H-15, H-16), 1.07 (t, $^3J_{HH}$= 7.1 Hz, 3H, H-1) ppm.

^{13}C-NMR (75.53 MHz, $CDCl_3$): δ= 166.2 (C-3), 155.1 (C-5), 137.6 (C-6), 128.2 (C-9), 128.1 (C-8, C-9), 127.6 (C-11, C-7), 116.3 (C-4), 98.2 (C-13), 70.1 (C-12), 62.2 (C-17), 60.0 (C-2), 30.5 (C-16), 25.5 (C-15), 19.3 (C-14), 14.1 (C-1) ppm.

NMR spectra are in accordance with previously reported ones.[14]

4.7.4.3 Ethyl (*E*)-4-hydroxy-3-phenylbut-2-enoate (32)

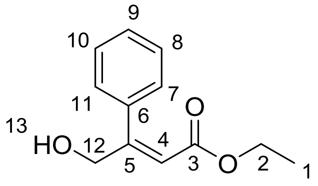

32

In a 100 mL one-neck round-bottom flask ethyl (*E*)-3-phenyl-4-((tetrahydro-2*H*-pyran-2-yl)oxy)but-2-enoate (**31**) (11.2 mmol) was dissolved in 65 mL MeOH. *p*-Toluenesulfonic acid monohydrate (106 mg, 558 µmoL) was added in one portion and the reaction mixture was stirred at22 °C until TLC indicated full conversion of starting material after 12 h. Then the solvent was removed under reduced pressureand the product was dissolved in 60 mL ethylacetate. The organic phase was washed with sat. aq. $NaHCO_3$ solution (2×50 mL) and the aqueous phase was extracted with EtOAc (3x30 mL). The combined organic layers were washed with brine (1x100 mL), dried over Na_2SO_4, and the solvent was removed under reduced pressure. The product was used in the next step without further purification

yield:2.20 g (96 %), slightly yellowliquid, $C_{12}H_{14}O_3$, [206.24 g/mol]

R_f= 0.32 (cyclohexane/ethylacetate 9/1) ($KMnO_4$)

^{1}H-NMR (300.36 MHz, DMSO-d_6): δ= 7.33 (d, $^3J_{HH}$= 3.5 Hz, 3H, H-10, H-9, H-8), 7.28-7.05(m, 2H, H-11, H-7), 6.09 (s, 1H, H-4), 5.48 (t, $^3J_{HH}$= 5.5 Hz, 1H, H-13), 4.18 (d, $^3J_{HH}$= 4.0 Hz, 2H, H-12), 3.92 (q, $^3J_{HH}$= 7.0 Hz, 1H, H-2), 1.00 (t, $^3J_{HH}$= 7.1 Hz, 3H, H-1) ppm.

^{13}C-NMR (75.53 MHz, DMSO-d_6): δ= 165.4 (C-3), 158.8 (C-6), 137.5 (C-5), 127.8 (C-10, C-9, C-8), 127.7 (C-11, C-7), 114.4 (C-4), 64.9 (C-12), 59.2 (C-2), 13.8 (C-1) ppm.

The recorded spectra are in accordance with the reported in literature.[14]

4.7.4.4 Ethyl (*E*)-4-chloro-3-phenylbut-2-enoate (33)

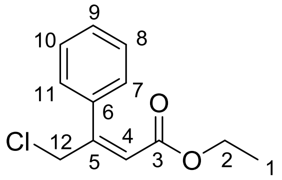

33

In a flame dried 250 mL two-neck round-bottom flask with nitrogen inlet and a bubble counterethyl (*E*)-4-hydroxy-3-phenylbut-2-enoate (**32**)(10.7 mmol) was dissolved in 30 mL dry CH_2Cl_2 and cooled to -20 °C using an acetone/dry ice bath. Dry pyridine (6.0 mL, 5.90 g, 74.7 mmol) and PPh_3 (11.20 g, 42.7 mmol) were added and the clear, colorless solution was stirred for 20 min at -20 °C. NCS (2.85 g, 21.3 mmol) was added in small portions within 60 min and the reaction solution turned dark brown. After slowly warming the reaction mixture to 22 °C (2 h), TLC indicated complete consumption of starting material. The reaction mixture was quenched by the addition of 1M HCl (100 mL). Addition of CH_2Cl_2 (100 mL) and centrifugation at 5000 rpm for 15 min produced two phases which were separated. The aqueous phase was extracted with CH_2Cl_2 (3x50 mL) and the combined organic layers were washed with 10 w% aq. $CuSO_4$ solution (3x60 mL) and brine (1x100 mL). The organic phase was dried over Na_2SO_4 and the solvent was removed under reduced pressure.The crude product was purified via flash chromatography (300 g silica gel, 12×7.5 cm, cyclohexane/ethylacetate 25/1, fraction size: 180 mL).

yield: 1.40 g (58 %), slightly yellow liquid, $C_{12}H_{13}ClO_2$, [224.86 g/mol]

R_f= 0.54 (cyclohexane/ethylacetate 9/1) ($KMnO_4$)

^{1}H-NMR (300.36 MHz, DMSO-d_6): δ= 7.56-7.50 (m, 5H, H-11, H-10, H-9, H-8, H-7), 6.32 (s, 1H, H-4), 4.63 (s, 2H, H-12), 3.94 (q, $^3J_{HH}$= 7.1 Hz, 2H, H-2), 1.01 (t, $^3J_{HH}$= 7.1 Hz, 3H, H-1) ppm.

^{13}C-NMR (75.53 MHz, DMSO-d_6): δ= 164.9 (C-3), 151.3 (C-6), 136.4 (C-5), 128.3 (C-9), 127.8 (C-11, C-10, C-8, C-7), 120.4 (C-4), 59.8 (C-2), 48.5 (C-12), 13.7 (C-1) ppm.

Configuration of the double bond was confirmed via the observation of an NOE effect between the proton at C-4 and the protons at C-12.

HRMS (EI): Calcd. (m/z) for $C_{12}H_{13}ClO_2$ [M^+]: 224.0604; found: 224.0600.

4.7.5 Synthesis of saturated aldehydes and ketones

4.7.5.1 4-Bromobutanal (34)

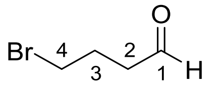

34

In a flame dried 250 mL two-neck round-bottom flask with nitrogen inlet commercially available ethyl 4-bromobutanoate (0.74 mL, 1.00 g, 5.16 mmol) was dissolved in 75 mL dry CH_2Cl_2 and was cooled to -78 °C using an acetone/dry ice bath. DIBAL-H solution (6.2 mL, 6.2 mmol, 1.0 M in CH_2Cl_2) was added dropwise to the reaction mixture in a N_2-counterstream. After full conversion of starting material was indicated by TLC and GC-FID (30 min) the reaction mixture was quenched at -78 °C by addition of 1M HCl(50 mL) and then warmed to 22 °C. The colorless layers were separated and the aqueous phase was extracted with CH_2Cl_2 (3x15 mL). The combined organic layers were washed with brine (1x50 mL) and dried over Na_2SO_4. The solvent was evaporated at650 mbar taking care that the bath temperature did not exceed 35 °C.

yield:473 mg (60 %), slightly red liquid, C_4H_7BrO, [151.00 g/mol]

R_f= 0.68 (cyclohexane/ethylacetate 4/1) ($KMnO_4$)

GC-FID (KH_80_30_280): t_R= 3.1 min

^{1}H-NMR (300.36 MHz, $CDCl_3$): δ= 9.81 (s, 1H, H-1), 3.46 (t, $^3J_{HH}$ = 6.4 Hz, 2H, H-4), 2.67 (t, $^3J_{HH}$ = 6.9 Hz, 2H, H-2), 2.36–2.07 (m, 2H, H-3) ppm.

^{13}C-NMR (75.53 MHz, $CDCl_3$): δ= 200.83 (C-1), 42.25 (C-2), 32.85 (C-4), 25.08 (C-3) ppm.

NMR spectra are in accordance with previously reported ones.[15]

4.7.5.2 4-Chlorobutanal (35)

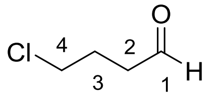

35

In a flame dried 25 mL Schlenk-flask 4-chlorobutan-1-ol (92 µL, 100 mg, 921 µmol) was dissolved in 5 mL dry CH_2Cl_2. PCC (298 mg, 1.38 mmol) and 4 Å molecular sieves (0.5 g) were added in a N_2-counterstream and the brownish solution was stirred at 22 °C. After full conversion of starting material was indicated by TLC and GC-FID, the reaction mixture was filtrated through a pad of silica and the pad rinsed with CH_2Cl_2 (3×50 mL). The solvent was evaporated at 650 mbar taking care that the bath temperature did not exceed 35 °C.

yield:65 mg (66 %), slightly yellow liquid, C_4H_7ClO, [106.55 g/mol]

R_f= 0.68 (cyclohexane/ethylacetate 4/1) ($KMnO_4$)

GC-FID (KH_80_30_280): t_R= 2.6 min

^{1}H-NMR (300.36 MHz, $CDCl_3$): δ= 9.78 (s, 1H, H-1), 3.57 (t, $^3J_{HH}$ = 6.4 Hz, 2H, H-4), 2.64 (t, $^3J_{HH}$ = 6.8 Hz, 2H, H-2), 2.24–2.07 (m, 2H, H-3) ppm.

^{13}C-NMR (75.53 MHz, $CDCl_3$): δ= 201.1 (C-1), 44.3 (C-2), 41.1 (C-4), 25.0 (C-3) ppm.

NMR spectra are in accordance with previously reported ones.[16]

4.7.5.3 5-Bromopentan-2-one (36)

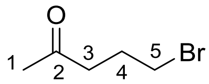

36

In a 25 mL one-neck round-bottom flask 3-acetyldihydrofuran-2(3*H*)-one (1.0 mL, 1.19 g, 9.3 mmol) was dissolved in 4 mL toluene. HBr (48 w% in water, 1.6 mL, 2.73 g, 14.0 mmol) was added in one portion and the reaction mixture was heated to 80 °C until TLC indicated full conversion of the staring material(8 h). The reaction solution was diluted by the addition of distilled H_2O (10 mL), the phases were separated, and the aqueous phase was extracted with Et_2O (6x10 mL). The combined organic layers were washed with brine (1x60 mL), dried over Na_2SO_4, and the solvent was removed under reduced pressure. The brown oil was

purified via flash chromatography (125 g silica gel, 12×4.0 cm, cyclohexane/ethylacetate4/1, fraction size: 50 mL).

yield:791 mg (52 %), orangeliquid, C_5H_9BrO, [165.03 g/mol]

R_f= 0.62 (cyclohexane/ethylacetate4/1) ($KMnO_4$)

GC-FID (KH_80_30_280): t_R= 3.6 min

^{1}H-NMR (300.36 MHz, $CDCl_3$): δ= 3.44 (t, $^3J_{HH}$= 6.3 Hz, 2H, H-5), 2.64 (t, $^3J_{HH}$= 6.9 Hz, 2H, H-3), 2.12 (m, 5H, H-1, H-4) ppm.

^{13}C-NMR (75.53 MHz, $CDCl_3$): δ= 207.5 (C-2), 41.6 (C-3), 33.4 (C-5), 30.2 (C-1), 26.5 (C-4) ppm.

HRMS (EI): Calcd. (m/z) for C_3H_6Br [M-CH_3CO]: 120.9653; found: 120.9653.

NMR spectra are in accordance with previously reported ones.[17]

4.7.5.4 Ethyl 4-hydroxy-3-methylbutanoate (37)

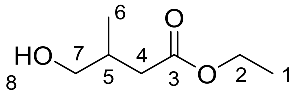

37

In a 250 mL two-neck round-bottom flask with nitrogen inletethyl (*E*)-4-hydroxy-3-methylbut-2-enoate(**27**) (682 mg, 4.73 mmol) was dissolved in 160 mL EtOAc. Et_3N (394 µL, 288 mg, 2.85 mmol) and 10 % Pd/C (160 mg, 150 µmol) were added. The grey suspension was cooled to 0 °C and after providinghydrogen atmosphere by evacuating and back-flushing with hydrogen gas (3 x), the reaction mixture was stirred for 3 h at 0 °C with an attached balloon filled with H_2. Complete consumption of starting material was detected by GC-FID indicating the formation of anunknown byproduct (50 %). The reaction solution was filtrated through a pad of celite, which was rinsed with ethylacetate (3×30 mL), and the solvent was evaporated under reduced pressure. The crude product was used immediately in the next step without further purification.

4.7.5.5 Ethyl 4-chloro-3-methylbutanoate (38)

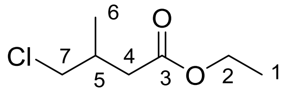

38

In a flame dried 100 mL two-neck round-bottom flask with nitrogen inlet and a bubble counterethyl 4-hydroxy-3-methylbutanoate (**37**) (998 mg, 6.83 mmol) was dissolved in 30 mL dry CH_2Cl_2 and cooled to -20 °C using an acetone/dry ice bath. Dry pyridine(4.0 mL, 3.84 g, 49.0 mmol) and PPh_3 (7.28 g, 27.7 mmol) were added and the clear, colorless solution was stirred for 20 min at -20 °C. NCS (1.85 g, 13.8 mmol) was added in small portions within 30 min and the reaction solution turned dark brown. After slowly warming the reaction mixture to 22 °C (2 h), TLC indicated complete consumption of starting material. The reaction mixture was quenched by the addition of 1M HCl (40 mL). The aqueous phase was extracted with CH_2Cl_2 (3x50 mL) and the combined organic layers were washed with 10 w% aq. $CuSO_4$ solution (3x60 mL) and brine (1x100 mL). The organic phase was dried over $Na_2SO_4$and the solvent was removed under reduced pressure. The product was purified via flash chromatography (500 g silica gel, 20×8 cm, cyclohexane/ethylacetate 70/1, fraction size: 250 mL).

yield: 653 mg (3.91 mmol, 58 %), colorless liquid, $C_7H_{13}O_2Cl$, [164.63]

R_f= 0.61 (cyclohexane/ethylacetate 10/1, UV and $KMnO_4$)

^{1}H NMR(300.36 MHz, $CDCl_3$):δ = 4.14 (q,$^3J_{HH}$=7.1 Hz, 2H, H-2), 3.75-3.27 (m, 2H, H-7), 2.53 (dd, $^3J_{HH}$= 15.2, 6.0 Hz, 1H, H-4), 2.44-2.27 (m, 1H, H-5), 2.24 (dd, $^3J_{HH}$=15.2, 7.2 Hz, 1H, H-4), 1.26 (t, $^3J_{HH}$= 7.1 Hz, 3H, H-1), 1.07 (d, $^3J_{HH}$= 6.6 Hz, 3H, H-6) ppm.

^{13}C NMR (75.53 MHz, $CDCl_3$):δ = 172.4 (C-3), 60.6 (C-2), 50.3 (C-7), 38.6 (C-4), 32.7 (C-5), 17.9 (C-6), 14.4 (C-1) ppm.

HRMS (EI): Calcd. (m/z) for $C_7H_{12}O_2$ [M-HCl]: 128.0837, found: 128.0837.

4.7.5.6 Ethyl 4-hydroxy-2-methylbutanoate (39)

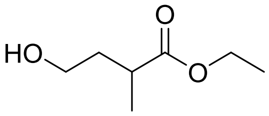

39

The preparation was executed as described in 4.7.5.4 starting from ethyl (*E*)-4-hydroxy-2-methylbut-2-enoate (**24**) (1.0 g, 7.0 mmol). The crude product was used immediately in the next step without further purification.

4.7.5.7 Ethyl 4-chloro-2-methylbutanoate (40)

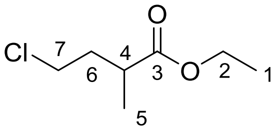

40

The preparation was executedas described in 4.7.5.5starting from ethyl 4-hydroxy-2-methylbutanoate(**39**) (1.25 g, 8.55 mmol). The product was purified via flash chromatography (500 g silica gel, 22×7.5 cm, cyclohexane/ethylacetate 70/1, fraction size: 250 mL).

yield: 865 mg (5.25 mmol, 61 %), colorless liquid, $C_7H_{13}O_2Cl$, [164.63]

R_f= 0.67 (cyclohexane/ethylacetate 10/1, UV and $KMnO_4$)

^{1}H NMR (300.36 MHz, $CDCl_3$):δ = 4.14 (q, $^3J_{HH}$=7.1 Hz, 2H, H-2), 3.56 (t, $^3J_{HH}$=6.7 Hz, 2H, H-7), 2.83-2.45 (m, 1H, H-4), 2.18 (td, $^3J_{HH}$=14.3, 6.6 Hz, 1H, H-6), 1.83 (dt, $^3J_{HH}$= 13.4, 6.7 Hz, 1H, H-6), 1.41-0.99 (m, 6H, H-1, H-5) ppm.

^{13}C NMR (75.53 MHz, $CDCl_3$):δ = 175.9 (C-3), 60.7 (C-2), 42.7 (C-7), 36.9 (C-4), 36.2 (C-6), 17.0 (C-5), 14.3 (C-1) ppm.

NMR spectra are in accordance with previously reported ones.[18]

4.7.5.8 Ethyl 4-bromo-3-methylbutanoate (41)

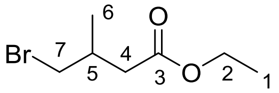

41

A 250 mL one-neck round-bottom flask was charged with ethyl 4-hydroxy-3-methylbutanoate(**37**) (557 mg, 3.81 mmol) dissolved in 5 mL $CH_2Cl_2$and cooled in an ice/water bath.CBr_4 (1.98 g 6.0 mmol) was added and a solution of PPh_3(1.20 g, 4.57 mmol) in 5 mL $CH_2Cl_2$was added dropwise to the reaction mixture at 0 °C. After 30 min stirring at 22 °C TLC indicated full conversion of starting material and the reaction mixture was concentrated to dryness without further workup. The product was purified via flash chromatography(200 g silica gel, 6×14 cm, cyclohexane/ethylacetate 70/1, fraction size: 120 mL).

yield: 536 mg (67 %), colourlessliquid, $C_7H_{13}BrO_2$, [209.08 g/mol]

R_f= 0.45 (cyclohexane/ethylacetate 10/1) ($KMnO_4$)

GC-FID (KH_80_30_280): t_R= 4.0 min

^{1}H NMR(300.36 MHz, $CDCl_3$):δ = 4.38-391 (m, 2H, H-2), 3.63-3.25 (m, 2H, H-7), 2.52 (dd, $^3J_{HH}$= 14.9, 5.7 Hz, 1H, H-4), 2.43-2.15 (m, 2H, H-4, H-5), 1.26 (t, $^3J_{HH}$= 7.1 Hz, 3H, H-1), 1.08 (d, $^3J_{HH}$= 6.4 Hz, 3H, H-6) ppm.

^{13}C NMR (75.53 MHz, $CDCl_3$):δ = 172.3 (C-3), 60.6 (C-2), 40.3 (C-7), 39.5 (C-4), 32.3 (C-5), 18.9 (C-6), 14.4 (C-1) ppm.

NMR spectra are in accordance with previously reported ones.[19]

4.7.5.9 Ethyl 4-hydroxy-3-phenylbutanoate (42)

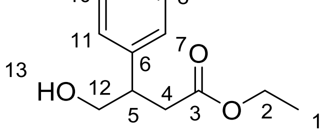

42

A 0.05 M solution of ethyl (*E*)-4-hydroxy-3-phenylbut-2-enoate (**32**) (1.14 g, 5.53 mmol) in 110 mL methanol was prepared in a 200 mL Erlenmeyer flask. The reduction was carried out

using a continuous-flow hydrogenation reactor (H-cube™) with a 10 % Pd/C catalyst cartridge with the following conditions: 1 mL/min, 60 °C, 60 bar H_2. The product solution was collected in a 250 mL Erlenmeyer flask and the solvent was removed under reduced pressure. The product was purified via flash chromatography (50 g silica gel, 11×3.5 cm, cyclohexane/ethylacetate4/1, fraction size: 25 mL).

yield: 536 mg (67 %), colorlessliquid, $C_{12}H_{16}O_3$, [208.26 g/mol]

R_f= 0.45 (cyclohexane/ethylacetate10/1) ($KMnO_4$)

^{1}H-NMR (300.36 MHz, MeOD): δ= 7.38-7.08 (m, 5H, H-7, H-8, H-9, H-10, H-11), 4.00 (q, $^3J_{HH}$= 7.1 Hz, 2H, H-2), 3.77-3.57 (m, 2H, H-12), 3.27 (dd, $^3J_{HH}$= 10.8, 4.9 Hz, 1H, H-5), 2.87 (dd, $^3J_{HH}$= 15.4, 5.8 Hz, 1H, H-4), 2.59 (dd, $^3J_{HH}$= 15.3, 9.4 Hz, 1H, H-4), 1.11 (t, $^3J_{HH}$= 7.1 Hz, 3H, H-1) ppm.

^{13}C-NMR (75.53 MHz, MeOD): δ= 164.8 (C-3), 133.3 (C-6), 120.0 (C-7, C-11), 119.5 (C-8, C-10), 118.3 (C-9), 57.7 (C-12), 51.9 (C-2), 36.9 (C-5), 28.9 (C-4), 4.9 (C-1) ppm.

HRMS (EI): Calcd. (m/z) for $C_{12}H_{16}O_3$ [M^+]: 208.1099; found: 208.1091.

4.7.5.10 Ethyl 4-chloro-3-phenylbutanoate (43)

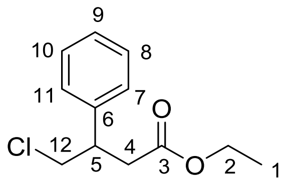

43

In a flame dried 250 mL two-neck round-bottom flask with nitrogen inlet and a bubble counterethyl 4-hydroxy-3-phenylbutanoate (**42**)(747 mg, 3.59 mmol)was dissolved in 14 mL dry CH_2Cl_2 and cooled to -20 °C using an acetone/dry ice bath. Dry pyridine(2.0 mL, 1.99 g, 24.8 mmol) and PPh_3 (3.76 g, 14.3 mmol) were added and the clear, colorless solution was stirred for 20 min at -20 °C. NCS (957 mg, 7.17 mmol) was added in small portions within 30 min and the reaction solution turned dark brown. After slowly warming the reaction mixture to 22 °C (2 h), TLC indicated complete consumption of starting material. The reaction mixture was quenched by the addition of 1M HCl (20 mL). Addition of CH_2Cl_2 (20 mL) and centrifugation at 5000 rpm for 15 min produced two phases which were separated. The aqueous phase was extracted with CH_2Cl_2 (3x20 mL) and the combined

organic layers were washed with 10 w% aq. $CuSO_4$ solution (3x20 mL) and brine (1x50 mL). The organic phase was dried over $Na_2SO_4$and the solvent was removed under reduced pressure. The product was purified via flash chromatography (250 g silica gel, 17.5×5.5 cm, cyclohexane/ethylacetate 25/1, fraction size: 150 mL).

yield: 1.40 g (58 %), slightly yellowliquid, $C_{12}H_{15}ClO_2$, [226.70 g/mol]

R_f= 0.56 (cyclohexane/ethylacetate 9/1) ($KMnO_4$)

GC-FID (KH_80_30_280): t_R= 6.3 min

^{1}H-NMR (300.36 MHz, DMSO-d_6): δ= 7.30-7.26 (m, 5H, H-7, H-8, H-9, H-10, H-11), 3.99-3.79 (m, 4H, H-2, H-12), 3.40 (dd, $^3J_{HH}$= 14.1, 7.5 Hz 2H, H-5), 2.89 (dd, $^3J_{HH}$= 15.8, 5.9 Hz, 1H, H-4), 2.69 (dd, $^3J_{HH}$= 15.8, 9.0 Hz, 1H, H-4), 1.05 (t, $^3J_{HH}$= 7.1 Hz, 3H, H-1) ppm.

^{13}C-NMR (75.53 MHz, DMSO-d_6): δ= 171.0 (C-3), 140.6 (C-6), 128.3 (C-8, C-10), 127.8 (C-7, C-11), 127.0 (C-9), 59.9 (C-2), 48.7 (C-12), 43.8 (C-5), 37.5 (C-4), 13.9 (C-1) ppm.

HRMS (EI): Calcd. (m/z) for $C_{12}H_{14}O_2$ [M-HCl]: 190.0994; found: 190.0996.

4.7.5.11 General Procedure for the reduction of saturated esters (38, 40, 41, 43) to the corresponding aldehydes (6-Cl, 6-Br, 46, 9)

In a flame dried 50 mL two-neck round-bottom flask with nitrogen inlet, saturated ester **38**, **40**, **41**, **43** (1.48 mmol) was dissolved in 5 mL dry CH_2Cl_2. The reaction solution was cooled to -78 °C using an acetone/dry ice bath. A solution of DIBAL-H (1.0 M in CH_2Cl_2, 1.6 mL, 1.6 mmol) was added dropwise via syringe to the reaction mixture in a N_2-counterstream. After 15 min TLC indicated complete consumption of the starting material and overreduction to the alcohol. The reaction mixture was quenched by addition of 1M HClat -78 °C and then warmed to 22 °C. The colorless layers were separated and the aqueous phase was extracted with CH_2Cl_2 (2 x 50 mL). The combined organic layers were washed with brine (1x100 mL) and dried over Na_2SO_4. The reaction solution was added to a flame dried 50 mL two-neck round-bottom flask with nitrogen inlet. PCC (487 mg, 2.22 mmol) and 4 Å molecular sieves (0.5 g) were added in a N_2-counterstream and the brownish solution was stirred at 22 °C. After full conversion was indicated by TLC and GC-FID, the reaction mixture was filtrated through a pad of silica, which was then rinsed with CH_2Cl_2 (3×50 mL). The solvent was evaporated under reduced pressure.

4.7.5.12 4-Bromo-3-methylbutanal (6-Br)

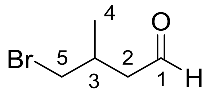

6-Br

Starting from ethyl 4-bromo-3-methylbutanoate(**41**) (294 mg, 1.41 mmol) reduction to the aldehyde was executed as described in4.7.5.11. The product did not require further purification.

yield: 172 mg (74 %), slightly green liquid, C_5H_9BrO, [165.03 g/mol]

GC-FID (KH_80_30_280): t_R= 3.4 min

R_f= 0.27 (cyclohexane/ethylacetate 10/1) ($KMnO_4$)

^{1}H-NMR (300.36 MHz, $CDCl_3$): δ= 9.78 (s, 1H, H-1), 3.42 (ddd, $^3J_{HH}$= 27.7, 10.0 5.1 Hz, 2H, H-5), 2.81-2.60 (m, 1H, H-2), 2.55-2.24 (m, 2H, H-2, H-3), 1.08 (d, $^3J_{HH}$= 6.5 Hz,3H, H-4) ppm.

^{13}C-NMR (75.53 MHz, $CDCl_3$): δ= 201.0 (C-1), 48.9 (C-2), 40.2,(C-5), 30.0 (C-3), 19.1 (C-4) ppm.

HRMS (EI): Calcd. (m/z) for C_5H_8O [M-HBr]: 84.0575; found: 84.0574.

4.7.5.13 4-Chloro-3-methylbutanal (6-Cl)

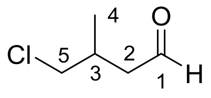

6-Cl

Starting from ethyl 4-chloro-3-methylbutanoate (**38**) (400 mg, 2.43 mmol) reduction to the aldehyde was executed as described in4.7.5.11. The product was purified via flash chromatography (40 g silica gel, 9.0×4.5 cm, *n*-pentane/diethylether 20/1, fraction size: 25 mL).

yield: 59 mg (20 %), colourless liquid, C_5H_9ClO, [120.58 g/mol]

R_f= 0.26 (cyclohexane/ethylacetate 10/1) ($KMnO_4$)

GC-FID (KH_80_30_280): t_R= 2.9 min

^{1}H-NMR (300.36 MHz, $CDCl_3$): δ= 9.78 (s, 1H, H-1), 3.52 (ddd, $^3J_{HH}$= 29.6, 10.8 5.3 Hz, 2H, H-5), 2.71 (dd, $^3J_{HH}$= 16.7, 5.0 Hz, 1H, H-2), 2.63-2.20 (m, 2H, H-2, H-3), 1.07 (d, $^3J_{HH}$= 6.6 Hz,3H, H-4) ppm.

^{13}C-NMR (75.53 MHz, $CDCl_3$): δ= 201.1 (C-1), 50.3 (C-5), 48.0,(C-2), 30.4 (C-3), 18.1 (C-4) ppm.

HRMS (EI): Calcd. (m/z) for C_5H_8O [M-HCl]: 84.0575; found: 84.0575.

4.7.5.14 4-Chloro-2-methylbutanal (46)

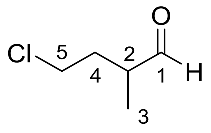

46

Starting from ethyl 4-chloro-2-methylbutanoate (**40**) (400 mg, 2.43 mmol)reduction to the aldehyde was executed as described in4.7.5.11.The product was purified via flash

chromatography (25 g silica gel, 7.0×2.5 cm, *n*-pentane/diethylether20/1, fraction size: 25 mL).

yield: 20 mg (7 %), colourless liquid, C_5H_9ClO, [120.58 g/mol]

R_f= 0.64 (cyclohexane/ethylacetate 2/1) ($KMnO_4$)

GC-FID (KH_80_30_280): t_R= 2.9 min

^{1}H-NMR (300.36 MHz, $CDCl_3$): δ= 9.68 (s, 1H, H-1), 3.78-3.45 (m, 2H, H-5), 2.67 (dq, $^3J_{HH}$= 13.8, 6.9 Hz, 1H, H-2), 2.24 (td, $^3J_{HH}$= 13.7, 6.7 Hz, 1H, H-4), 1.88-1.65 (m, 1H, H-4), 1.16 (d, $^3J_{HH}$= 7.2 Hz, 3H, H-3) ppm.

^{13}C-NMR (75.53 MHz, $CDCl_3$): δ= 203.7 (C-1), 43.7 (C-2), 42.2, (C-5), 33.0 (C-4), 13.1 (C-3) ppm.

NMR spectra are in accordance with previously reported ones.[18]

4.7.5.15 4-Chloro-3-phenylbutanal (9)

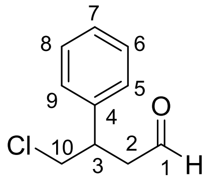

9

Starting from ethyl 4-chloro-3-phenylbutanoate (**43**) (336 mg, 1.48 mmol) reduction to the aldehyde was executed as described in4.7.5.11. The product was purified via flash chromatography (15 g silica gel, 4.5×2.0 cm, cyclohexane/ethylacetate 12/1, fraction size: 10 mL).

yield: 114 mg (42 %), slightly yellowliquid, $C_{10}H_{11}ClO$, [182.65 g/mol]

R_f= 0.41 (cyclohexane/ethylacetate 4/1) ($KMnO_4$)

GC-FID (KH_80_30_280): t_R= 5.9 min

^{1}H-NMR (300.36 MHz, DMSO-d_6): δ= 9.60 (s, 1H, H-1), 7.33-7.24 (m, 5H, H-5, H-6, H-7, H-8, H-9), 4.02-3.71 (m, 2H, H-10), 3.72-3.39 (m, 1H, H-3), 3.16-2.66 (m, 2H, H-2) ppm.

^{13}C-NMR (75.53 MHz, DMSO-d_6): δ= 201.7 (C-1), 140.9 (C-4), 128.4 (C-6, C-8), 127.8 (C-5, C-9), 127.0 (C-7), 49.0 (C-10), 46.4 (C-2), 41.3 (C-3) ppm.

HRMS (EI): Calcd. (m/z) for $C_{10}H_{10}O$ [M-HCl]: 146.0732; found: 146.0736.

4.7.6 Synthesis of racemic and chiral cyclopropanes

4.7.6.1 1-Methylcyclopropane-1-carbaldehyde (48)

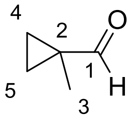

48

In a flame dried 50 mL two-neck round-bottom flask with nitrogen inlet (1-methylcyclopropyl)methanol (250 mg, 2.9 mmol) was dissolved in 18 mL abs. CH_2Cl_2. PCC (1.25 g, 5.80 mmol) and 4 Å molecular sieves (0.5 g) were added in a N_2-counterstream and the brownish solution was stirred at 22 °C. After full conversion of starting material was indicated by GC-FID (8 h), the reaction mixture was filtrated through a pad of silica, which was then rinsed with CH_2Cl_2 (3×50 mL). The clear and colorless filtrate was concentrated at a pressure of 200 mbar (with care to not exceed 35 °C water bath temperature).

yield: 61 mg (25 %), colorless liquid, C_5H_8O, [84.12 g/mol]

GC-FID (KH_80_30_280): t_R= 1.8 min

^{1}H-NMR (300.36 MHz, $CDCl_3$): δ = 8.63 (s, 1H, H-5), 5.29 (s, 3H, H-3), 1.19-1.08 (m, 2H, H-5), 0.92 (q, $^3J_{HH}$= 4.1 Hz, 2H, H-4) ppm.

^{13}C-NMR (75.53 MHz, $CDCl_3$): δ = 202.1 (C-1), 27.7 (C-2), 15.7 (C-3), 14.0 (C-4, C-5) ppm.

The recorded spectra are in accordance with the reported in literature.[20]

4.7.6.2 *trans*-(2-Phenylcyclopropyl)methanol (*rac*)(*trans*-49)

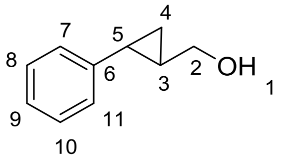

trans-**49**

In a flame dried 50 mL three-neck round-bottom flask equipped with nitrogen inlet and a bubble counter,freshly distilled CH_2I_2(360 µL, 1.20 g, 4.47 mmol) wasdissolved in 26 mL dry CH_2Cl_2 (0.17 M). The reaction mixture was cooled to 0 °C using an ice/water bath and Et_2Zn

(1.0 M solution in hexanes, 2.8 mL, 2.8 mmol) was added dropwise via syringe forming a colorless precipitate. In the meanwhile, in a second flame dried 25 mL Schlenk flask (*E*)-3-phenylprop-2-en-1-ol (300 mg, 2.24 mmol) was dissolved in 6.3 mL dry CH_2Cl_2 (0.33 M). The reaction mixture was cooled to 0 °C using an ice/water bath and Et_2Zn (1.0 M solution in hexanes, 2.8 mL, 2.8 mmol) was added dropwise. After 30 min stirring at 0 °C the content of the second flask was added to the first flask via cannula. The reaction solution was stirred at 22 °C until GC-FID indicated complete consumption of starting material (8 h). The reaction was quenched by the addition of sat. aq. NH_4Cl-solution (26 mL, 11.5 mL/mmolallylic alcohol). The aqueous phase was extracted with CH_2Cl_2 (3x15 mL) and the combined organic layers were washed with brine (1x50 mL), dried over Na_2SO_4, and the solvent was evaporated under reduced pressure. The product was purified via flash chromatography (30 g silica gel, 7×3.5 cm, cyclohexane/ethylacetate 5/1, fraction size: 15 mL).

yield: 160 mg (48 %), colourlessliquid, $C_{10}H_{12}O$, [148.21 g/mol]

R_f= 0.24 (cyclohexane/ethylacetate 5/1) ($KMnO_4$)

^{1}H-NMR (300.36 MHz, $CDCl_3$): δ= 7.24-7.05 (m,5H, H-7, H-8, H-9, H-10, H-11), 3.60 (m, 2H, H-2), 1.80 (m, 1H, H-5), 1.58 (s, 1H, H-1), 1.43 (m, 1H, H-3), 0.93 (m, 2H, H-4) ppm.

^{13}C-NMR (75.53 MHz, $CDCl_3$): δ= 142.6(C-6), 128.5 (C-7, C-11), 126.0 (C-8, C-10), 125.8 (C-9), 66.7 (C-2), 25.4 (C-3), 21.4 (C-5), 13.9 (C-4) ppm.

The recorded spectra are in accordance with the reported in literature.[21]

4.7.6.3 *trans*-2-Phenylcyclopropane-1-carbaldehyde (*rac*) (*trans*-10)

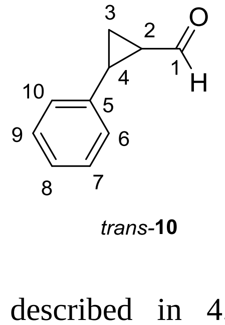

trans-**10**

The preparation was executed as described in 4.7.6.1starting from racemic*trans*-(2-phenylcyclopropyl)methanol(*trans*-**49**) (100 mg, 675 µmol).

yield: 53 mg (53 %), yellowish liquid, $C_{10}H_{12}O$, [148.21 g/mol]

GC-FID (KH_80_30_280): t_R= 5.2 min

^{1}H-NMR (300.36 MHz, $CDCl_3$): δ = 9.33 (d, $^3J_{HH}$= 4.6 Hz, 1H, H-1), 7.33-7.11 (m, 5H, H-6, H-7, H-8, H-9, H-10), 2.66-2.61 (m, 1H, H-4), 2.21-2.15 (m, 1H, H-2), 1.76-1.71 (m, 1H, H-3), 1.56-1.51 (m, 1H, H-3) ppm.

^{13}C-NMR (75.53 MHz, $CDCl_3$): δ = 199.6 (C-1), 138.9 (C-5), 128.5 (C-6, C-10), 126.7 (C-7, C-9), 126.2 (C-8), 33.7 (C-2), 26.5 (C-4), 16.4 (C-3) ppm.

The recorded spectra are in accordance with the reported in literature.[22]

4.7.6.4 (*Z*)-2-Phenylethen-1-ol (51)

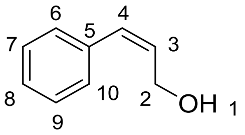

51

In a 20 mL two-neck round-bottom flask equipped with nitrogen inlet3-phenylprop-2-yn-1-ol (1.0 mL, 1.08 g, 8.16 mmol), quinoline (102 µL, 111 mg, 860 µmol), and Lindlar catalyst (208 mg, 1.96 mmol) were suspended in 12 mL ethylacetate.After ensuring hydrogen atmosphere by evacuating and back-flushing with hydrogen gas (3 x), the reaction mixture was stirred at22 °C with an attached balloon filled with H_2 until GC-FID indicated complete consumption of starting material (1 h). The reaction solution was filtrated through a pad of celite, which was rinsed with ethylacetate (2×8 mL), and the solvent was evaporated under reduced pressure. The product was purified via flash chromatography (100 g silica gel, 12×1.5 cm, cyclohexane/ethylacetate 5/1, fraction size: 50 mL).

yield: 760 mg (69 %), colorlessliquid, $C_9H_{10}O$, [134.18 g/mol]

R_f= 0.23 (cyclohexane/ethylacetate 5/1) ($KMnO_4$)

^{1}H-NMR (300.36 MHz, $CDCl_3$): δ= 7.31-7.13 (m,5H, H-6, H-7, H-8, H-9, H-10), 6.51 (d, $^3J_{HH}$= 11.7 Hz, 1H, H-4), 5.81 (dt, $^3J_{HH}$= 11.7, 6.3 Hz, 1H, H-3), 4.37 (dd, $^3J_{HH}$= 6.3, 1.1 Hz, 2H, H-2), 1.62 (s, 1H, H-1) ppm.

^{13}C-NMR (75.53 MHz, $CDCl_3$): δ= 136.7(C-5), 131.3 (C-3), 131.2 (C-4), 128.9 (C-6, C-10), 128.4 (C-7, C-9), 127.4 (C-8), 59.8 (C-2) ppm.

The recorded spectra are in accordance with the reported in literature.[23]

4.7.6.5 *cis*-(2-Phenylcyclopropyl)methanol (*rac*) (*cis*-49)

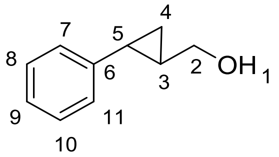

(*cis*-**49**)

Starting from (*Z*)-2-phenylethen-1-ol (**51**) (760 mg, 5.66 mmol) cyclopropanation was executed as described in 4.7.6.2. The product was purified via flash chromatography (100 g silica gel, 12×1.5 cm, cyclohexane/ethylacetate 5/1, fraction size: 50 mL).

yield: 584 mg (70 %), colorlessliquid, $C_{10}H_{12}O$, [148.21 g/mol]

R_f= 0.24 (cyclohexane/ethylacetate 5/1) ($KMnO_4$)

^{1}H-NMR (300.36 MHz, $CDCl_3$): δ= 7.28-7.15 (m,5H, H-7, H-8, H-9, H-10, H-11), 3.43 (dd, $^3J_{HH}$= 11.6, 6.4 Hz, 1H, H-2), 3.24 (dd, $^3J_{HH}$= 11.6, 8.5 Hz, 1H, H-2), 2.27 (dd, $^3J_{HH}$= 14.7, 8.3 Hz, 1H, H-3), 1.48-1.37 (m, 2H, H-1, H-5), 1.02 (td, $^3J_{HH}$= 8.3, 5.4 Hz, 1H, H-4), 0.85 (dd, $^3J_{HH}$= 11.2, 5.6 Hz, 1H, H-4) ppm.

^{13}C-NMR (75.53 MHz, $CDCl_3$): δ= 138.4(C-6), 129.0 (C-7, C-11), 128.4 (C-8, C-10), 126.3 (C-9), 63.0 (C-2), 21.0 (C-3), 20.8 (C-5), 7.8 (C-4) ppm.

The recorded spectra are in accordancewith the reported in literature.[24]

4.7.6.6 *cis*-2-Phenylcyclopropane-1-carbaldehyde (*rac*) (*cis*-10)

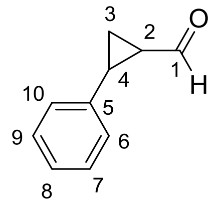

cis-**10**

The preparation was executed as described in 4.7.6.1starting from racemic *cis*-(2-phenylcyclopropyl)methanol (*cis*-**49**) (108 mg, 729 µmol).

yield: 65 mg (60 %), yellowish liquid, $C_{10}H_{12}O$, [148.21 g/mol]

GC-FID (KH_80_30_280): t_R= 5.3 min

^{1}H-NMR (300.36 MHz, $CDCl_3$): δ = 8.66 (d, $^3J_{HH}$= 6.6 Hz, 1H, H-1), 7.32-7.21 (m, 5H, H-6, H-7, H-8, H-9, H-10), 2.83 (q, $^3J_{HH}$= 8.2 Hz, H-2), 2.14-2.00 (m, 1H, H-4), 1.88-1.79 (dt, $^3J_{HH}$= 7.2, 5.3 Hz, 1H, H-3), 1.64-1.55 (dt, $^3J_{HH}$= 8.2, 5.6 Hz 1H, H-3) ppm.

^{13}C-NMR (75.53 MHz, $CDCl_3$): δ = 201.3 (C-1), 135.7(C-5), 129.1 (C-6, C-10), 128.5 (C-7, C-9), 127.1 (C-8), 29.6 (C-4), 26.3 (C-2), 11.5 (C-3) ppm.

The recorded spectra are in accordance with the reported in literature.[25]

4.7.6.7 (*Z*)-But-2-en-1-ol (*cis*-52)

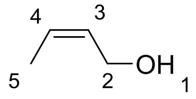

cis-**52**

In a 250 mL two-neck round-bottom flask equipped with nitrogen inlet 2-butyn-1-ol (5.0 mL, 4.69 g, 66.8 mmol) and Lindlar catalyst (133 mg, 1.25 mmol) were suspended in 100 mL MeOH. After ensuring hydrogen atmosphere by evacuating and back-flushing with hydrogen gas (3 x), the reaction mixture was stirred at22 °C with an attached balloon filled with H_2 until GC-FID indicated complete consumption of starting material (3 d). The reaction solution was filtrated through a pad of celite, which was then rinsed withMeOH (3×50 mL).The filtrate was concentrated at a pressure of 200 mbar (with care to not exceed 35 °C water bath temperature). The yellow liquid was purified by distillation at atmospheric pressure (b.p. = 112 °C).

yield: 2.07 g (43 %), colourlessliquid, C_4H_8O, [72.06 g/mol]

GC-FID (KH_80_30_280): t_R= 1.8 min

^{1}H NMR (300.36 MHz, $CDCl_3$): δ= 5.72–5.47 (m, 2H, H-3, H-4), 4.20 (d, $^3J_{HH}$= 4.3 Hz, 2H, H-2), 1.69–1.63 (m, 3H, H-5), 1.57 (s, 1H, H-1) ppm.

^{13}C-NMR (75.53 MHz, $CDCl_3$): δ= 129.4(C-4), 127.3 (C-3), 58.4 (C-2), 13.1 (C-5) ppm.

The recorded spectra are in accordance with the reported in literature.[26]

4.7.6.8 (*E*)-But-2-en-1-ol (*trans*-52)

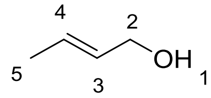

trans-**52**

In a flame dried 250 mL two-neck round-bottom flask equipped with nitrogen inlet and bubble counter, $LiAlH_4$ (5.32 g, 140 mmol) was suspended in 25 mL dry Et_2O at 0 °C. A solution of 2-butyn-1-ol (5.0 mL, 4.69 g, 66.8 mmol) in 25 mL dry Et_2O was added dropwise to the reaction mixture and the grey suspension was stirred at 22 °C until GC-FID indicated complete consumption of starting material (8 h). The reaction mixture was cooled using an ice/water bath and dist. H_2O (5.3 mL), 15 w% NaOH (5.3 mL), and dist. H_2O (15.9 mL) were added carefully in the stated sequence. After the reaction mixture was stirred at 22 °C for 30 min the colorless precipitate was removed by filtration through a fritted funnel, which was washed with Et_2O (3×30 mL).The filtrate was concentrated at a pressure of 200 mbar (with care to not exceed 35 °C water bath temperature). The yellow solution was purified by distillation at atmospheric pressure (b.p. = 114 °C).

yield: 2.97 g (62 %), colourlessliquid, C_4H_8O, [72.06 g/mol]

GC-FID (KH_80_30_280): t_R= 1.7 min

^{1}H NMR (300.36 MHz, $CDCl_3$): δ= 5.98–5.35 (m, 2H, H-3, H-4), 4.05 (d, $^3J_{HH}$=3.8 Hz, 2H, H-2), 1.70 (d, $^3J_{HH}$= 4.9 Hz, 3H, H-5), 1.60 (s, 1H, H-1) ppm.

^{13}C-NMR (75.53 MHz, $CDCl_3$): δ= 130.3(C-4), 128.2 (C-3), 63.8 (C-2), 17.8 (C-5) ppm.

The recorded spectra are in accordance with the reported in literature.[26]

4.7.6.9 *cis*-(2-Methylcyclopropyl)methanol (*rac*) (*cis*-53)

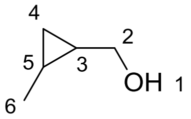

cis-**53**

Starting from (*Z*)-but-2-en-1-ol(*cis*-**52**) (500 mg, 6.94 mmol) cyclopropanation was executed as described in4.7.6.2. The product was purified via flash chromatography (100 g silica gel, 12×5 cm, *n*-pentane/diethylether4/1, fraction size: 25 mL).

yield:155 mg (26 %), colourlessliquid, $C_5H_{10}O$, [86.13 g/mol]

R_f= 0.33 (cyclohexane/ethylacetate 4/1) (CAM)

GC-FID (KH_80_30_280): t_R= 2.1 min

^{1}H-NMR (300.36 MHz, $CDCl_3$): δ= 3.89–3.63 (m, 1H, H-2), 3.63–3.35 (m, 1H, H-2), 1.36 (s, 1H, H-1), 1.07 (dd, $^3J_{HH}$= 14.2, 6.4 Hz, 4H, H-6, H-5), 1.00–0.85 (m, 1H, H-3), 0.81–0.46 (m, 1H, H-4), -0.07 (dd, , $^3J_{HH}$= 10.0, 5.0 Hz, 1H, H-4) ppm.

^{13}C-NMR (75.53 MHz, $CDCl_3$): δ= 63.3(C-2), 18.2 (C-5), 13.2 (C-6), 10.7 (C-4), 9.8 (C-3) ppm.

The recorded spectra are in accordance with the reported in literature.[27]

4.7.6.10 *trans*-(2-Methylcyclopropyl)methanol (*rac*) (*trans*-53)

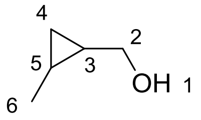

trans-**53**

Starting from (*E*)-but-2-en-1-ol(*trans*-**52**) (500 mg, 6.94 mmol) cyclopropanation was executed as described in4.7.6.2. The product was purified via flash chromatography (75 g silica gel, 12×4.5 cm, *n*-pentane/diethylether4/1, fraction size: 25 mL).

yield: 175 mg (28 %), colourlessliquid, $C_5H_{10}O$, [86.13 g/mol]

R_f= 0.33 (cyclohexane/ethylacetate 4/1) (CAM)

GC-FID (KH_80_30_280): t_R= 2.0 min

^{1}H-NMR (300.36 MHz, $CDCl_3$): δ= 3.48–3.37 (p, $^3J_{HH}$= 11.1 Hz, 1H, H-2), 1.53 (s, 1H, H-1), 1.04 (d, $^3J_{HH}$= 5.9 Hz, 3H, H-6), 0.90–0.71 (m, 1H, H-3), 0.71–0.53 (m, 1H, H-5), 0.45-0.05 (m, 2H, H-4) ppm.

^{13}C-NMR (75.53 MHz, $CDCl_3$): δ= 67.3 (C-2), 22.4 (C-3), 18.6 (C-6), 11.3 (C-5), 11.2 (C-4) ppm.

The recorded spectra are in accordance with the reported in literature.[28]

4.7.6.11 ((1*S*,2*R*)-2-Methylcyclopropyl)methanol((*S*,*R*)-53)

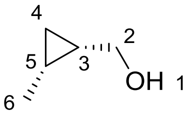

(*S*,*R*)-**53**

In a flame dried 80 mL Schlenk-flask, 1,2-DME (2.0 mL, 19.0 mmol) was dissolved in 18 mL dry CH_2Cl_2.In the meanwhile, in a second flame dried 250 mL three-neck round-bottom flask equipped with nitrogen inlet and a bubble counter,(*Z*)-but-2-en-1-ol(*cis*-**52**) (540 mg, 7.50 mmol) and (4*S*,5*S*)-2-butyl-N^4,N^4,N^5,N^5-tetramethyl-1,3,2-dioxaborolane-4,5-dicarboxamide((4*S*,5*S*)-**54**) (2.2 g, 8.14 mmol) were dissolved in 37 mL dry CH_2Cl_2. Both reaction mixtures were cooled to -10 °C using a NaCl/ice-water bath. To the solution of 1,2-DME and CH_2Cl_2, Et_2Zn (1.0 M solution in hexanes, 19.0 mL, 19.0 mmol) was added dropwise via syringe forming a cloudysuspension. Then CH_2I_2 (3.0 mL, 37.0 mmol) was added dropwise and after the reaction mixture was stirred for 15 min at -10 °C, the $Zn(CH_2I)_2$*DME solution was added to the 250 mL three-neck round-bottom flask containing the allylic alcohol via cannula. The reaction solution was stirred at 0 °C for 2 h and the reaction was quenched by the addition of sat.aq. NH_4Cl-solution (50 mL).The aqueous phase was extracted with CH_2Cl_2 (3x30 mL), dried over Na_2SO_4, and the solvent was evaporated under reduced pressure. The product was purified via flash chromatography (200 g silica gel, 17×5.5 cm, *n*-pentane/diethylether4/1, fraction size: 75 mL).

yield: 57 mg (9 %), colourlessliquid, $C_5H_{10}O$, [86.13 g/mol]

R_f= 0.33 (cyclohexane/ethylacetate 4/1) (CAM)

GC-FID (KH_80_30_280): t_R= 2.1 min

^{1}H-NMR (300.36 MHz, $CDCl_3$): δ= 3.89–3.63 (m, 1H, H-2), 3.63–3.35 (m, 1H, H-2), 1.36 (s, 1H, H-1), 1.07 (dd, $^3J_{HH}$= 14.2, 6.4 Hz, 4H, H-6, H-5), 1.00–0.85 (m, 1H, H-3), 0.81–0.46 (m, 1H, H-4), -0.07 (dd, , $^3J_{HH}$= 10.0, 5.0 Hz, 1H, H-4) ppm.

^{13}C-NMR (75.53 MHz, $CDCl_3$): δ= 63.3(C-2), 18.2 (C-5), 13.2 (C-6), 10.7 (C-4), 9.8 (C-3) ppm.

$[\alpha]^{20}_D$= +37.4 (c 0.9, CH_2Cl_2); lit.: $[\alpha]^{23}_D$= +43.4 (c 0.9, CH_2Cl_2).[Ref. 27]

The recorded spectra are in accordance with the reported in literature.[27]

Racemic sample: GC-FID(CP-Chirasil-Dex CB, KH_40_60_155)

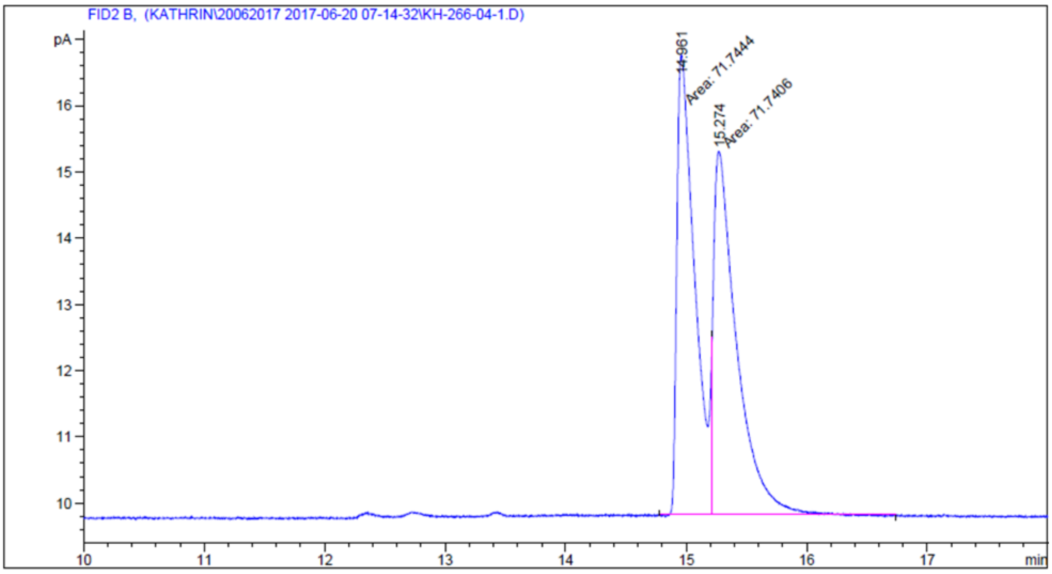

Peak #	RetTime [min]	Type	Width [min]	Area [pA*s]	Height [pA]	Area %
1	14.961	MF	0.1722	71.74436	6.94476	50.00133
2	15.274	FM	0.2181	71.74055	5.48316	49.99867

Enantioenriched sample: GC-FID (CP-Chirasil-Dex CB, KH_40_60_155), 73 % ee

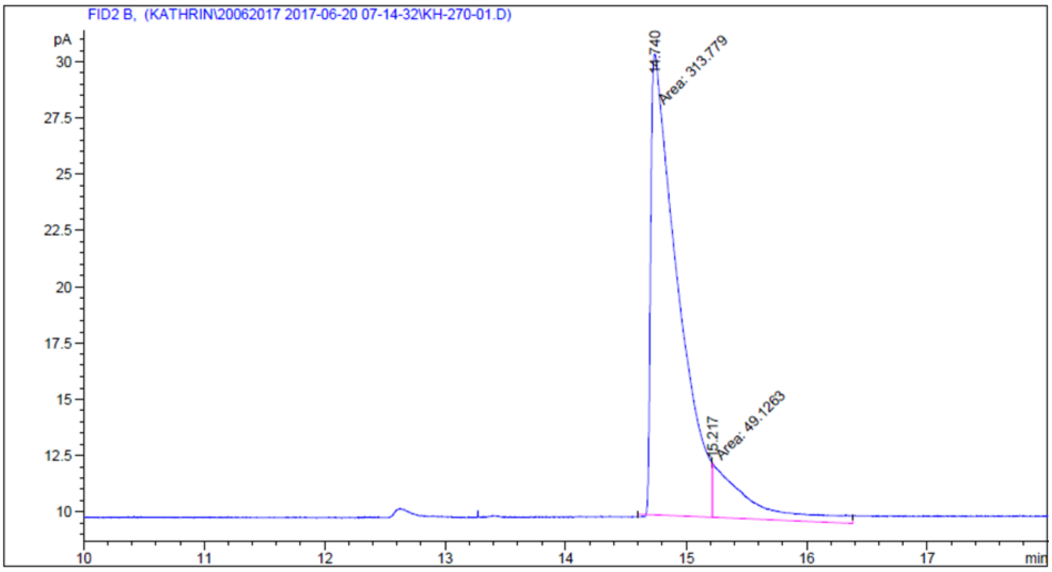

Peak #	RetTime [min]	Type	Width [min]	Area [pA*s]	Height [pA]	Area %
1	14.740	MF	0.2555	313.77863	20.46783	86.46304
2	15.217	FM	0.3373	49.12629	2.42716	13.53696

4.7.6.12 ((1*S*,2*S*)-2-Methylcyclopropyl)methanol ((*S*,*S*)-53)

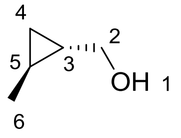

(*S*,*S*)-**53**

In a flame dried 80 mL Schlenk-flask, 1,2-DME (2.0 mL, 19.0 mmol) was dissolved in 18 mL dry CH_2Cl_2.In the meanwhile, in a second flame dried 250 mL three-neck round-bottom flask equipped with nitrogen inlet and a bubble counter,(*E*)-but-2-en-1-ol (*trans*-**52**) (540 mg, 7.50 mmol) and (4*R*,5*R*)-2-butyl-N^4,N^4,N^5,N^5-tetramethyl-1,3,2-dioxaborolane-4,5-dicarboxamide ((4*R*,5*R*)-**54**) (2.2 g, 8.14 mmol) were dissolved in 37 mL dry CH_2Cl_2. Both reaction mixtures were cooled to -10 °C using a NaCl/ice-water bath. To the solution of 1,2-DME and CH_2Cl_2, Et_2Zn (1.0 M solution in hexanes, 19.0 mL, 19.0 mmol) was added dropwise via syringe forming a cloudysuspension. Then CH_2I_2 (3.0 mL, 37.0 mmol) was added dropwise and after the reaction mixture was stirred for 15 min at -10 °C, the $Zn(CH_2I)_2$*DME solution was added to the 250 mL three-neck round-bottom flask containing the allylic alcohol via cannula. The reaction solution was stirred at 0 °C for 2 h and the reaction was quenched by the addition of saturated aqueous NH_4Cl-solution (50 mL).The aqueous phase was extracted with CH_2Cl_2 (3x30 mL), dried over Na_2SO_4, and the solvent was evaporated under reduced pressure. The product was purified via flash chromatography (150 g silica gel, 15×4.5 cm, *n*-pentane/diethylether4/1, fraction size: 50 mL).

yield:247 mg (38 %), colourlessliquid, $C_5H_{10}O$, [86.13 g/mol]

R_f= 0.33 (cyclohexane/ethylacetate 4/1) (CAM)

GC-FID (KH_80_30_280): t_R= 2.0 min

^{1}H-NMR (300.36 MHz, $CDCl_3$): δ= 3.48–3.37 (p, $^3J_{HH}$= 11.1 Hz, 1H, H-2), 1.53 (s, 1H, H-1), 1.04 (d, $^3J_{HH}$= 5.9 Hz, 3H, H-6), 0.90–0.71 (m, 1H, H-3), 0.71–0.53 (m, 1H, H-5), 0.45-0.05 (m, 2H, H-4) ppm.

^{13}C-NMR (75.53 MHz, $CDCl_3$): δ= 67.3 (C-2), 22.4 (C-3), 18.6 (C-6), 11.3 (C-5), 11.2 (C-4) ppm.

$[\alpha]^{20}_D$ = +46.5 (c 0.13, CH_2Cl_2); lit.: value not available.[29]

The recorded spectra are in accordance with the reported in literature.[29]

Racemic sample: GC-FID (CP-Chirasil-Dex CB, KH_40_60_155)

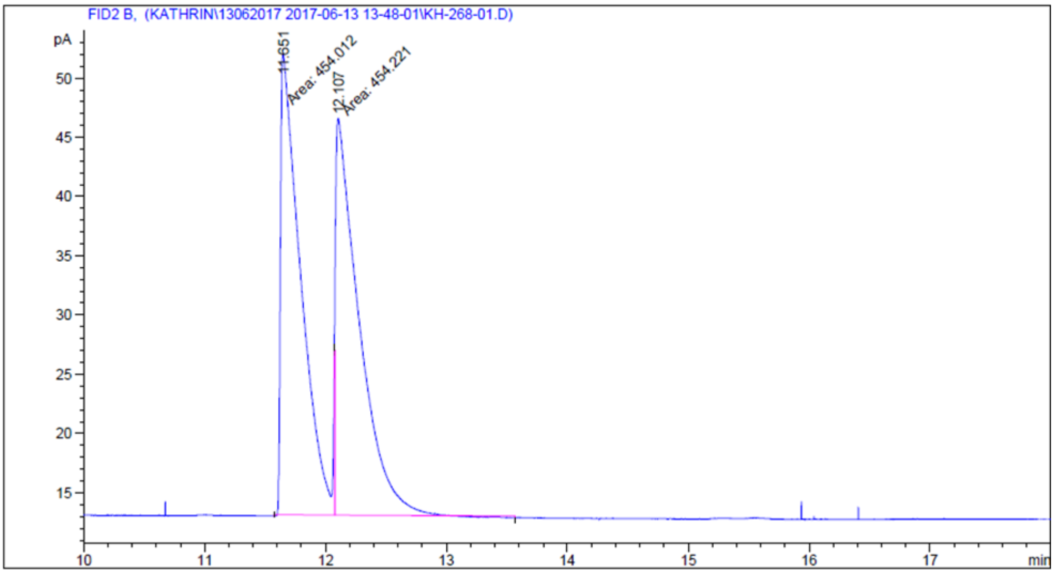

Peak #	RetTime [min]	Type	Width [min]	Area [pA*s]	Height [pA]	Area %
1	11.651	MF	0.1946	454.01233	38.88210	49.98851
2	12.107	FM	0.2262	454.22104	33.47151	50.01149

Enantioenriched sample: GC-FID (CP-Chirasil-Dex CB, KH_40_60_155),86 % ee

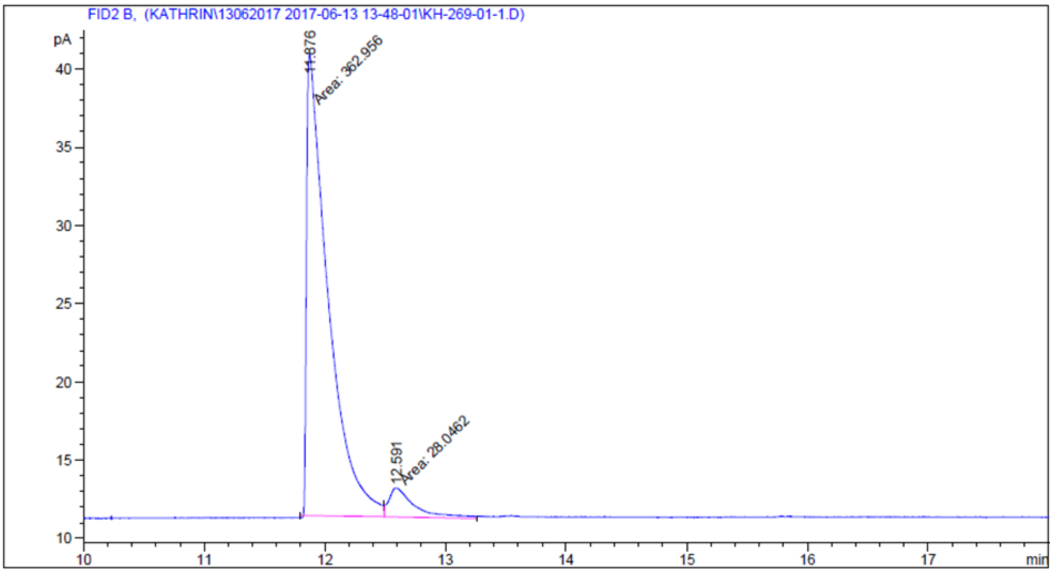

Peak #	RetTime [min]	Type	Width [min]	Area [pA*s]	Height [pA]	Area %
1	11.876	MF	0.2051	362.95566	29.49018	92.82710
2	12.591	FM	0.2505	28.04618	1.86611	7.17290

4.7.6.13 (4*S*,5*S*)-2-Butyl-*N*[4],*N*[4],*N*[5],*N*[5]-tetramethyl-1,3,2-dioxaborolane-4,5-dicarboxamide ((4*S*,5*S*)-54)

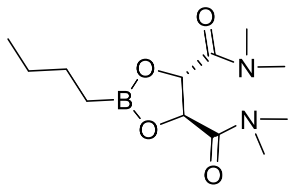

(4*S*,5*S*)-54

In a 50 mL round–bottom flask equipped with a Dean-Stark apparatus (2*S*,3*S*)-2,3-dihydroxy-*N*[1],*N*[1],*N*[4],*N*[4]-tetramethylsuccinamide (1.30 g, 6.4 mmol) and *n*-butylboronic acid (615 mg, 6.0 mmol) were heated under reflux in toluene (60 mL) until no more water distilled (8 h). The mixture was cooled to 22 °C and the solids were collected by filtration and washed with toluene (3×10 mL). The combined filtrates were concentrated under reduced pressure and the colourless oil was dried under oil pump vacuum for 6 h.

yield:1.66 g (97 %), colourlessliquid, $C_{12}H_{23}BN_2O_4$, [270.13 g/mol]

^{1}H-NMR (300.36 MHz, $CDCl_3$): δ= 5.40 (s, 2H, CHO), 3.03 (s, 6H, $N(CH_3)_2$), 2.86 (s, 6H, $N(CH_3)_2$), 1.29 (m, 4H, CH_2-CH_2), 0.81 (m, 5H, CH_2, CH_3) ppm.

^{13}C-NMR (75.53 MHz, $CDCl_3$): δ= 167.9 (C_q, C=O), 75.1 (CHO), 36.4 (CH_3), 35.3 (CH_3), 25.6 (CH_2), 24.5 (CH_2), 13.7 (CH_3), 9.6 (CH_2) ppm.

$[\alpha]^{20}_D$ =+90.4 (c 0.18, $CHCl_3$); lit.: value not available.[27]

The recorded spectra are in accordance with the reported in literature.[27]

4.7.6.14 (4*R*,5*R*)-2-Butyl-*N*[4],*N*[4],*N*[5],*N*[5]-tetramethyl-1,3,2-dioxaborolane-4,5-dicarboxamide ((4*R*,5*R*)-54)

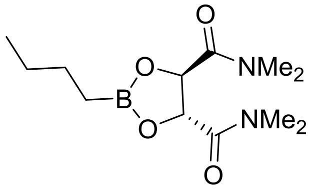

(4*R*,5*R*)-54

In a 50 mL round–bottom flask equipped with a Dean-Stark apparatus (2*R*,3*R*)-2,3-dihydroxy-*N*[1],*N*[1],*N*[4],*N*[4]-tetramethylsuccinamide (2.14 g, 10.5 mmol) and *n*-butylboronic acid (1.02 g,

10.0 mmol) were heated under reflux in toluene (60 mL) until no more water distilled (8 h). The mixture was cooled to 22 °C and the solids were collected by filtration and washed with toluene (3×10 mL). The combined filtrates were concentrated under reduced pressure and the colourless oil was dried under oil pump vacuum for 6 h.

yield: 2.53 g (94 %), colourlessliquid, $C_{12}H_{23}BN_2O_4$, [270.13 g/mol]

^{1}H-NMR (300.36 MHz, $CDCl_3$): δ= 5.40 (s, 2H, CHO), 3.04 (s, 6H, $N(CH_3)_2$), 2.86 (s, 6H, $N(CH_3)_2$), 1.29 (m, 4H, CH_2-CH_2), 0.82 (m, 5H, CH_2, CH_3) ppm.

^{13}C-NMR (75.53 MHz, $CDCl_3$): δ= 167.9 (C_q, C=O), 75.1 (CHO), 36.4 (CH_3), 35.3 (CH_3), 25.6 (CH_2), 24.5 (CH_2), 13.7 (CH_3), 9.6 (CH_2) ppm.

$[\alpha]^{20}_D$ =-82.1 (c 0.17, $CHCl_3$); lit.: $[\alpha]_D$= -114.3 (c 1.71, $CHCl_3$).[30]

The recorded spectra are in accordance with the reported in literature.[30]

4.7.7 Synthesis of aldehydes for reductive cyclization reactions

4.7.7.1 General procedure for reduction with DIBAL-H for unsaturated esters 1d-Br, 16, 25, 28, 29, 33

These experiments were executed under the complete exclusion of light. In a flame dried 250 mL two-neck round-bottom flask with nitrogen inlet a 0.30 M (1.0 eq) solution of the corresponding ester in dry CH_2Cl_2 was cooled to -78 °C using an acetone/dry ice bath. 2.1 eq DIBAL-H (1.0 M solution in CH_2Cl_2) were added dropwise to the reaction mixture in a N_2-counterstream. After 15 min full conversion was indicated by TLC. The reaction mixture was quenched by the addition of excess 1 M HClat -78 °C and warmed to 22 °C. After stirring for 30 min the colorless layers were separated and the aqueous phase was extracted with CH_2Cl_2 (2x50 mL). The combined organic layers were washed with brine (1x100 mL) and dried over Na_2SO_4. The solution of the allylic alcohol was immediately used in the next reaction without further purification.

4.7.7.2 General oxidation procedure with $MnO_2$to substrates 1a-Br, 1a-Cl, 2a-Br, 2a-I, 3a-Br, 3a-I

These experiments were executed under the complete exclusion of light. 1.0 g anhydrous 4 Å MS was transferred into a flame dried 250 mL two-neck round-bottom flask with nitrogen inlet. A 0.10 M (1.0 eq) solution of the corresponding allylic alcohol in CH_2Cl_2 and activated MnO_2 (5.0 eq) were added in a N_2-counterstream. The black reaction mixture was stirred for 8 h. After full conversion of the starting material was indicated by TLC and GC-FID, MnO_2 was removed via filtration through a pad of silica which was rinsed with CH_2Cl_2. The filtrate was collected and the solvent was evaporated under reduced pressure taking care that the bath temperature did not exceed 35 °C. Purification procedure and analytical data are stated for each substrate.

4.7.7.3 (*E*)-4-Bromobut-2-enal (1a-Br)

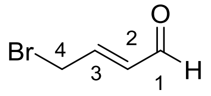

1a-Br

Starting fromethyl-4-bromocrotonate (**1d-Br**) (2.0 mL, 2.8 g, 14.5 mmol) reduction with DIBAL-H and oxidation with activated MnO_2 was executed as described in 4.7.7.1 and 4.7.7.2.

yield: 700 mg (33 %), brown liquid, C_4H_5BrO, [148.99 g/mol]

R_f= 0.68 (cyclohexane/ethylacetate 1/1) ($KMnO_4$)

GC-FID (KH_80_30_280): t_R= 3.6 min

^{1}H-NMR (300.36 MHz, $CDCl_3$): δ= 9.63 (dd, $^3J_{HH}$= 15.7, 7.5 Hz, 1H, H-1), 6.88 (dt, $^3J_{HH}$= 14.6, 7.3 Hz, 1H, H-3), 6.24 (dt, $^3J_{HH}$= 18.0, 9.0 Hz, 1H, H-2), 4.11 (dd, $^3J_{HH}$= 7.3, 1.0 Hz, 2H, H-4) ppm.

^{13}C-NMR (75.53 MHz, $CDCl_3$): δ= 192.8 (C-1), 149.5 (C-3), 134.3 (C-2), 28.7 (C-4) ppm. NMR spectra are in accordance with previously reported ones.[31]

4.7.7.4 (*E*)-4-Chlorobut-2-enal (1a-Cl)

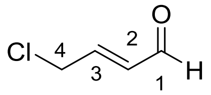

1a-Cl

Starting from ethyl-4-chlorocrotonate (**16**) (750 mg, 5.05 mmol) reduction with DIBAL-H and oxidation with activated MnO_2 was executed as described described in 4.7.7.1 and 4.7.7.2

yield: 70 mg (13 %), orange-brownish liquid, C_4H_5ClO, [104.53 g/mol]

R_f= 0.64 (cyclohexane/ethylacetate 1/1) ($KMnO_4$)

GC-FID (KH_80_30_280): t_R= 2.9 min

^{1}H-NMR (300.36 MHz, $CDCl_3$): δ= 9.62 (d, $^3J_{HH}$= 7.6 Hz, 1H, H-1), 6.85 (dt, $^3J_{HH}$= 15.4, 5.9 Hz, 1H, H-3), 6.35 (dd, $^3J_{HH}$= 15.4, 7.6 Hz, 1H, H-2), 4.27-4.20 (m, 2H, H-4) ppm.

^{13}C-NMR (75.53 MHz, $CDCl_3$): δ= 192.7 (C-1), 149.6 (C-3), 134.0 (C-2), 42.4 (C-4) ppm.

NMR spectra are in accordance with previously reported ones.[32]

4.7.7.5 (*E*)-4-Chloro-3-methylbut-2-enal (5)

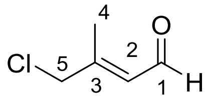

5

Starting from ethyl (*E*)-4-chloro-3-methylbut-2-enoate (**28**) (700 mg, 4.30 mmol) reduction with DIBAL-H and oxidation with activated MnO_2 was executed as described in 4.7.7.1 and 4.7.7.2.

yield: 130 mg (26 %), yellowish liquid, C_5H_7ClO, [118.02 g/mol]

R_f= 0.23 (cyclohexane/ethylacetate 5/1) ($KMnO_4$)

GC-FID (KH_80_30_280): t_R= 3.6 min

^{1}H-NMR (300.36 MHz, $CDCl_3$): δ= 10.0 (d, $^3J_{HH}$= 7.6 Hz, 1H, H-1), 6.10 (d, $^3J_{HH}$= 6.9 Hz, 1H, H-2), 4.10 (s, 2H, H-5), 2.26 (s, 3H, H-4) ppm.

^{13}C-NMR (75.53 MHz, $CDCl_3$): δ= 191.6 (C-1), 155.3 (C-3), 128.7 (C-2), 49.4 (C-5), 15.7 (C-4) ppm.
HRMS (EI): Calcd. (m/z) for C_5H_7ClO [M^+]:118.0185; found: 118.0185.

4.7.7.6 (*E*)-4-Bromo-3-methylbut-2-enal (7)

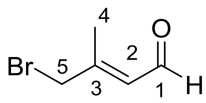

7

Starting from ethyl (*E*)-4-bromo-3-methylbut-2-enoate (**29**) (1.0 g, 4.83 mmol) reduction with DIBAL-H and oxidation with activated MnO_2 was executed as described in 4.7.7.1 and 4.7.7.2.

yield: 270 mg (34 %), yellow and brownish liquid, C_5H_7BrO, [163.01 g/mol]

R_f= 0.45 (cyclohexane/ethylacetate 5/1) ($KMnO_4$)

GC-FID (KH_80_30_280): t_R= 3.6 min

^{1}H-NMR (300.36 MHz, $CDCl_3$): δ= 10.0 (d, $^{3}J_{HH}$= 7.6 Hz, 1H, H-1), 6.10 (d, $^{3}J_{HH}$= 7.4 Hz, 1H, H-2), 4.00 (s, 2H, H-5), 2.30 (s, 3H, H-4) ppm.

^{13}C-NMR (75.53 MHz, $CDCl_3$): δ= 191.1 (C-1), 155.5 (C-3), 129.1 (C-2), 37.3 (C-5), 16.0 (C-4) ppm.
NMR spectra are in accordance with previously reported ones.[33]

4.7.7.7 (*E*)-4-Chloro-3-phenylbut-2-enal (10)

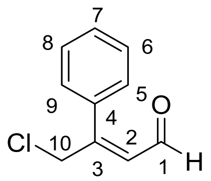

10

Starting from ethyl (*E*)-4-chloro-3-phenylbut-2-enoate (**33**) (395 mg, 1.76 mmol) reduction with DIBAL-H and oxidation with activated MnO_2 was executed as described in 4.7.7.1 and 4.7.7.2. The product was purified via flash chromatography (50 g silica gel, 10×1.5 cm, cyclohexane/ethylacetate 12/1, fraction size: 25 mL).

yield: 181 mg (57 %), yellowish liquid, $C_{10}H_9ClO$, [180.23 g/mol]

R_f= 0.53 (cyclohexane/ethylacetate 4/1) ($KMnO_4$)

GC-FID (KH_80_30_280): t_R= 6.1 min

^{1}H-NMR (300.36 MHz, DMSO-d_6): δ= 9.42 (d, $^{3}J_{HH}$= 7.9 Hz, 1H, H-1), 7.50 (s, 5H, H-5, H-6, H-7, H-8, H-9), 6.40 (d, $^{3}J_{HH}$= 7.9 Hz, 1H, H-2), 4.82 (s, 2H, H-10) ppm.

^{13}C-NMR (75.53 MHz, DMSO-d_6): δ= 192.5 (C-1), 158.7 (C-2), 134.3 (C-3), 129.7 (C-4), 129.2 (C-5, C-9), 128.9 (C-7), 128.5 (C-6, C-8), 47.7 (C-10) ppm.

HRMS (EI): Calcd. (m/z) for $C_{10}H_8O$ [M-HCl]:180.0342; found: 180.0347.

4.7.7.8 (*E*)-4-Chloro-2-methylbut-2-enal (4a-Cl)

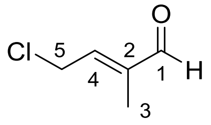

4a-Cl

Starting from ethyl (*E*)-4-chloro-2-methylbut-2-enoate(**25**) (1.0 g, 6.20 mmol) reduction with DIBAL-H and oxidation with activated MnO_2 was executed as described in 4.7.7.1 and 4.7.7.2.

yield: 328 mg (45 %), yellowish liquid, C_5H_7ClO, [118.02 g/mol]

R_f= 0.72 (cyclohexane/ethylacetate 3/1) ($KMnO_4$)

GC-FID (KH_80_30_280): t_R= 3.2 min

^{1}H-NMR (300.36 MHz, $CDCl_3$): δ= 9.48 (s, 1H, H-1), 6.55 (dd, $^3J_{HH}$= 7.4, 6.3 Hz, 1H, H-2), 4.30 (d, $^3J_{HH}$= 7.4 Hz, 2H, H-5), 1.82 (s, 3H, H-3) ppm.

^{13}C-NMR (75.53 MHz, $CDCl_3$): δ= 194.4 (C-1), 145.8 (C-4), 141.3 (C-2), 38.8 (C-5), 9.3 (C-3) ppm.
The recorded spectra are in accordance with the reported in literature.[34]

4.7.7.9 Ethyl (*E*)-5-bromopent-2-enoate (2a-Br)

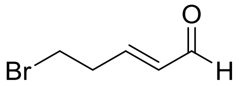

2a-Br

Starting from **18** (1.0 g, 6.13 mmol) reduction with DIBAL-H and oxidation with activated MnO_2 was executed as described in 4.7.7.1 and 4.7.7.2.

yield: 148 mg (15 %), yellowish liquid, C_5H_7BrO, [163.01 g/mol]

R_f= 0.72 (cyclohexane/ethylacetate 3/1) ($KMnO_4$)

GC-FID (KH_80_30_280): t_R= 4.3 min

Aldehyde **2a-Br** was notoriously unstable and defied our efforts for characterisation. It was directly engaged in the subsequent step, described previously by Sabbatani *et al.*[35]

4.7.7.10 Ethyl (*E*)-5-iodopent-2-enoate (2a-I)

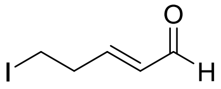

2a-I

Starting from **19** (1.5 g, 7.14 mmol) reduction with DIBAL-H and oxidation with activated MnO_2 was executed as described in 4.7.7.1 and 4.7.7.2.

yield: 173 mg (12 %), yellowish liquid, C_5H_7IO, [210.01 g/mol]

R_f= 0.72 (cyclohexane/ethylacetate 3/1) ($KMnO_4$)

GC-FID (KH_80_30_280): t_R= 4.8 min

Aldehyde **2a-I** was notoriously unstable and defied our efforts for characterisation. It was directly engaged in the subsequent step.

4.7.7.11 (*E/Z*)-6-Bromohex-2-enal (*E*/Z 3a-Br)

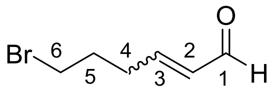

E/Z 3a-Br

In a flame dried 50 mL two-neck round-bottom flask with nitrogen inletGrubbs II Catalyst (28.6 mg, 0.03 mmol, 1 mol%) was suspended in 15 mL dry CH_2Cl_2. To the catalyst solution a mixture of 5-bromo-1-pentene (0.40 mL, 0.50 g, 3.38 mmol, 1.0 eq) and crotonaldehyde (1.40 mL, 1.19 g, 17.0 mmol, 5.0 eq) in 4 mL dry CH_2Cl_2 was added via cannula. The solution was stirred at 48 °C for 24 h until TLC indicated full conversion of the strating materials. The reaction mixture was cooled to 22 °C and the solution was filtered through a pad of silica and rinsed with CH_2Cl_2 (3×15 mL). The solvent was removed under reduced pressure and the product was used without further purification in the next step.

yield: 510 mg (86 %), slightly brown liquid, C_6H_9BrO, [177.04 g/mol].

4.7.7.12 (*E*)-6-Bromohex-2-enal (3a-Br)

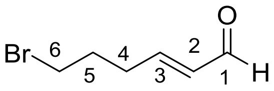

3a-Br

In a flame dried 25 mL Schlenk flask (*E/Z*)-6-bromohex-2-enal (***E*/Z3a-Br**) (128 mg, 0.72 mmol, 1.0 eq) was dissolved in 2.9 mL anhydrous acetonitrile (0.25 M solution). 4-(Dimethylamino)pyridine (0.8 mg, 7.23 µmol, 1 mol %) were added in a N_2 counter-stream and the solution was stirred for 8 h at RT. The solvent was removed under reduced pressure and the product was purified via flash chromatography (35 g silica gel, 10x3 cm, diethylether/*n*-pentane = 1:4, fraction size: 20 mL).

yield: 123 mg (96 %), slightly brown liquid, C_6H_9BrO, [177.04 g/mol].

Rf= 0.23 (diethylether/*n*-pentane =1:4) ($KMnO_4$)

GC-FID (KH_80_30_280): t_R= 4.9 min

^{1}H-NMR (300.36 MHz, $CDCl_3$):δ= 9.52 (d, $^{3}J_{HH}$= 7.8 Hz, 1H, H-1),6.82 (dt, $^{3}J_{HH}$= 15.6, 6.7Hz, 1H, H-3),6.16 (ddd, $^{3}J_{HH}$=15.7,7.8, 1.1 Hz, 1H, H-2), 3.43 (t, $^{3}J_{HH}$= 6.5 Hz, 2H, H-6), 2.52 (td, $^{3}J_{HH}$= 8.0, 1.3 Hz, 2H, H-4), 2.24-1.91 (m, 2H, H-5) ppm.

^{13}C-NMR (75.53 MHz, $CDCl_3$):δ= 193.8 (C-1), 156.1 (C-3), 133.9 (C-2), 32.4 (C-4), 31.1 (C-5), 30.7 (C-6) ppm.

The recorded spectra are in accordance with the reported in literature.[36]

4.7.7.13 5-Iodo-1-pentene (55)

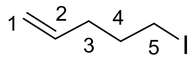

55

In a flame dried 25 mL Schlenk flask 5-bromo-1-pentene (0.8 mL, 1.00 g, 6.71 mmol, 1.0eq) was dissolved in 22 mL acetone. NaI (2.0 g, 13.3 mmol, 2.0 eq) was added in a N_2 counter-stream and the solution was stirred at 60 °C for 2 h as GC-FID indicated full conversion of the strating materials. The mixture was cooled to 22 °C and diluted with 100 mL of distilled water. The reaction solution was extracted with *n*-pentane (3×50 mL) and the combined organic layers were dried over Na_2SO_4. After filtration the solvent was removed by rotary evaporation and the product was used in the next reaction step without further purification.

yield: 1.23 mg (95 %), colourless liquid, C_5H_9I, [196.03 g/mol].

GC-FID (KH_80_30_280): t_R= 3.4 min

^{1}H-NMR (300.36 MHz, $CDCl_3$):δ= 5.75 (ddt, $^{3}J_{HH}$= 16.9, 10.2, 6.7 Hz, 1H, H-2),5.19-4.92 (m,2H, H-1),3.19 (t, $^{3}J_{HH}$=6.9 Hz, 2H, H-5), 2.17(q, $^{3}J_{HH}$= 7.0Hz, 2H, H-3), 1.91(p, $^{3}J_{HH}$= 6.9Hz, 2H, H-4) ppm.

^{13}C-NMR (75.53 MHz, $CDCl_3$):δ= 136.7 (C-2), 116.1 (C-1), 34.4 (C-3), 32.6 (C-4), 6.4 (C-5) ppm.

The recorded spectra are in accordance with the reported in literature.[37]

4.7.7.14 (*E*/*Z*)-6-Iodohex-2-enal (*E*/*Z* 3a-I)

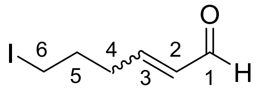

E/Z 3a-I

In a flame dried 100 mL two-neck round-bottom flask with nitrogen inlet, Grubbs II catalyst (53.3 mg,0.06 mmol, 1 mol %) was suspended in 30 mL dry CH_2Cl_2. To the catalyst solution a mixture of 5-iodo-1-pentene (**55**) (1.26 g, 6.43 mmol, 1.0 eq) and crotonaldehyde (2.58 mL, 2.20 g, 31.4 mmol, 5.0 eq) in 7 mL dry CH_2Cl_2 was added via cannula. The solution was stirred at 48 °C for 24 h until TLC indicated full conversion of the strating materials. The reaction mixture was cooled to 22 °C and the solution was filtered through a pad of silica and rinsed with CH_2Cl_2 (3×15 mL). The solvent was removed under reduced pressure and the product was used without further purification in the next step.

yield: 946 mg (66 %), slightly brown liquid, C_6H_9IO, [224.04 g/mol].

4.7.7.15 (*E*)-6-Iodohex-2-enal (3a-I)

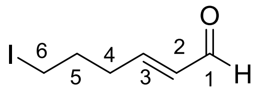

3a-I

In a flame dried 25 mL Schlenk flask (*E*/*Z*)-6-iodohex-2-enal (***E*/*Z*3a-I**) (946 mg, 4.22mmol, 1.0 eq) was dissolved in 17 mL anhydrous acetonitrile (0.25 M solution). 4-(Dimethylamino)pyridine (4.7 mg, 41.9 µmol, 1 mol %) were added in a N_2 counter-stream and the solution was stirred for 8 h at 22 °C. The solvent was removed under reduced pressure and the product was purified via flash chromatography (40 g silica gel, 12x3.5 cm, cyclohexane/ethylacetate = 9.5:1, fraction size: 20 mL).

yield: 940 mg (quant.), slightly brown liquid, C_6H_9IO, [224.04 g/mol].

Rf= 0.23 (cyclohexane/ethylacetate = 9.5:1) ($KMnO_4$)

GC-FID (KH_80_30_280): t_R= 5.4 min

^{1}H-NMR (300.36 MHz, $CDCl_3$):δ= 9.51 (d, $^3J_{HH}$= 7.8 Hz, 1H, H-1), 6.80 (dt, $^3J_{HH}$= 15.6, 6.7 Hz, 1H, H-3),6.16 (ddd, $^3J_{HH}$= 15.7,7.8, 1.1 Hz, 1H, H-2), 3.21(t, $^3J_{HH}$= 6.7Hz, 2H, H-6), 2.47(dt, $^3J_{HH}$= 7.5, 3.9Hz, 2H, H-4), 2.20-1.85 (m, 2H, H-5) ppm.

^{13}C-NMR (75.53 MHz, $CDCl_3$):δ= 193.8 (C-1), 155.8 (C-3), 133.9 (C-2), 32.4 (C-4), 31.3 (C-5), 5.1 (C-6) ppm.

HRMS (EI): Calcd. (m/z) for $C_7H_{10}O_2^+$ $[M]^+$: 223.9698, found 223.9707.

4.8 Biocatalysis Section

4.9 Analytical scale procedure for enzymatic reductive cyclization reactions

These experiments were executed under complete exclusion of light. An aliquot of enzyme (5 µM final concentration) was rehydrated in NaPi buffer (50 mM, 150 mMNaCl, pH 7.5) to a volume of 300 µL. The reaction was started by the addition of a stock solution (300 µL) containing the substrate (10 mM), NADH (15 mM), DMF (1 % final concentration), and 1,2-DME as an internal standard ($V_{Substrate}$=$V_{InternalStandard}$). The samples were incubated for 180 min at 25 °C and 300 rpm in an Eppendorf Thermomixer comfort. For work-up MTBE (300 µL) was added to each sample, which was then intensely vortexed and centrifuged for 10 min at 13.000 rpm. The organic layer was then separated and dried over $MgSO_4$. After centrifugation (10 min, 13.000 rpm), the organic layer was transferred into GC glass-vials and the samples were measured directly using GC-FID or HPLC. Conversions are stated as an average of three independent experiments.

Blank reactions were prepared for every screening run. One set of blanks was run without enzyme and the other set of blanks was run without NADH.

4.10 Determination of diastereomericexcess, enantiomeric excess and assignment of absolute configuration

A solution of $NaBH_4$ (10 wt% in dist. water, 50 µL per sample) was added tothe finished biocatalytic reactionmixtures in an Eppendorf vial and the samples were incubated for 30 min at 22 °C. The reaction mixtures were quenched by the addition of 1 M HCl (100 µL) and MTBE(300 µL) was added. The samples were intensely vortexedand after centrifugation (10 min, 13.000 rpm) the organic layer was separated and dried over $MgSO_4$. Once more, the samples were centrifuged (10 min, 13.000 rpm) and the organic layer was transferred into GC glass-vials and analysed as stated for each compound.

4.10.1 Diastereomericexcess and enantiomeric excess of (2-methylcyclopropyl)methanol

All samples were reduced as it is described in 4.10. The diastereomeric and the enantiomeric excess of (2-methylcyclopropylmethanol) (**53**) was determined as follows (Scheme S1):

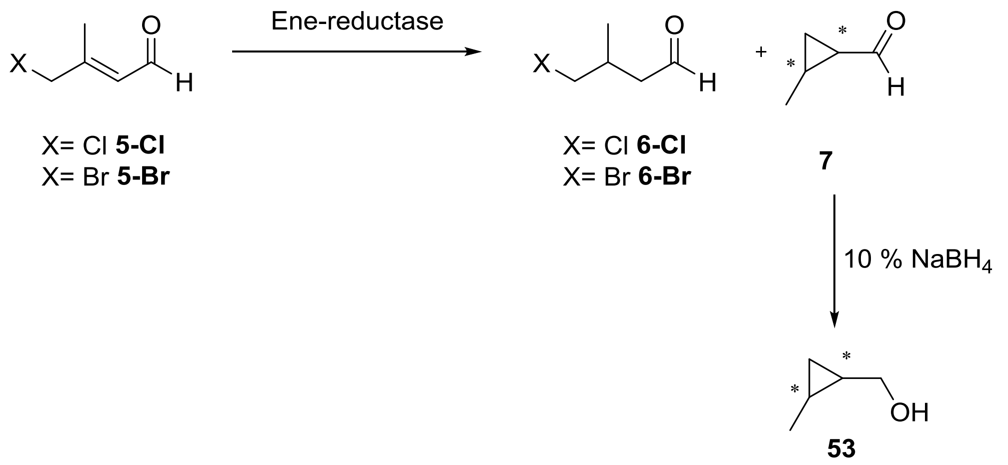

Scheme S1: Determination of diastereomeric excess and enantiomeric excess of (2-methylcyclopropyl)methanol (**53**).

Separation of diastereomers was achieved on achiral GC-FID using an Agilent Technologies J&W GC-column DB-17-01 and method KH_80_30_280.As shown in Figure S2 and Figure S3 the *cis*-diastereomer (*cis*-**53**) and the *trans*-diastereomer(*trans*-**53**) were identified by the injection of synthesized reference materials on achiral GC-FID. Figure S4 shows GC-FID chromatogram of the conversion of (*E*)-4-chloro-3-methylbut-2-enal (**5-Cl**) byYqjM Y169F after the sample had been reduced with $NaBH_4$.

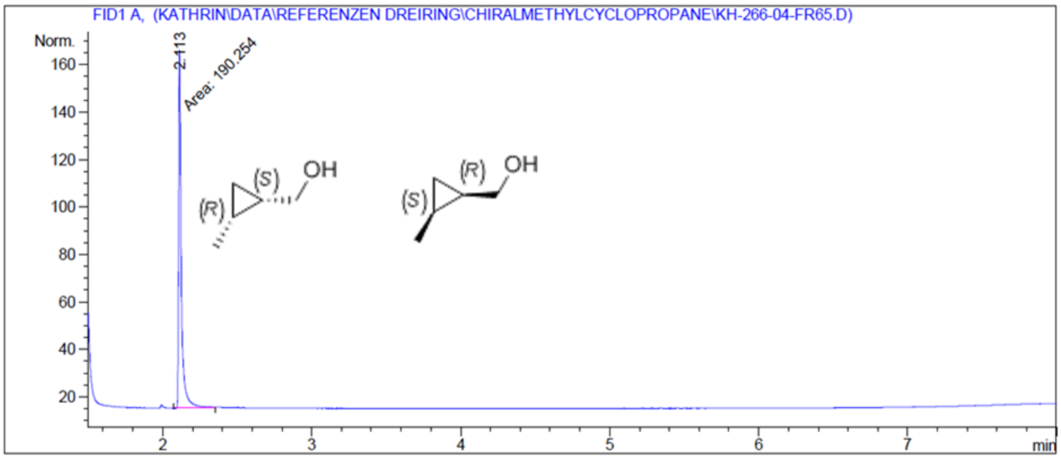

Figure S2: GC-FID chromatogram of(2-methylcyclopropyl)methanol *cis*-**53**.

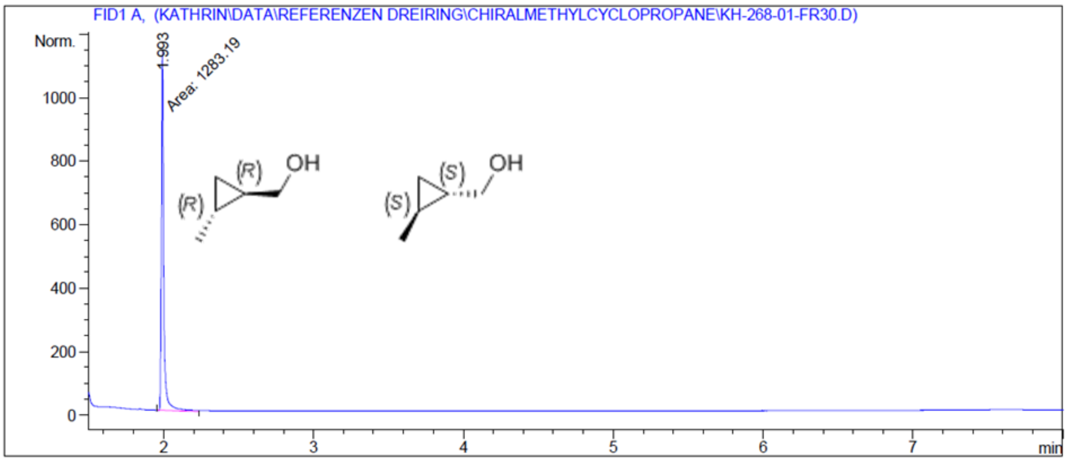

Figure S3: GC-FID chromatogram of(2-methylcyclopropyl)methanol *trans*-**53**.

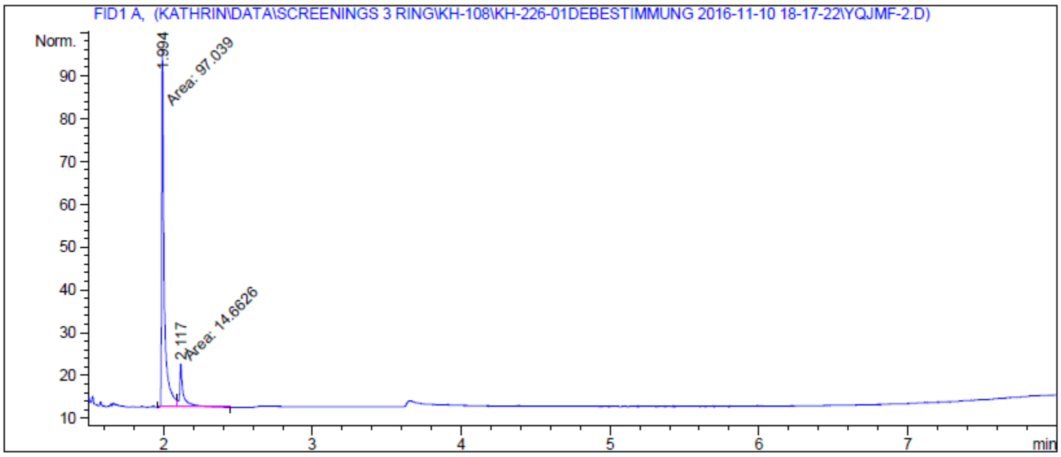

Figure S4: GC-FID chromatogram of the conversion of (*E*)-4-chloro-3-methylbut-2-enal (**5-Cl**) byYqjM Y169F after the sample had been reduced with $NaBH_4$.

For the determination of the absolute configuration and the enantiomeric excess samples were injected in chiral GC-FID. Separation of enantiomers was achieved on chiral GC-FID using an Agilent CP-Chirasil-Dex CB column and method KH_40_60_155. Synthesized reference material was injected to perform the determination of the absolute configuration (chromatograms were already shown in4.7.6.11 and 4.7.6.12). Figure S5 shows the conversion of (*E*)-4-chloro-3-methylbut-2-enal (**5-Cl**) by OPR3 WT after reduction with $NaBH_4$.

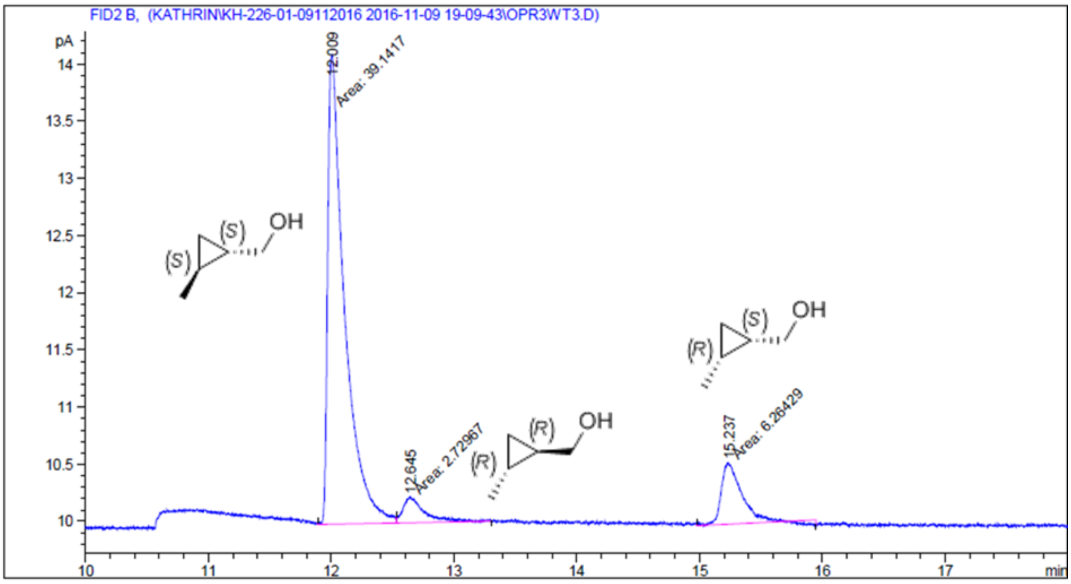

Figure S5: GC-FID chromatogram of the conversion of (*E*)-4-chloro-3-methylbut-2-enal (**5-Cl**) by OPR3 WT after reduction with $NaBH_4$.

4.10.2 Diastereomericexcess and enantiomeric excess of (2-phenylcyclopropyl)methanol

The diastereomeric excess of 2-phenylcyclopropane-1-carbaldehyde (**114**) and the enantiomeric excess of (2-phenylcyclopropylmethanol) (**115**) was determined as follows (Scheme S2):

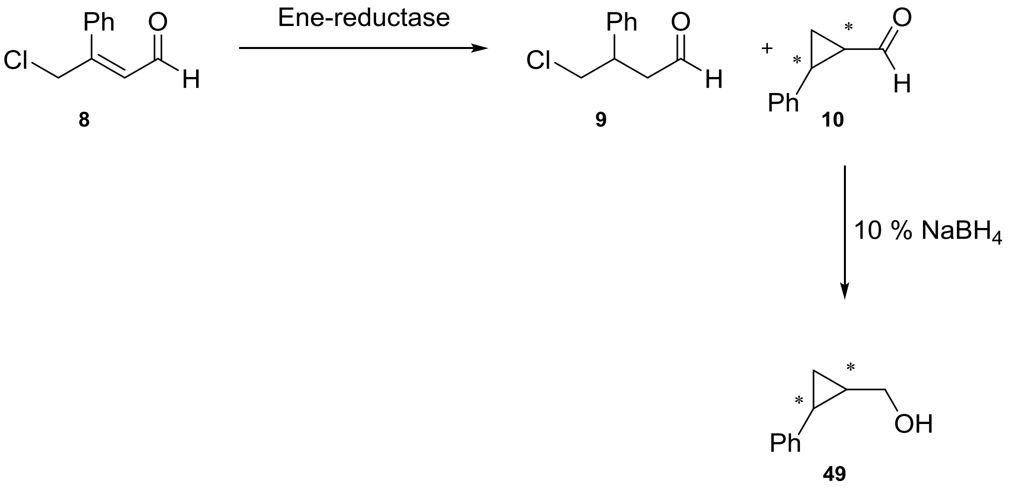

Scheme S2: Determination of diastereomeric excess of 2-phenylcyclopropane-1-carbaldehyde (**10**)and enantiomeric excess of (2-phenylcyclopropyl)methanol (**49**).

Separation of diastereomers was achieved on achiral GC-FID using an Agilent Technologies J&W GC-column DB-17-01 and method KH_80_30_280. As shown in Figure S6and Figure S7the *cis*-diastereomer(*cis*-**10**) and the *trans*-diastereomer(*trans*-**10**) were identified by the injection of synthesized reference materials on achiral GC-FID.Figure S8shows GC-FID chromatogram of theconversion of (*E*)-4-chloro-3-phenylbut-2-enal (**8**) by OPR3 Y190F.

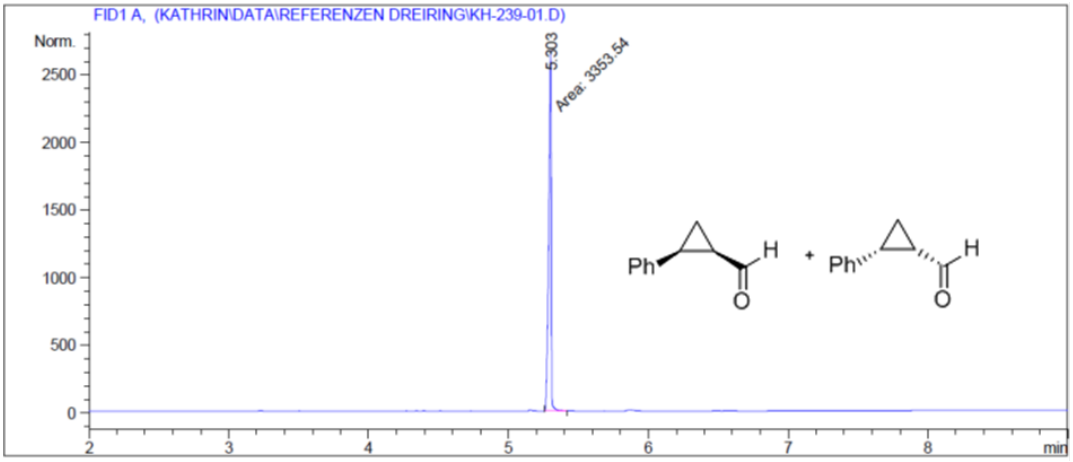

Figure S6:GC-FID chromatogram of 2-phenylcyclopropane-1-carbaldehyde (*cis*-**10**).

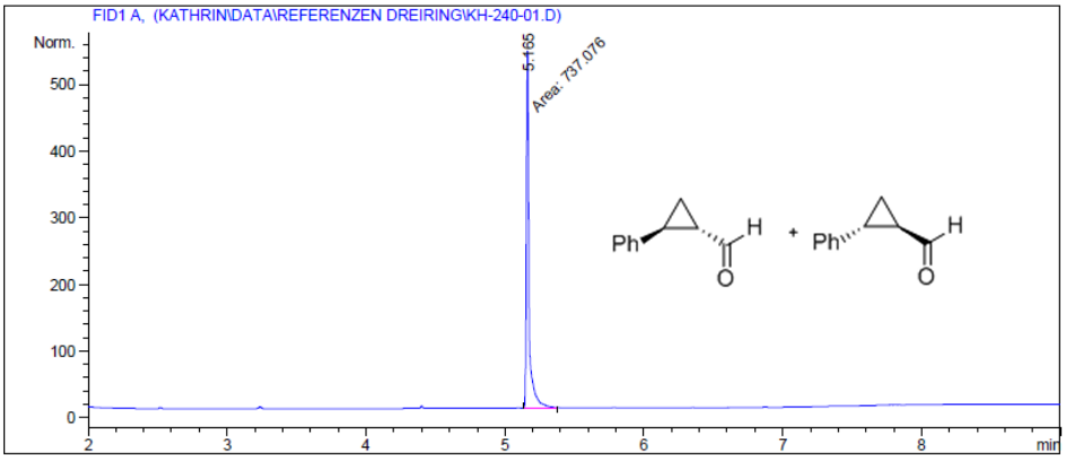

Figure S7:GC-FID chromatogram of 2-phenylcyclopropane-1-carbaldehyde (*trans*-**10**).

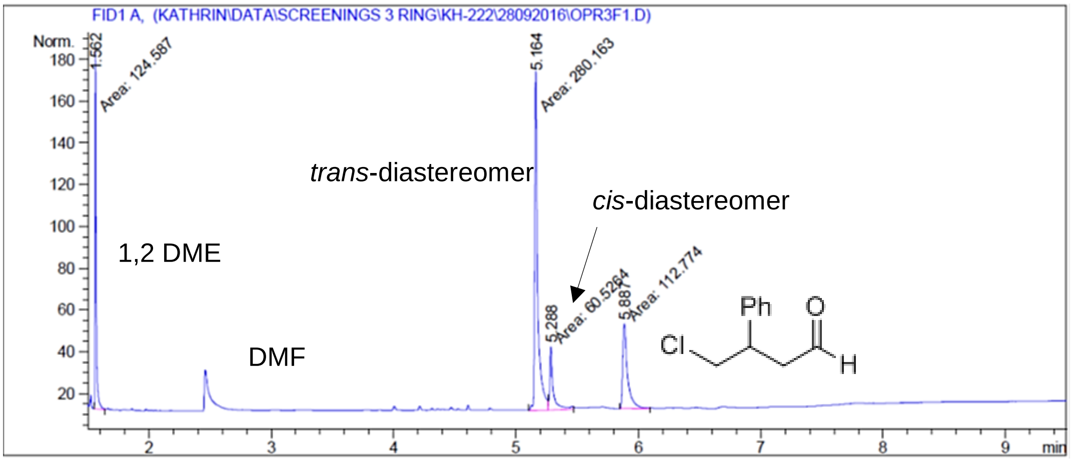

Figure S8: GC-FID chromatogram of the conversion of (*E*)-4-chloro-3-phenylbut-2-enal (**8**) by OPR3 Y190F.

For the determination of the absolute configuration and the enantiomeric excess,samples were reduced as it is described in 4.10.Separation of enantiomers of *trans*-(2-phenylcyclopropyl)methanol(*trans*-**49**) was achieved as follows (Figure S9): Daicel Chiralcel OD-H, *n*-hexane: iPrOH 9:1, flow= 0.5 mL min^{-1}. Elution order of enantiomers was previously described in literature.[38]

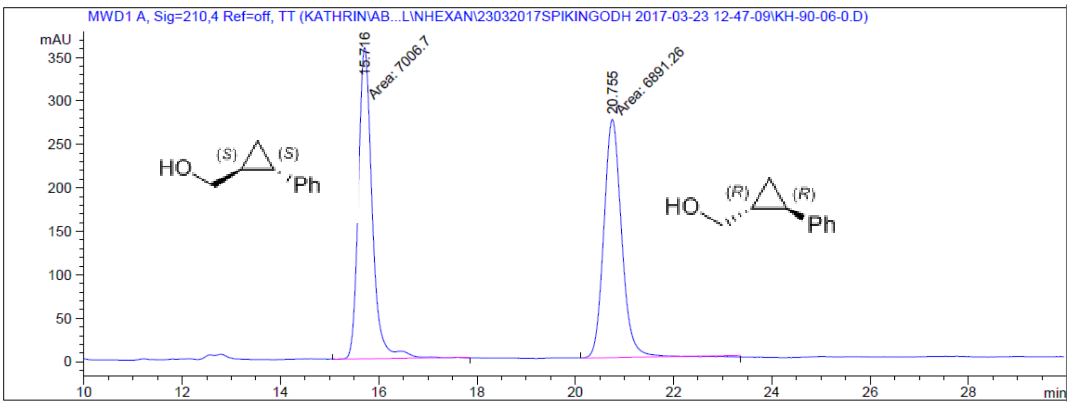

Peak #	RetTime [min]	Type	Width [min]	Area [mAU*s]	Height [mAU]	Area %
1	15.716	MM	0.3232	7006.69678	361.29327	50.4153
2	20.755	MM	0.4169	6891.25684	275.51080	49.5847

Figure S9:HPLC chromatogram of racemic sample of *trans*-(2-phenylcyclopropyl)methanol (*trans*-**49**).

Figure illustrates a HPLC chromatogram of the conversion of (*E*)-4-chloro-3-phenylbut-2-enal (**10**) by OPR3 Y190F using the above mentioned column and method. For the determination of the enantiomeric excess the sample was spiked with 0.2 µL of racemic *trans*-(2-phenylcyclopropyl)methanol(*trans*-**49**) (see Figure).

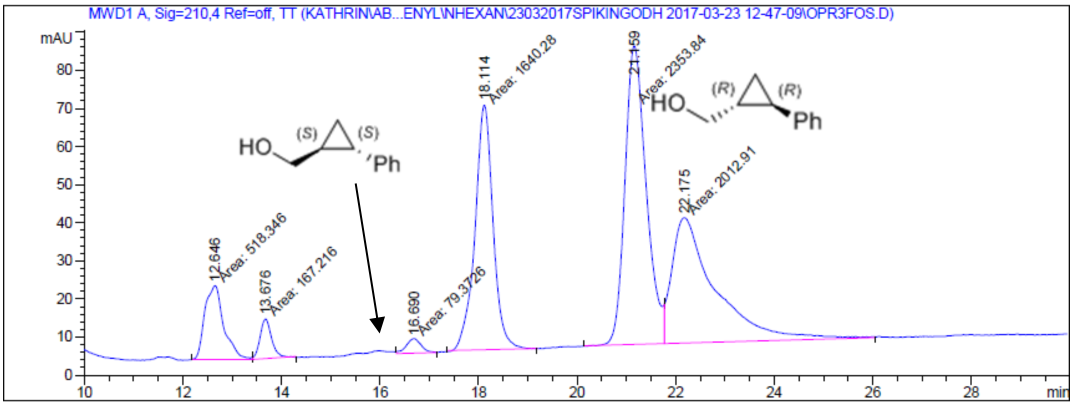

Figure S10: HPLC chromatogram of sample of OPR3 Y190F.

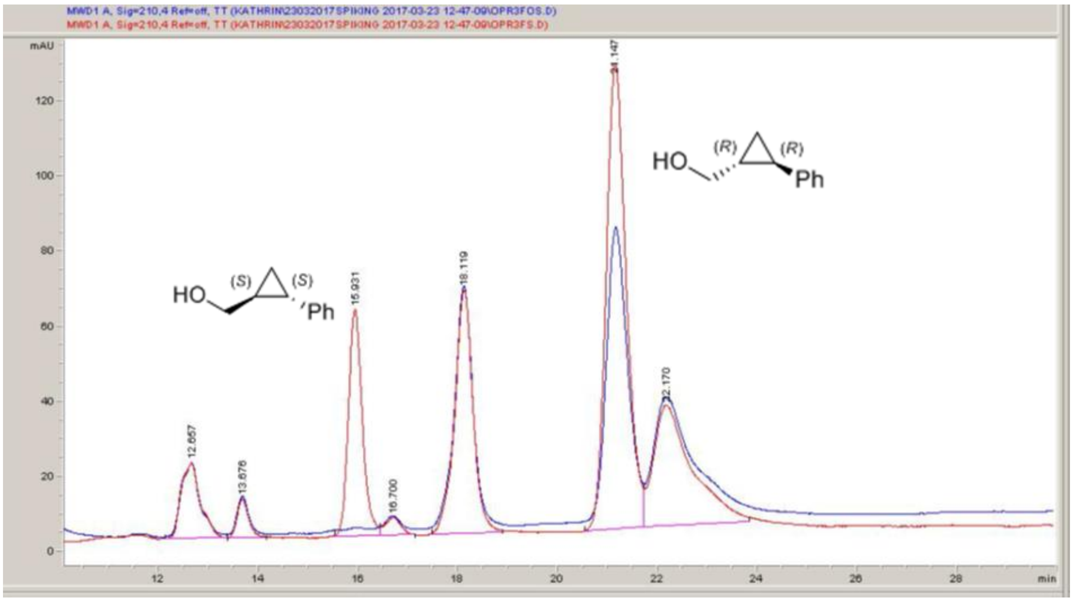

Figure S11: HPLC chromatogram of sample of OPR3 Y190F spiked with 0.2 µL of racemic *trans*-(2-phenylcyclopropyl)methanol (*trans*-**49**).

Separation of enantiomers of *cis*-(2-phenylcyclopropyl)methanol was achieved as follows:Daicel Chiralcel OJ-H, *n*-hexane: iPrOH 95:5, flow= 0.5 mL min^{-1}. Elution order of enantiomers was previously described in literature.[38]

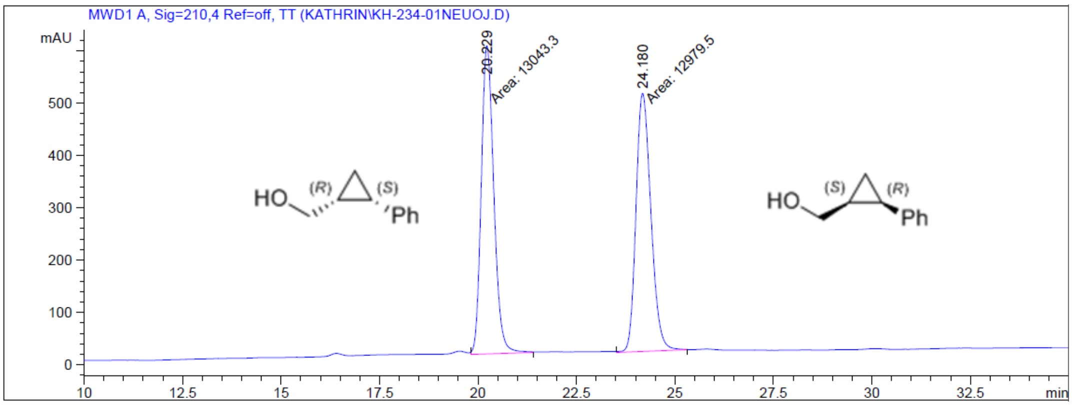

Peak #	RetTime [min]	Type	Width [min]	Area [mAU*s]	Height [mAU]	Area %
1	20.229	MM	0.3682	1.30433e4	590.42200	50.1226
2	24.180	MM	0.4366	1.29795e4	495.41827	49.8774

Figure S12: HPLC chromatogram of racemic sample of *cis*-(2-phenylcyclopropyl)methanol (*cis*-**49**).

Figure S13 illustrates a HPLC chromatogram of the conversion of (*E*)-4-chloro-3-phenylbut-2-enal (**10**) by OPR3 Y190F using the above mentioned column and method. For the determination of the enantiomeric excess the sample was spiked with 0.2 µL of racemic *cis*-(2-phenylcyclopropyl)methanol(*trans*-**49**) (see Figure S14).

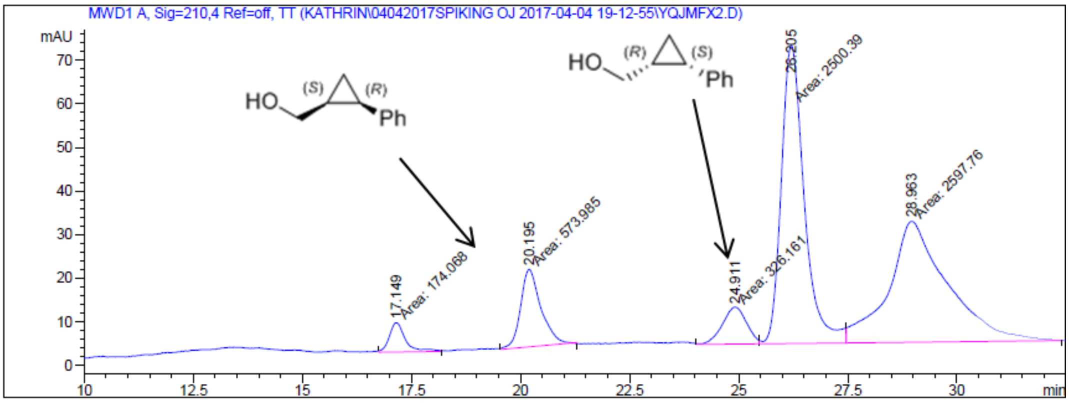

Figure S13: HPLC chromatogram of sample of YqjM Y169F.

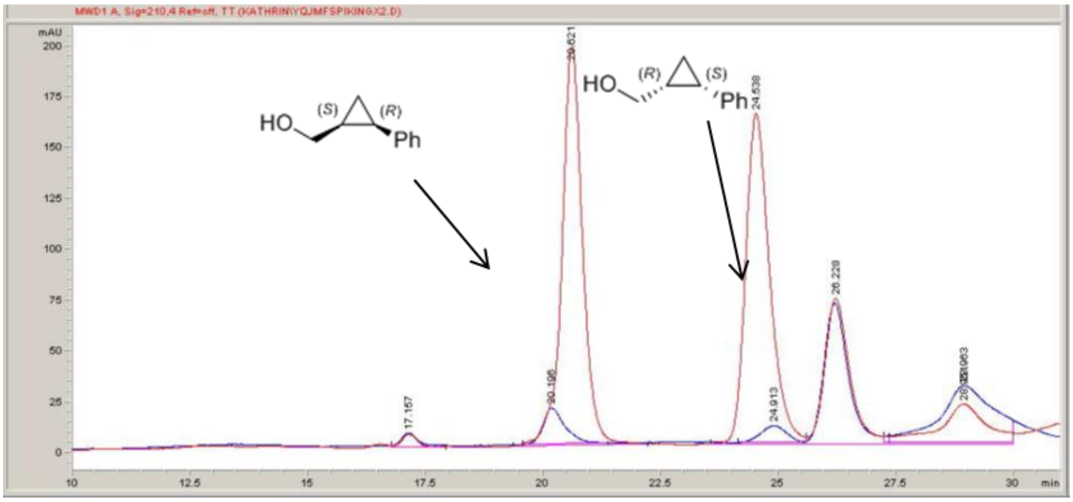

Figure S14: HPLC chromatogram of sample of YqjM Y169F spiked with 0.2 µL of racemic *cis*-(2-phenylcyclopropyl)methanol (*cis*-**49**).

4.11 Preparative scale procedure for enzymatic reductive cyclization reactions

These experiments were executed under complete exclusion of light in a 50 mL Greiner vial with screw cap. An aliquot of enzyme (5 µM final concentration) was rehydrated in NaPi buffer (50 mM, 150 mMNaCl, pH 7.5) to a volume of 23.25 mL. The reaction was started by the addition of a stock solution (23.25 mL) containing the substrate (10 mM), NADH (15 mM), DMF (1 % final concentration), and 1,2 DME as an internal standard ($V_{Substrate}$=$V_{InternalStandard}$). The flask was incubated for 180 min at 25 °C and 300 rpm in an Eppendorf Thermomixer comfort. For work-up Et_2O (50 mL) was added, intensely mixed, and centrifuged for 10 min at 4000 rpm. The organic layer was then separated, 20 mL AcOH and 0.9 eq* 2,4-dinitrophenylhydrazine were added and the orange solution was stirred for 48 h at 22 °C. The solvent was evaporated under reduced pressure and the 2,4-dinitrophenylhydrazone adduct was purified as stated for each compound.

*If an excess of 2,4-dinitrophenylhydrazine was used, purification of the corresponding 2,4-dinitrophenylhydrazone was not possible.

4.11.1 1-(Cyclopropylmethylene)-2-(2,4-dinitrophenyl)hydrazine (55)

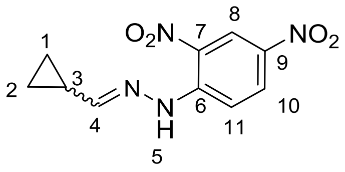

55

Starting from(*E*)-4-bromobut-2-enal (78 mg, 480 µmol) reductive cyclization with YqjM Y169F and conversion of the aldehyde with 2,4-dinitrophenylhydrazine was executed as described in4.11. The product was purified via flash chromatography (30 g silica gel, 9×3.5 cm, cyclohexane/ethylacetate 5/1, fraction size: 15 mL).

yield:71 mg (60 %), orange powder, $C_{10}H_9N_4O_4$, [250.07 g/mol]

R_f= 0.56 (cyclohexane/ethylacetate 5/1) (UV and $KMnO_4$)

HPLC-MS (**method_1, ESI$^+$**): t_R= 6.9 min;*m/z:* 251 [M+H$^+$]; λ_{max}= 210 nm.

^{1}H-NMR (300.36 MHz, DMSO-d$_6$): δ= 11.32 (s, 1H, H-5), 8.84 (d, $^{3}J_{HH}$= 2.5 Hz, 1H, H-4), 8.32 (dd, $^{3}J_{HH}$= 9.7, 2.2 Hz, 1H, H-8), 7.86 (d, $^{3}J_{HH}$= 9.6 Hz, 1H, H-10), 7.56 (d, $^{3}J_{HH}$= 7.9 Hz, 1H, H-11), 1.85–1.63 (m, 1H, H-3), 0.99 (d, $^{3}J_{HH}$= 5.5 Hz, 2H, H-1), 0.79 (d, $^{3}J_{HH}$= 2.1 Hz, 2H, H-2) ppm.

^{13}C-NMR (75.53 MHz, DMSO-d$_6$): δ= 158.5 (C-4), 144.4 (C-6), 136.2 (C-9), 129.8 (C-10), 128.3 (C-7), 123.1 (C-8), 116.2 (C-11), 14.0 (C-3), 6.7 (C-1, C-2) ppm.

m.p.: 185-187 °C

The recorded spectra are in accordance with the reported in literature.[39]

4.12 Generation of enzyme variants of OPR3 and YqjM

For generation of enzyme variants of OPR3 and YqjM, suitable mutagenesis primers were designed (see Table) and orderedat Sigma Aldrich. A PCR-based site-directed mutagenesis was performed according to the manual of the *QuikChange*™ site-directed mutagenesis kit (Stratagene) using the plasmids pET21d-*opr3* and pET21a-*yqjM* as templates.[40,41]After DpnI digestion, which was used to remove all methylated template DNA, and transformation into *E.coli* Top10 cells for selection, plasmid DNA from three colonies, each, was isolated and sequenced. Plasmids exhibiting the desired mutations were transformed into the *E.coli* expression strain BL21-CodonPlus™-(DE3)-RIL (Stratagene).

Table S3: Mutagenesis primers of OPR3 and YqjM.

Entry	Enzyme variant	Mutagenesis primer 5'-3'
1	OPR3 Y190F	ccatggagctcacggtttcttgattgatcaattcttgaaagatgg
2	OPR3 Y190W	ccatggagctcacggttggttgattgatcaattcttgaaagatgg
3	YqjM Y169F	c atgcggcgcacggattttaattcatgaatttttgtctccgc
4	YqjM Y169W	cgcacggatggttaattcatgaatttttg

4.12.1 DNA sequences and translated amino acid sequenesof ene-reductase variants

The gene of OPR3 wild type (*Lycopersiconesculentum*, tomato) with the N-terminal 6x His-tag underlined. The codon for Y190 in the gene sequence and the corresponding amino acid in the protein are highlighted with yellow color.

ATGGCTAGCCACCACCACCACCACCACATGGCGTCTTCAGCTCAAGATGGAAACA
ATCCCCTTTTCTCTCCTTACAAGATGGGCAAGTTCAATCTATCCCACAGGGTAGTA
TTGGCTCCGATGACAAGGTGCAGAGCACTGAATAATATTCCACAGGCGGCGCTAG
GGGAGTATTACGAGCAGAGAGCGACGGCCGGTGGATTTCTGATCACTGAAGGCA
CTATGATTTCTCCGACTTCAGCTGGGTTTCCTCATGTGCCAGGGATTTTCACAAAG
GAACAAGTAAGGGAATGGAAGAAAATAGTTGATGTAGTGCATGCAAAGGGTGCT
GTCATATTTTGTCAGCTGTGGCATGTTGGTCGTGCATCTCATGAAGTGTATCAACC
TGCTGGAGCTGCACCAATATCATCCACTGAGAAGCCTATATCAAATAGGTGGAGA
ATTCTAATGCCTGATGGAACTCATGGGATTTATCCAAAACCAAGAGCAATTGGAA
CCTATGAGATCTCACAAGTTGTTGAAGATTATCGCAGGTCGGCCTTGAATGCTAT
TGAAGCAGGTTTCGATGGTATTGAAATCCATGGAGCTCACGGTTACTTGATTGAT
CAATTCTTGAAAGATGGGATCAATGACCGGACAGATGAGTATGGTGGATCACTA
GCCAACCGGTGCAAATTCATCACACAGGTGGTTCAAGCAGTAGTCTCAGCAATAG
GAGCTGATCGCGTAGGCGTTAGAGTTTCACCAGCAATAGATCATCTTGATGCCAT
GGACTCTAATCCACTCAGCCTTGGCTTAGCAGTTGTTGAAAGACTAAACAAAATC
CAACTCCATTCTGGTTCCAAGCTTGCCTATCTTCATGTAACACAGCCACGATACGT
AGCATATGGGCAAACTGAAGCAGGCAGACTTGGCAGTGAAGAGGAAGAGGCTCG
TTTAATGAGGACTTTGAGGAACGCGTATCAGGGGACATTCATTTGCAGTGGTGGA
TACACTAGGGAACTAGGAATTGAGGCTGTGGCACAAGGTGATGCTGATCTCGTGT
CATATGGTCGTCTTTTCATCTCTAATCCTGATTTGGTTATGAGAATCAAGCTAAAT
GCACCTCTAAATAAGTATAACAGGAAGACATTCTATACTCAAGATCCAGTTGTGG
GATACACAGATTACCCTTTCCTTCAAGGAAATGGAAGCAATGGACCGTTATCGCG
TCTGTGA

MASHHHHHHMASSAQDGNNPLFSPYKMGKFNLSHRVVLAPMTRCRALNNIPQAAL
GEYYEQRATAGGFLITEGTMISPTSAGFPHVPGIFTKEQVREWKKIVDVVHAKGAVIF
CQLWHVGRASHEVYQPAGAAPISSTEKPISNRWRILMPDGTHGIYPKPRAIGTYEISQ
VVEDYRRSALNAIEAGFDGIEIHGAHG**Y**LIDQFLKDGINDRTDEYGGSLANRCKFITQ
VVQAVVSAIGADRVGVRVSPAIDHLDAMDSNPLSLGLAVVERLNKIQLHSGSKLAYL
HVTQPRYVAYGQTEAGRLGSEEEEARLMRTLRNAYQGTFICSGGYTRELGIEAVAQG

DADLVSYGRLFISNPDLVMRIKLNAPLNKYNRKTFYTQDPVVGYTDYPFLQGNGSNG
PLSRL

The gene of OPR3 Y190F variant with the N-terminal 6x His-tag underlined and mutagenesis primer in bold. The codon for Y190F in the gene sequence and the corresponding amino acid in the protein are highlighted with yellow color.

ATGGCTAGCCACCACCACCACCACCACATGGCGTCTTCAGCTCAAGATGGAAACA
ATCCCCTTTTCTCTCCTTACAAGATGGGCAAGTTCAATCTATCCCACAGGGTAGTA
TTGGCTCCGATGACAAGGTGCAGAGCACTGAATAATATTCCACAGGCGGCGCTAG
GGGAGTATTACGAGCAGAGAGCGACGGCCGGTGGATTTCTGATCACTGAAGGCA
CTATGATTTCTCCGACTTCAGCTGGGTTTCCTCATGTGCCAGGGATTTTCACAAAG
GAACAAGTAAGGGAATGGAAGAAAATAGTTGATGTAGTGCATGCAAAGGGTGCT
GTCATATTTTGTCAGCTGTGGCATGTTGGTCGTGCATCTCATGAAGTGTATCAACC
TGCTGGAGCTGCACCAATATCATCCACTGAGAAGCCTATATCAAATAGGTGGAGA
ATTCTAATGCCTGATGGAACTCATGGGATTTATCCAAAACCAAGAGCAATTGGAA
CCTATGAGATCTCACAAGTTGTTGAAGATTATCGCAGGTCGGCCTTGAATGCTAT
TGAAGCAGGTTTCGATGGTATTGAAAT**CCATGGAGCTCACGGTTTCTTGATTGA**
TCAATTCTTGAAAGATGGGATCAATGACCGGACAGATGAGTATGGTGGATCACT
AGCCAACCGGTGCAAATTCATCACACAGGTGGTTCAAGCAGTAGTCTCAGCAATA
GGAGCTGATCGCGTAGGCGTTAGAGTTTCACCAGCAATAGATCATCTTGATGCCA
TGGACTCTAATCCACTCAGCCTTGGCTTAGCAGTTGTTGAAAGACTAAACAAAAT
CCAACTCCATTCTGGTTCCAAGCTTGCCTATCTTCATGTAACACAGCCACGATACG
TAGCATATGGGCAAACTGAAGCAGGCAGACTTGGCAGTGAAGAGGAAGAGGCTC
GTTTAATGAGGACTTTGAGGAACGCGTATCAGGGGACATTCATTTGCAGTGGTGG
ATACACTAGGGAACTAGGAATTGAGGCTGTGGCACAAGGTGATGCTGATCTCGTG
TCATATGGTCGTCTTTTCATCTCTAATCCTGATTTGGTTATGAGAATCAAGCTAAA
TGCACCTCTAAATAAGTATAACAGGAAGACATTCTATACTCAAGATCCAGTTGTG
GGATACACAGATTACCCTTTCCTTCAAGGAAATGGAAGCAATGGACCGTTATCGC
GTCTGTGA

MASHHHHHHMASSAQDGNNPLFSPYKMGKFNLSHRVVLAPMTRCRALNNIPQAAL
GEYYEQRATAGGFLITEGTMISPTSAGFPHVPGIFTKEQVREWKKIVDVVHAKGAVIF
CQLWHVGRASHEVYQPAGAAPISSTEKPISNRWRILMPDGTHGIYPKPRAIGTYEISQ
VVEDYRRSALNAIEAGFDGIEIHGAHG**F**LIDQFLKDGINDRTDEYGGSLANRCKFITQ
VVQAVVSAIGADRVGVRVSPAIDHLDAMDSNPLSLGLAVVERLNKIQLHSGSKLAYL

HVTQPRYVAYGQTEAGRLGSEEEEARLMRTLRNAYQGTFICSGGYTRELGIEAVAQG
DADLVSYGRLFISNPDLVMRIKLNAPLNKYNRKTFYTQDPVVGYTDYPFLQGNGSNG
PLSRL

The gene of OPR3 Y190W variant with the N-terminal 6x His-tag underlined and mutagenesis primer in bold. The codon for Y190W in the gene sequence and the corresponding amino acid in the protein are highlighted with yellow color.

ATGGCTAGCCACCACCACCACCACCACATGGCGTCTTCAGCTCAAGATGGAAACA
ATCCCCTTTTCTCTCCTTACAAGATGGGCAAGTTCAATCTATCCCACAGGGTAGTA
TTGGCTCCGATGACAAGGTGCAGAGCACTGAATAATATTCCACAGGCGGCGCTAG
GGGAGTATTACGAGCAGAGAGCGACGGCCGGTGGATTTCTGATCACTGAAGGCA
CTATGATTTCTCCGACTTCAGCTGGGTTTCCTCATGTGCCAGGGATTTTCACAAAG
GAACAAGTAAGGGAATGGAAGAAAATAGTTGATGTAGTGCATGCAAAGGGTGCT
GTCATATTTTGTCAGCTGTGGCATGTTGGTCGTGCATCTCATGAAGTGTATCAACC
TGCTGGAGCTGCACCAATATCATCCACTGAGAAGCCTATATCAAATAGGTGGAGA
ATTCTAATGCCTGATGGAACTCATGGGATTTATCCAAAACCAAGAGCAATTGGAA
CCTATGAGATCTCACAAGTTGTTGAAGATTATCGCAGGTCGGCCTTGAATGCTAT
TGAAGCAGGTTTCGATGGTATTGAAAT**CCATGGAGCTCACGGTTGGTTGATTG
ATCAATTCTTGAAAGATGG**GATCAATGACCGGACAGATGAGTATGGTGGATCAC
TAGCCAACCGGTGCAAATTCATCACACAGGTGGTTCAAGCAGTAGTCTCAGCAAT
AGGAGCTGATCGCGTAGGCGTTAGAGTTTCACCAGCAATAGATCATCTTGATGCC
ATGGACTCTAATCCACTCAGCCTTGGCTTAGCAGTTGTTGAAAGACTAAACAAAA
TCCAACTCCATTCTGGTTCCAAGCTTGCCTATCTTCATGTAACACAGCCACGATAC
GTAGCATATGGGCAAACTGAAGCAGGCAGACTTGGCAGTGAAGAGGAAGAGGCT
CGTTTAATGAGGACTTTGAGGAACGCGTATCAGGGGACATTCATTTGCAGTGGTG
GATACACTAGGGAACTAGGAATTGAGGCTGTGGCACAAGGTGATGCTGATCTCGT
GTCATATGGTCGTCTTTTCATCTCTAATCCTGATTTGGTTATGAGAATCAAGCTAA
ATGCACCTCTAAATAAGTATAACAGGAAGACATTCTATACTCAAGATCCAGTTGT
GGGATACACAGATTACCCTTTCCTTCAAGGAAATGGAAGCAATGGACCGTTATCG
CGTCTGTGA

MASHHHHHHMASSAQDGNNPLFSPYKMGKFNLSHRVVLAPMTRCRALNNIPQAAL
GEYYEQRATAGGFLITEGTMISPTSAGFPHVPGIFTKEQVREWKKIVDVVHAKGAVIF
CQLWHVGRASHEVYQPAGAAPISSTEKPISNRWRILMPDGTHGIYPKPRAIGTYEISQ
VVEDYRRSALNAIEAGFDGIEIHGAHGWLIDQFLKDGINDRTDEYGGSLANRCKFITQ

VVQAVVSAIGADRVGVRVSPAIDHLDAMDSNPLSLGLAVVERLNKIQLHSGSKLAYL
HVTQPRYVAYGQTEAGRLGSEEEEARLMRTLRNAYQGTFICSGGYTRELGIEAVAQG
DADLVSYGRLFISNPDLVMRIKLNAPLNKYNRKTFYTQDPVVGYTDYPFLQGNGSNG
PLSRL

The gene of YqjM wild type (*Bacillus subtilis*). The codon for Y169 in the gene sequence and the corresponding amino acid in the protein are highlighted with yellow color.

ATGGCCAGAAAATTATTTACACCTATTACAATTAAAGATATGACGTTAAAAAACC
GCATTGTCATGTCGCCAATGTGCATGTATTCTTCTCATGAAAAGGACGGAAAATT
AACACCGTTCCACATGGCACATTACATATCGCGCGCAATCGGCCAGGTCGGACTG
ATTATTGTAGAGGCGTCAGCGGTTAACCCTCAAGGACGAATCACTGACCAAGACT
TAGGCATTTGGAGCGACGAGCATATTGAAGGCTTTGCAAAACTGACTGAGCAGGT
CAAAGAACAAGGTTCAAAAATCGGCATTCAGCTTGCCCATGCCGGACGTAAAGC
TGAGCTTGAAGGAGATATCTTCGCTCCATCGGCGATTGCGTTTGACGAACAATCA
GCAACACCTGTAGAAATGTCAGCAGAAAAAGTAAAAGAAACGGTCCAGGAGTTC
AAGCAAGCGGCTGCCCGCGCAAAAGAAGCCGGCTTTGATGTGATTGAAATTCAT
GCGGCGCACGGATATTTAATTCATGAATTTTTGTCTCCGCTTTCCAACCATCGAAC
AGATGAATATGGCGGCTCACCTGAAAACCGCTATCGTTTCTTGAGAGAGATCATT
GATGAAGTCAAACAAGTATGGGACGGTCCTTTATTTGTCCGTGTATCTGCTTCTGA
CTACACTGATAAAGGCTTAGACATTGCCGATCACATCGGTTTTGCAAAATGGATG
AAGGAGCAGGGTGTTGACTTAATTGACTGCAGCTCAGGCGCCCTTGTTCACGCAG
ACATTAACGTATTCCCTGGCTATCAGGTCAGCTTCGCTGAGAAAATCCGTGAACA
GGCGGACATGGCTACTGGTGCCGTCGGCATGATTACAGACGGTTCAATGGCTGAA
GAAATTCTGCAAAACGGACGTGCCGACCTCATCTTTATCGGCAGAGAGCTTTTGC
GGGATCCATTTTTTGCAAGAACTGCTGCGAAACAGCTCAATACAGAGATTCCGGC
CCCTGTTCAATACGAAAGAGGCTGGTAA

MARKLFTPITIKDMTLKNRIVMSPMCMYSSHEKDGKLTPFHMAHYISRAIGQVGLIIV
EASAVNPQGRITDQDLGIWSDEHIEGFAKLTEQVKEQGSKIGIQLAHAGRKAELEGDI
FAPSAIAFDEQSATPVEMSAEKVKETVQEFKQAAARAKEAGFDVIEIHAAHGYLIHEF
LSPLSNHRTDEYGGSPENRYRFLREIIDEVKQVWDGPLFVRVSASDYTDKGLDIADHI
GFAKWMKEQGVDLIDCSSGALVHADINVFPGYQVSFAEKIREQADMATGAVGMITD
GSMAEEILQNGRADLIFIGRELLRDPFFARTAAKQLNTEIPAPVQYERGW

The gene of YqjM Y169F variant with the mutagenesis primer in bold. The codon for Y169F in the gene sequence and the corresponding amino acid in the protein are highlighted with yellow color.

ATGGCCAGAAAATTATTTACACCTATTACAATTAAAGATATGACGTTAAAAAACC
GCATTGTCATGTCGCCAATGTGCATGTATTCTTCTCATGAAAAGGACGGAAAATT
AACACCGTTCCACATGGCACATTACATATCGCGCGCAATCGGCCAGGTCGGACTG
ATTATTGTAGAGGCGTCAGCGGTTAACCCTCAAGGACGAATCACTGACCAAGACT
TAGGCATTTGGAGCGACGAGCATATTGAAGGCTTTGCAAAACTGACTGAGCAGGT
CAAAGAACAAGGTTCAAAAATCGGCATTCAGCTTGCCCATGCCGGACGTAAAGC
TGAGCTTGAAGGAGATATCTTCGCTCCATCGGCGATTGCGTTTGACGAACAATCA
GCAACACCTGTAGAAATGTCAGCAGAAAAAGTAAAAGAAACGGTCCAGGAGTTC
AAGCAAGCGGCTGCCCGCGCAAAAGAAGCCGGCTTTGATGTGATTGAAATT**CAT**
GCGGCGCACGGATTTTTAATTCATGAATTTTTGTCTCCGCTTTCCAACCATCG
AACAGATGAATATGGCGGCTCACCTGAAAACCGCTATCGTTTCTTGAGAGAGATC
ATTGATGAAGTCAAACAAGTATGGGACGGTCCTTTATTTGTCCGTGTATCTGCTTC
TGACTACACTGATAAAGGCTTAGACATTGCCGATCACATCGGTTTTGCAAAATGG
ATGAAGGAGCAGGGTGTTGACTTAATTGACTGCAGCTCAGGCGCCCTTGTTCACG
CAGACATTAACGTATTCCCTGGCTATCAGGTCAGCTTCGCTGAGAAAATCCGTGA
ACAGGCGGACATGGCTACTGGTGCCGTCGGCATGATTACAGACGGTTCAATGGCT
GAAGAAATTCTGCAAAACGGACGTGCCGACCTCATCTTTATCGGCAGAGAGCTTT
TGCGGGATCCATTTTTTGCAAGAACTGCTGCGAAACAGCTCAATACAGAGATTCC
GGCCCCTGTTCAATACGAAAGAGGCTGGTAA

MARKLFTPITIKDMTLKNRIVMSPMCMYSSHEKDGKLTPFHMAHYISRAIGQVGLIIV
EASAVNPQGRITDQDLGIWSDEHIEGFAKLTEQVKEQGSKIGIQLAHAGRKAELEGDI
FAPSAIAFDEQSATPVEMSAEKVKETVQEFKQAAARAKEAGFDVIEIHAAHGFLIHEF
LSPLSNHRTDEYGGSPENRYRFLREIIDEVKQVWDGPLFVRVSASDYTDKGLDIADHI
GFAKWMKEQGVDLIDCSSGALVHADINVFPGYQVSFAEKIREQADMATGAVGMITD
GSMAEEILQNGRADLIFIGRELLRDPFFARTAAKQLNTEIPAPVQYERGW

The gene of YqjM Y169W variant with the mutagenesis primer in bold. The codon for Y169W in the gene sequence and the corresponding amino acid in the protein are highlighted with yellow color.

ATGGCCAGAAAATTATTTACACCTATTACAATTAAAGATATGACGTTAAAAAACC
GCATTGTCATGTCGCCAATGTGCATGTATTCTTCTCATGAAAAGGACGGAAAATT
AACACCGTTCCACATGGCACATTACATATCGCGCGCAATCGGCCAGGTCGGACTG
ATTATTGTAGAGGCGTCAGCGGTTAACCCTCAAGGACGAATCACTGACCAAGACT
TAGGCATTTGGAGCGACGAGCATATTGAAGGCTTTGCAAAACTGACTGAGCAGGT
CAAAGAACAAGGTTCAAAAATCGGCATTCAGCTTGCCCATGCCGGACGTAAAGC
TGAGCTTGAAGGAGATATCTTCGCTCCATCGGCGATTGCGTTTGACGAACAATCA
GCAACACCTGTAGAAATGTCAGCAGAAAAAGTAAAAGAAACGGTCCAGGAGTTC
AAGCAAGCGGCTGCCCGCGCAAAAGAAGCCGGCTTTGATGTGATTGAAATTCAT
GCGG**CGCACGGATGGTTAATTCATGAATTTTTG**TCTCCGCTTTCCAACCATCGA
ACAGATGAATATGGCGGCTCACCTGAAAACCGCTATCGTTTCTTGAGAGAGATCA
TTGATGAAGTCAAACAAGTATGGGACGGTCCTTTATTTGTCCGTGTATCTGCTTCT
GACTACACTGATAAAGGCTTAGACATTGCCGATCACATCGGTTTTGCAAAATGGA
TGAAGGAGCAGGGTGTTGACTTAATTGACTGCAGCTCAGGCGCCCTTGTTCACGC
AGACATTAACGTATTCCCTGGCTATCAGGTCAGCTTCGCTGAGAAAATCCGTGAA
CAGGCGGACATGGCTACTGGTGCCGTCGGCATGATTACAGACGGTTCAATGGCTG
AAGAAATTCTGCAAAACGGACGTGCCGACCTCATCTTTATCGGCAGAGAGCTTTT
GCGGGATCCATTTTTTGCAAGAACTGCTGCGAAACAGCTCAATACAGAGATTCCG
GCCCCTGTTCAATACGAAAGAGGCTGGTAA

MARKLFTPITIKDMTLKNRIVMSPMCMYSSHEKDGKLTPFHMAHYISRAIGQVGLIIV
EASAVNPQGRITDQDLGIWSDEHIEGFAKLTEQVKEQGSKIGIQLAHAGRKAELEGDI
FAPSAIAFDEQSATPVEMSAEKVKETVQEFKQAAARAKEAGFDVIEIHAAHGWLIHEF
LSPLSNHRTDEYGGSPENRYRFLREIIDEVKQVWDGPLFVRVSASDYTDKGLDIADHI
GFAKWMKEQGVDLIDCSSGALVHADINVFPGYQVSFAEKIREQADMATGAVGMITD
GSMAEEILQNGRADLIFIGRELLRDPFFARTAAKQLNTEIPAPVQYERGW

4.12.2 Preparation of buffers

NaH_2PO_4/Na_2HPO_4buffer (50 mM, pH 7.5, 150 mM NaCl)

$Na_2HPO_4 \cdot 2H_2O$ (8.29 g, 46.6 mmol), $NaH_2PO_4 \cdot H_2O$ (0.47 g, 3.4 mmol) and NaCl (8.77 g, 150 mmol) were dissolved in 1 L H_2O bidest. Under vigorous stirring H_3PO_4 conc. orNaOH-solution (conc., in H_2O)were added until a calibrated pH-meter indicated pH 7.5. Buffer solution was filtered.

For buffers additionally containing imidazole, the latter was added prior to the adjustment of the pH.

Buffer A: Tris-HCl buffer (50 mM, pH 7.5)

Tris-HCl (7.88 g, 50 mmol) was dissolved in 1 L H_2O bidest. Under vigorous stirring HCl conc. or NaOH-solution (conc., in H_2O) were added until a calibrated pH-meter indicated pH 7.5. Buffer solution was filtered.

Buffer B: Tris-HCl buffer (50 mM, pH 7.5, 400 mMKCl)

Tris-HCl (7.88 g, 50 mmol) and KCl (29.8 g, 400 mmol) were dissolved in 1 L H_2O bidest. Under vigorous stirring HCl conc. or NaOH-solution (conc., in H_2O) were added until a calibrated pH-meter indicated pH 7.5. Buffer solution was filtered.

Buffer C: Tris-HCl buffer (50 mM, pH 7.5, 1.5 M $(NH_4)_2SO_4$)

Tris-HCl (7.88 g, 50 mmol) and $(NH_4)_2SO_4$(198.2 g, 1.5 mol) were dissolved in 1 L H_2O bidest. Under vigorous stirring HCl conc. or NaOH-solution (conc., in H_2O) were added until a calibrated pH-meter indicated pH 7.5. Buffer solution was filtered.

4.12.3 Expression of OPR3 and YqjM wild types and variants

10 mL of an overnight cell culture was used to inoculate one flask with 800 mL LB medium supplemented with ampicillin (100 µg/mL) and chloramphenicol (20 µg/mL).The cell culture was incubated at 37 °C at 150 rpm until an OD_{600} of 0.6-0.8 was reached. Then, protein expression was induced by the addition of IPTG (to a final concentration of 0.125 mM) and the cultures were incubated at 20 °C and 150 rpm for 16 h. The cells were harvested by centrifugation at 5000 rpm and 4 °C (for 15 min) and the resulting pellets were stored at -32 °C.

4.12.4 Purification of OPR3 wild type and variants

A cell pellet (~ 12g) was resuspended in lysis buffer (50 mM sodium phosphate buffer, pH 7.5, 20 mM imidazole, 150 mMNaCl, 3 mL/g cell pellet), to whichphenylmethylsulfonyl fluoride (final concentration 1.3 mM) and lysozyme (final concentration 1 mg/mL) were added. After 20 min of incubation at 4 °C the cells were further lysed by ultra-sonication for 10 min (0.5 s pulse, power 60 %). The sonicated suspension was subsequently centrifuged at 20.000 rpm and 4 °C for 45 min. The supernatant was then loaded onto a Ni-NTA HisTrap FF 5 mL column equilibrated with lysis buffer. After washing with increasing concentrations of imidazole (up to 50 mM), the bound protein was eluted with elution buffer (50 mM sodium phosphate buffer, pH 7.5, 300 mM imidazole, 150 mMNaCl). The yellow fractions containing OPR3 were pooled, concentrated, and dialyzed against sodium phosphate buffer (50 mM, pH 7.5, 150 mMNaCl) at 4 °C overnight. If required, the protein was then again concentrated and aliquots of OPR3 WT and its variants were stored at 4°C until needed.In addition, the purity of the protein fractions was analyzed by SDS-PAGE.

4.12.5 Purification of YqjM wild type and variants[41]

A cell pellet (10-15 g) was resuspended in buffer A (50 mMTris-HCl, pH 7.5, 4 mL/g cell pellet), to whichphenylmethylsulfonyl fluoride (final concentration 1.3 mM) and lysozyme (final concentration 1 mg/mL) were added. After 20 min of incubation at 4 °C the cells were further lysed by ultra-sonication for 10 min (0.5 s pulse, power 60 %). The sonicated suspension was centrifuged at 20.000 rpm and 4 °C for 45 min. The supernatant was then loaded onto an ion exchange chromatography DEAE-Sephacel FF column (20 mL) equilibrated with buffer A. After extensive washing with buffer A, the bound protein was eluted with a linear gradient of 250 mL of buffer A and buffer B (buffer B: 50 mMTris-HCl, pH 7.5, 400 mMKCl). The yellow fractions containing YqjM were pooled, concentrated, and dialyzed against buffer A at 4 °C overnight. The dialysate was brought to 30 % ammonium sulfate saturation and applied to a phenyl-sepharosecolumn (25 mL) freshly equilibrated with buffer C (50 mMTris-HCl, pH 7.5, 1.5 M $(NH_4)_2SO_4$). After washing with buffer C, the bound protein was eluted with a linear gradient of 500 mL buffer C and buffer A. The yellow fractions containing YqjM were again pooled, concentrated, and dialyzedtagainst buffer A at 4 °C overnight. If necessary, the protein solution was again concentrated, before flash freezing aliquots of YqjM wild type and variants with liquid nitrogen and storing them at –

32°C until further use. To prove the quality of the protein purification SDS-PAGE analysis was applied after each chromatographic step.

4.12.6 Estimation of enzyme concentrations based on absorption spectra

Enzyme concentrations were photometrically estimated using the molar extinction coefficients of OPR3 wild type and YqjM wild type at their respective absorption maximum ($\varepsilon_{\lambda max}$ = 11600 $M^{-1}\cdot cm^{-1}$; see Table).[41,42]

Table S4: Absorption maxima of OPR3 and YqjM wild type enzymes and variants.

Entry	Enzyme variant	Absorption maximum [nm]
1	OPR3 WT	466
2	OPR3 Y190F	467
3	OPR3 Y190W	466
4	YqjM WT	455
5	YqjM Y169F	454
6	YqjM Y169W	454

All spectra were recorded using a spectrophotometer Specord 200 plus from Analytik Jena. The measurements were carried out in disposable cuvettes (semimicro, dimension 12.5×12.5×45 mm) from Brand. Spectra are shown in Figure S10, Figure S11, Figure S12, Figure S13, Figure S14 and Figure S15.

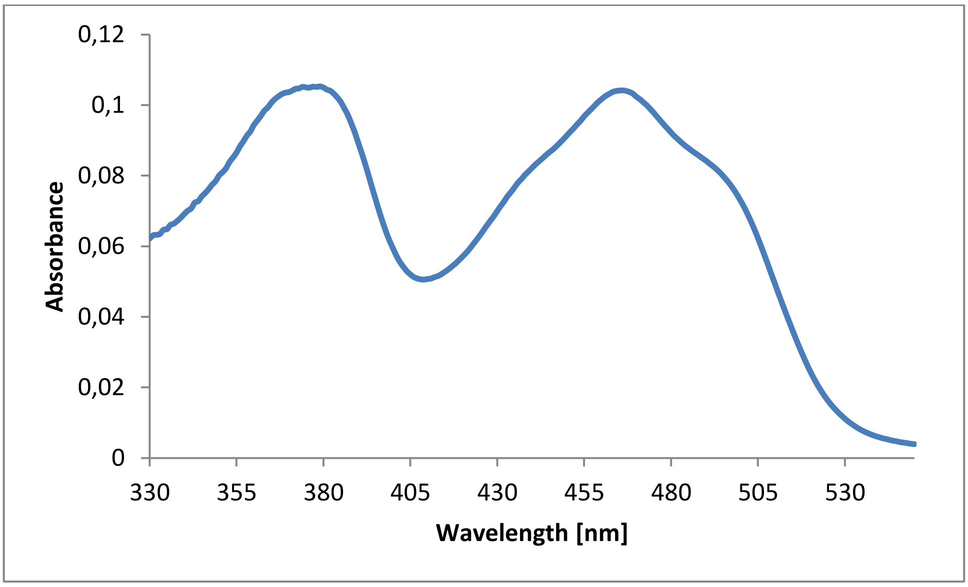

Figure S10: Absorption spectrum of OPR3 WT.

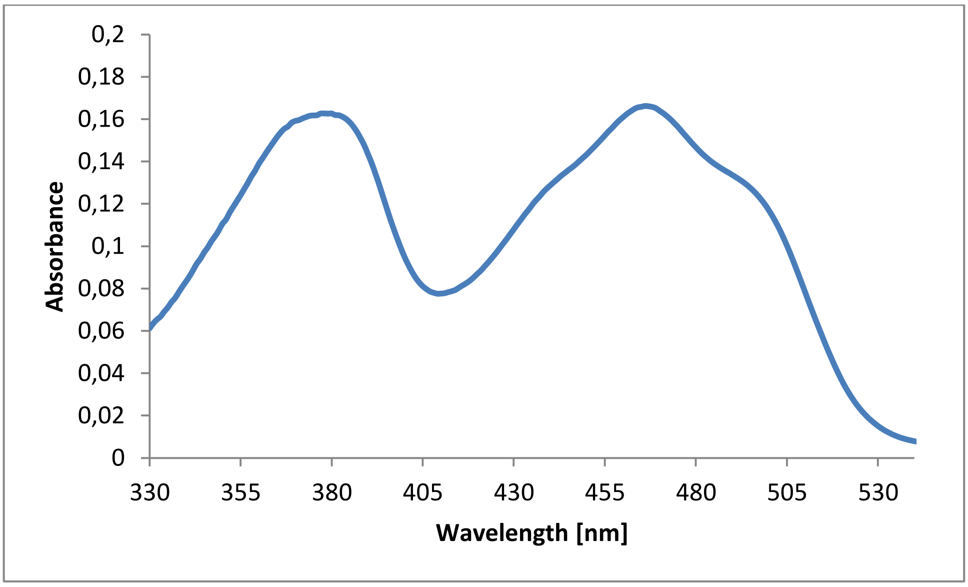

Figure S11: Absorption spectrum of OPR3 Y190F.

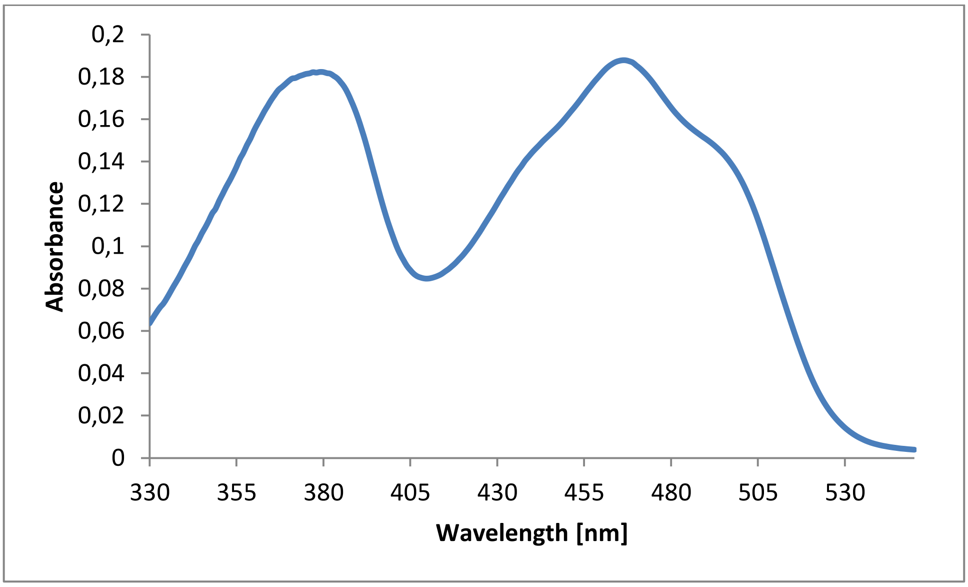

Figure S12: Absorption spectrum of OPR3 Y190W.

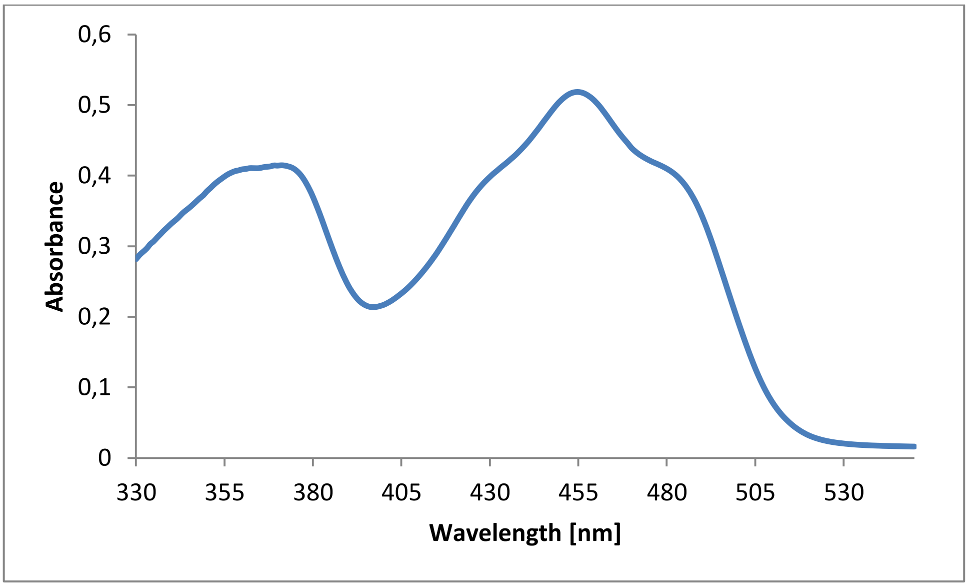

Figure S13: Absorption spectrum of YqjM WT.

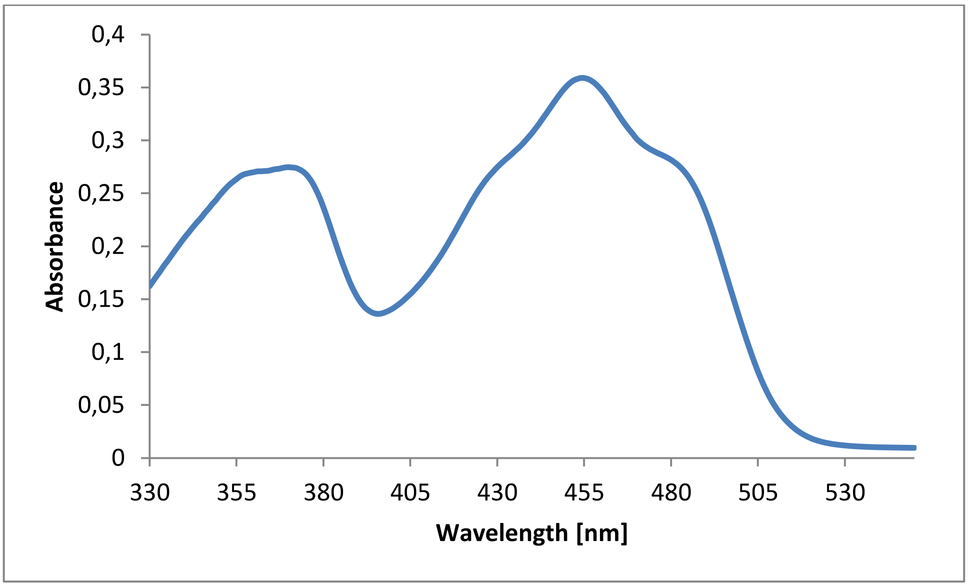

Figure S14: Absorption spectrum of YqjM Y169F.

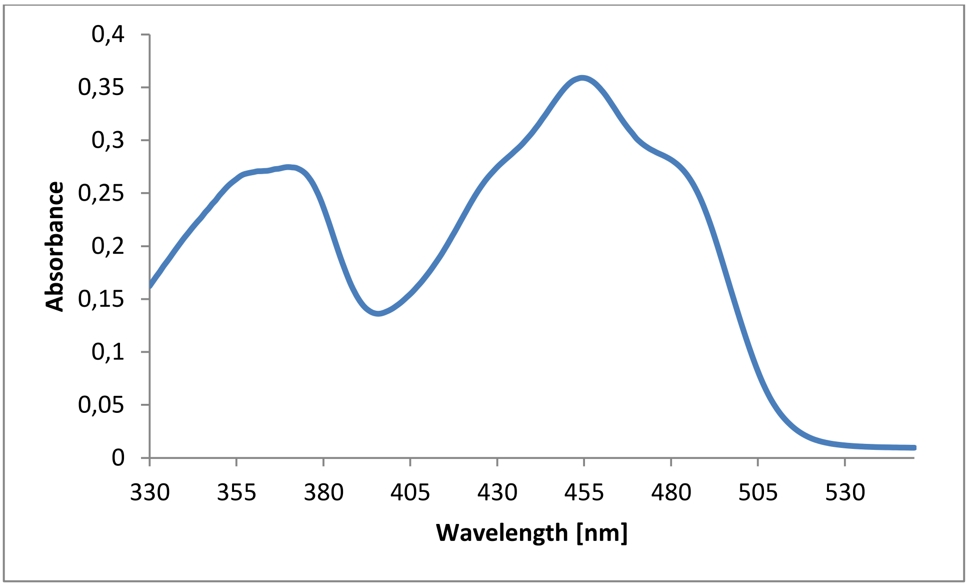

Figure S15: Absorption spectrum of YqjM Y169W.

5 References

[1] A. D. Bochevarov, E. Harder, T.F. Hughes, J. R. Greenwood, D. A. Braden, D. M. Philipp, D. Rinaldo, M. D. Halls, J. Zhang, R. A. Friesner, R.A., *Int. J. Quantum Chem.,***2013**, *113*, 2110-2142.
[2] O. Trott, A. J. Olson, *J. Comput. Chem.***2010**, *31*, 455-461.
[3] S. Sadhukhan, G. F. Zhang, G. P. Tochtrop, *ACS Chem. Biol.* **2014**, *9*, 1706-1711.
[4] M. P. Doyle, M. Yan, W. Hu, L. S. Gronenberg, *J. Am. Chem. Soc***2003**, *125*, 4692-4693.
[5] D. J. Phillips, A. E. Graham, *Synlett***2008**, *5*, 649-652.
[6] R. A. Bunce, D. A. Irwin, *Synth. Commun.***1990**, *20*, 2979-2982.
[7]S.Nakamura,Y. Watanabe, T. Toru, *J. Org. Chem.***2000**, *65*, 1758-1766.
[8] M. Dischmann, T. Frassetto, M. A. Breuning, U. Koert, *Chem. Eur. J.***2014**, *20*, 11300-11302.
[9]Denmark S., T. Kobayashi, R. Christopher, *Tetrahedron***2010**, *66*, 4745-4759.
[10] Y. Nakagawa, S. Chanthamath, K. Shibatomi, S. Iwasa, *Org. Lett.* **2015**, *17*, 2792-2795.
[11] I. Shiina, Y. Umezaki, Y. Ohashi, Y. Yamazaki, S. Dan, T. Yamori, *J. Med. Chem.* **2013**, *56*, 150-159.
[12] G. E. Magoulas, S. E. Bariamis, C. M. Athanassopoulos, A. Haskopoulos, P. G. Dedes, M. G. Krokidis, N. K. Karamanos, D. Kletsas, D. Papaioannou, G. Maroulis, *Eur. J. Med. Chem.***2011**, *46*, 721-737.
[13] G. L. Olson, H. C. Cheung, K. D. Morgan, C. Neukom, G. Saucy, *J. Org. Chem.***1976**, *41*, 3287-3293.
[14]H.Lu, J. Y. Liu, C. G. Li, J. B. Lin, Y. M. Liang, P. F. Xu, *Chem. Commun.***2015**, *51*, 4473-4476.
[15] P. D. Brown, A. C. Willis, M. S. Sherburn, A. L. Lawrence, *Angew. Chem. Int. Ed.***2013**, *52*, 13273-13275.
[16] X. Han, P. E. Floreancig, *Angew. Chem. Int. Ed.***2014**, *53*, 11075-11078.
[17] D. J. Miller, F. Yu, D. W. Knight, K. Allemann, *Org. Biomol. Chem.***2009**, *7*, 962-975.
[18] D. J. Collins, A. M. James, *Aust. J. Chem.* **1989**, *45*, 223-228.
[19] M. Schmid, R. Barner, *Helv. Chim.Acta***1979**, *62*, 464-473.
[20] H. O. House, P. D. Weeks, *J. Am. Chem. Soc.***1975**, *97*, 2778-2785.
[21] J. Pietruszka, A. C. M. Rieche, T. Wilhelm, A. Witt, *Adv. Synth. Catal.***2003**, *345*, 1273-1286.
[22] M. Schmittel, A. A. Mahajan, G. Bucher, J. W. Bats, *J. Org. Chem.* **2007**, *72*, 2166-2173.
[23]S. Fan, F. Chen, X. Zhang, *Angew. Chem.Int. Ed.* **2011**, *50*, 5918-5923.
[24]S. E. Denmark, S. P. O`Connor, *J. Org. Chem.***1997**, *62*, 584-594.
[25] P. Mauleon, J. L. Krinsky, F. D. Toste, *J. Am. Chem. Soc*. **2009**, *131*, 4513–4520.
[26]J. Rehbein, S. Leick, M. Hiersemann, *J. Org. Chem.***2009**, *74*, 1531-1540.
[27] J. D. White, T. S Kim, M. Nambu, *J. Am. Chem. Soc.***1995**, *117*, 5612-5613.
[28] E. C. Friedrich, S. E. Lunetta, E. J. Lewis, *J. Org. Chem.* **1989**, *54*, 2388-2390.
[29] V. M. T. Carneiro, C. M. Avila, M. J. Balunas, W. H. Gerwick, R. A. Pilli, *J. Org. Chem.* **2014**, *79*, 630-642.
[30]R. Anthes, O. Bello, S. Benoit, C.-K. Chen, E. Corbett, R. M. Corbett, A. J. DelMonte, S. Gingras, R. Livingston, J. Sausker, M. Soumeillant, *Org. Process Res. Dev.***2008**, *12*, 168-177.
[31] M.S.C. Pedras, A. Abdoli, *Bioorg. Med. Chem.* **2013**, *21*, 4541-4549.

[32] M. J. Berenguer, J. Castells, J. Fernandez, R. M. Galard, *Tetrahedron Lett.***1971**, *6*, 493-494.
[33] L. Duhamel, J. Guillemont, J. M. Poirier, *Tetrahedron Lett.***1992**, *33*, 5051-5054.
[34] G. Eletti-Bianchi, F. Centini, L. Re, *J. Org. Chem.***1976**, *41*, 1648-1650.
[35]J. Sabbatani, N. Maulide, *Angew. Chem. Int. Ed.* **2016**, *55*, 6780-6783.
[36] A. Aponick, C. Y. Li, B. Biannic, Org. Lett. **2008**, *10*, 669-671.
[37] J. A. Bull, A. B. Charette, *J. Org. Chem.***2008**, *73*, 8097-8100.
[38] H. Shitama, T. Katsuki, *Angew. Chem. Int. Ed.* **2008**, *47*, 2450-2453.
[39] M. Lageron, M. B. Fleury, *Angew. Chem. Int. Ed.* **2012**, *57*, 5409-5412.
[40] C. Breithaupt, R. Kurzbauer, H. Lilie, A. Schaller, J. Strassner, R. Huber, P.Macheroux, T. Clausen, *Proc. Natl. Acad. Sci. U.S.A.* **2006**,*103*, 14337–14342.
[41] T. B. Fitzpatrick, N. Amrhein, P. Macheroux, *J. Biol. Chem.* **2003**, *278*, 19891-19897.
[42] J. Straßner, A. Fürholz, P. Macheroux, N. Amrhein, A. Schaller, *J. Biol. Chem*. **1999**, *274*, 35067–35073.

6 NMR-Appendix

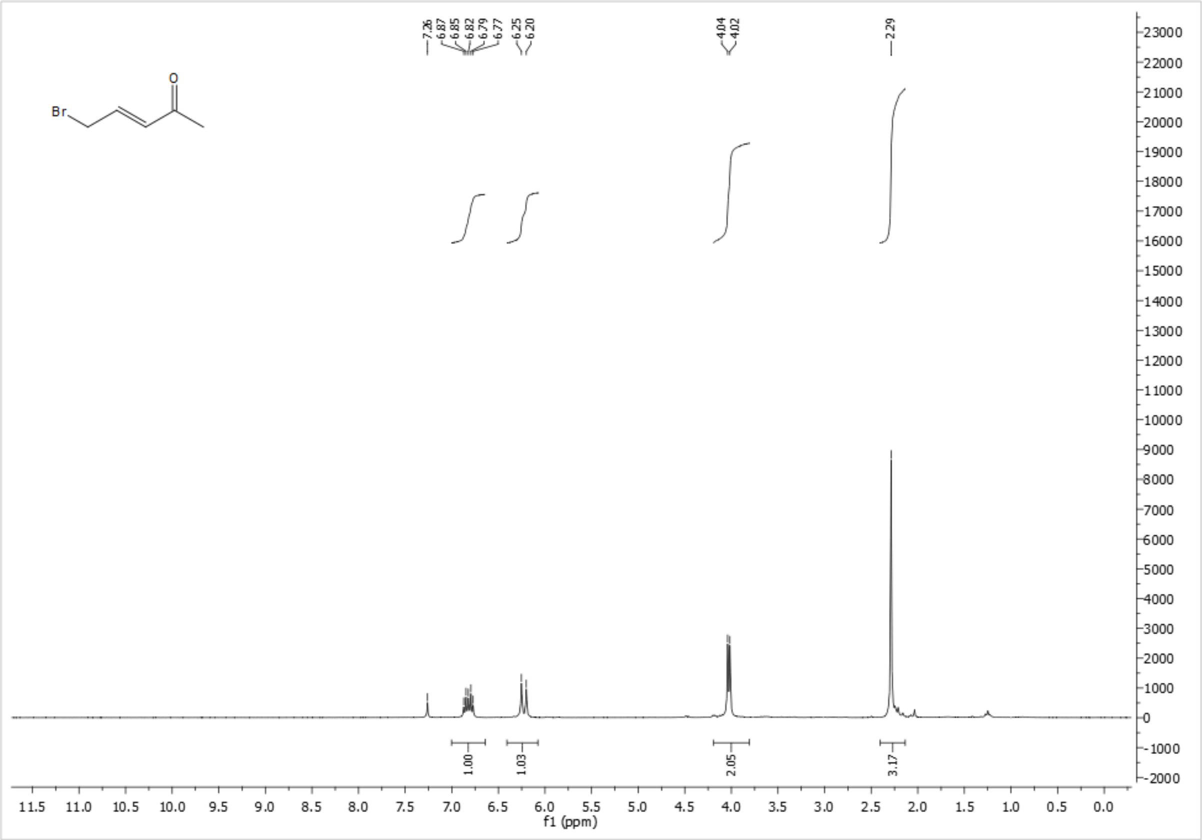

Figure S16:^{1}H-NMR (300.36 MHz, $CDCl_3$) of compound **21**.

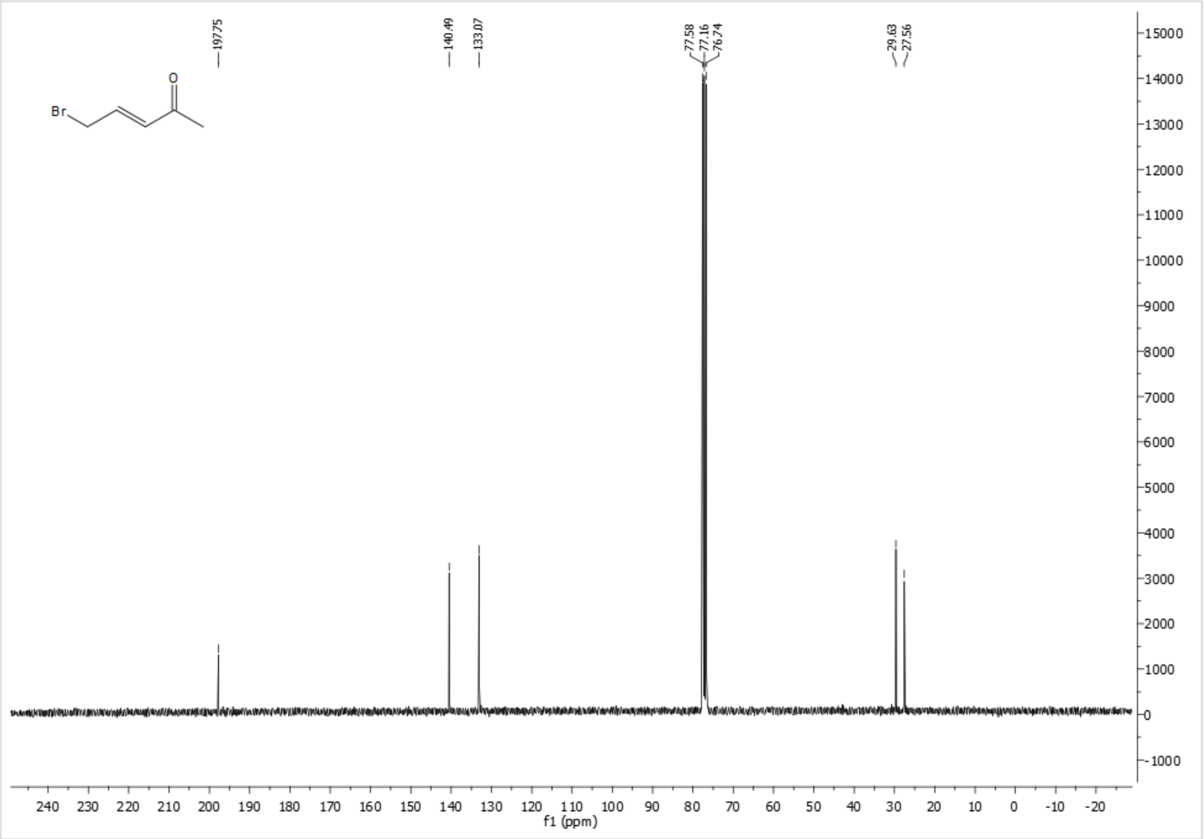

Figure S17: ^{13}C-NMR (75.53 MHz, $CDCl_3$) of compound **21**.

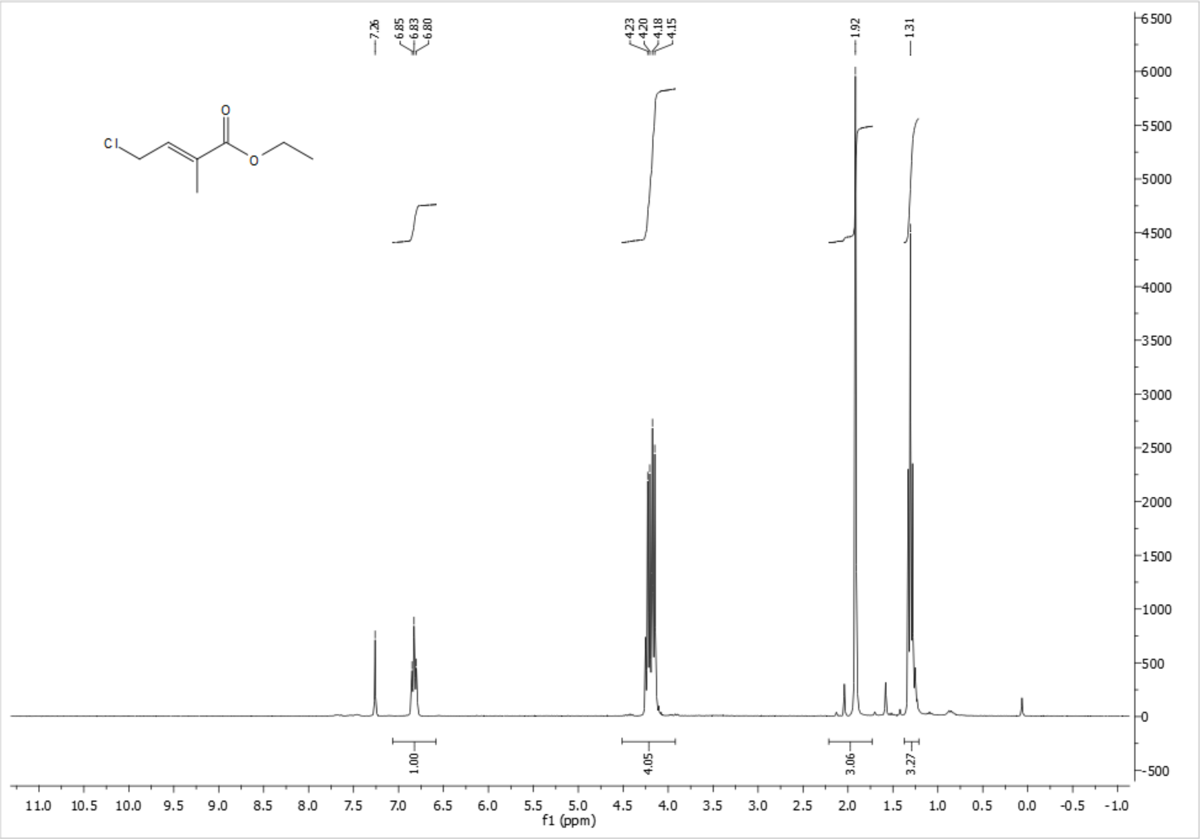

Figure S18: ^{1}H-NMR (300.36 MHz, $CDCl_3$) of compound **25**.

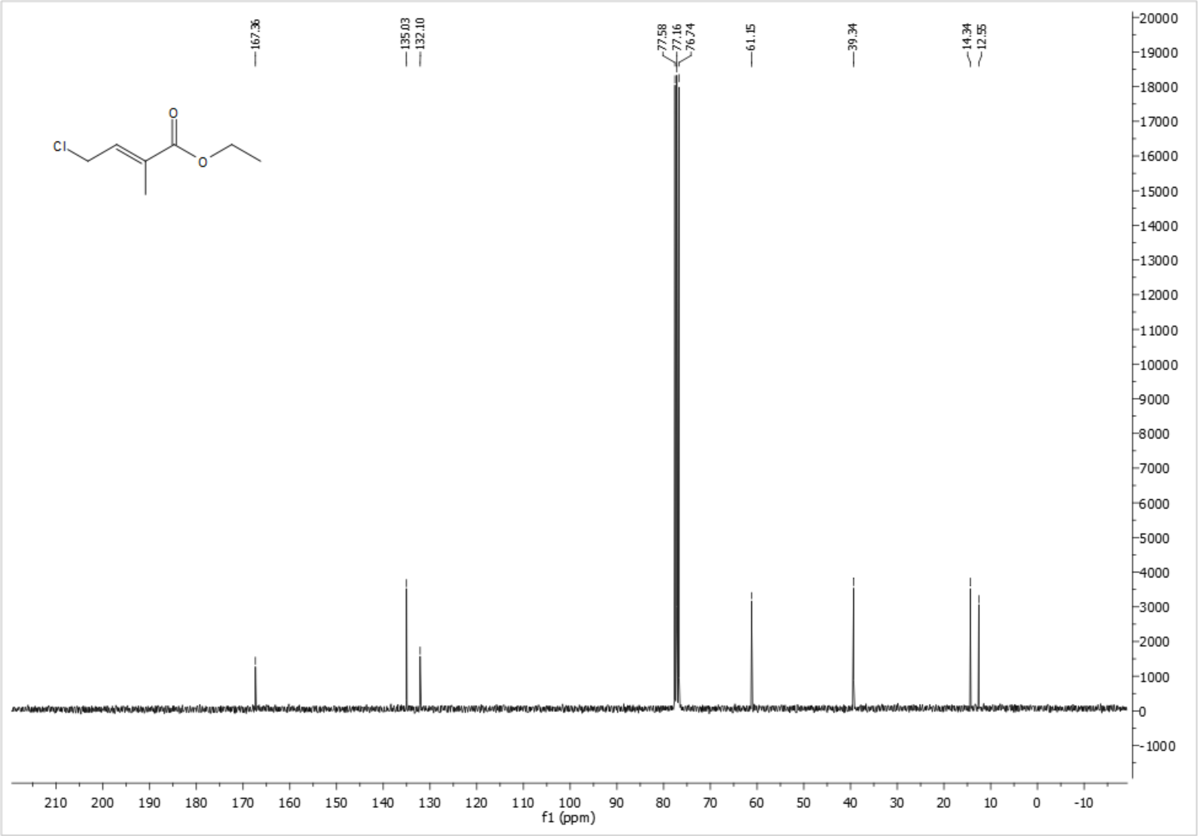

Figure S19: ^{13}C-NMR (75.53 MHz, $CDCl_3$) of compound **25**.

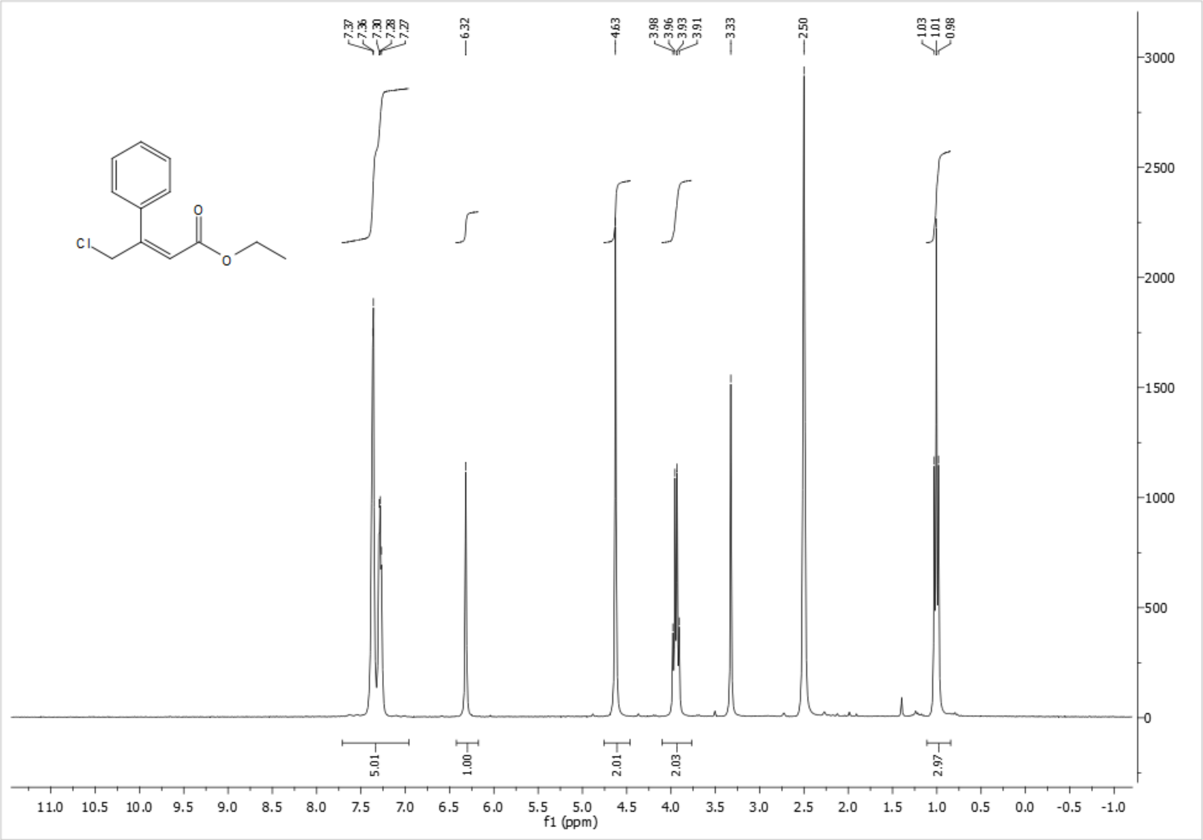

Figure S20: ^{1}H-NMR (300.36 MHz, DMSO-d_6) of compound **33**.

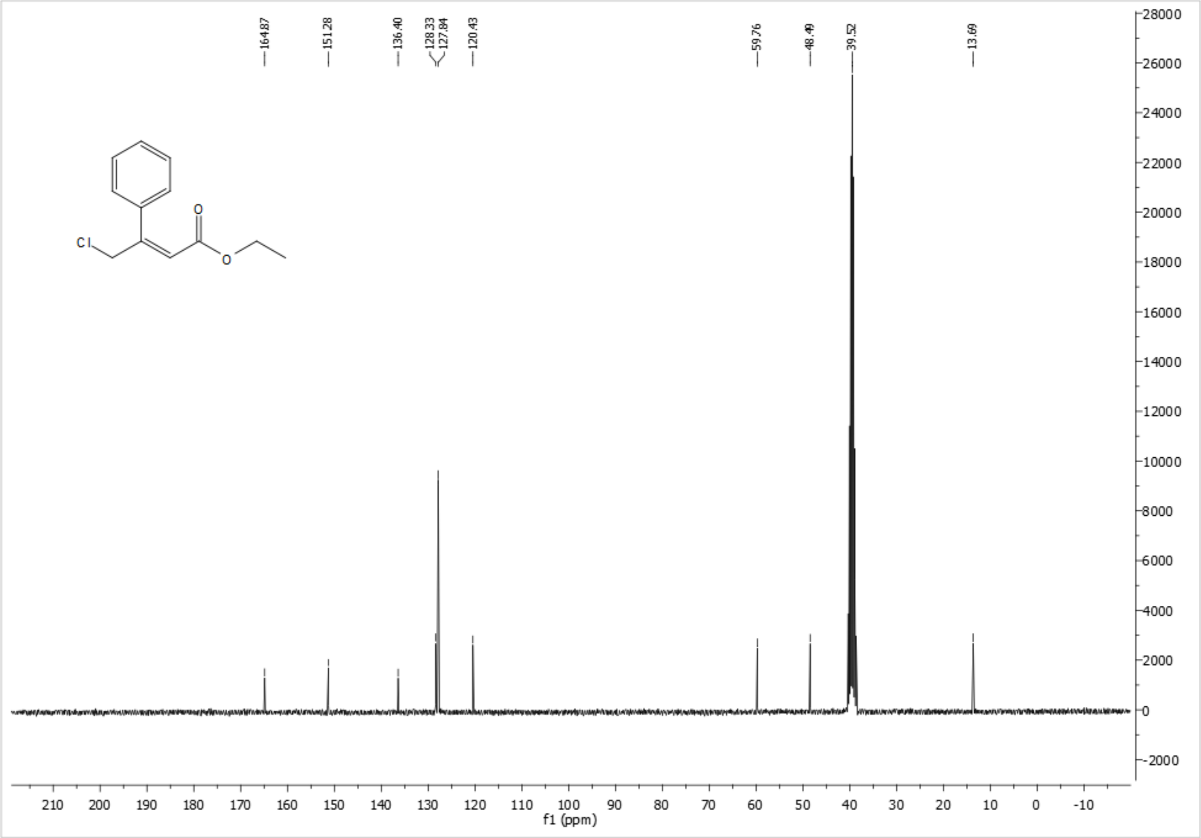

Figure S21: ^{13}C-NMR (75.53 MHz, DMSO-d_6) of compound **33**.

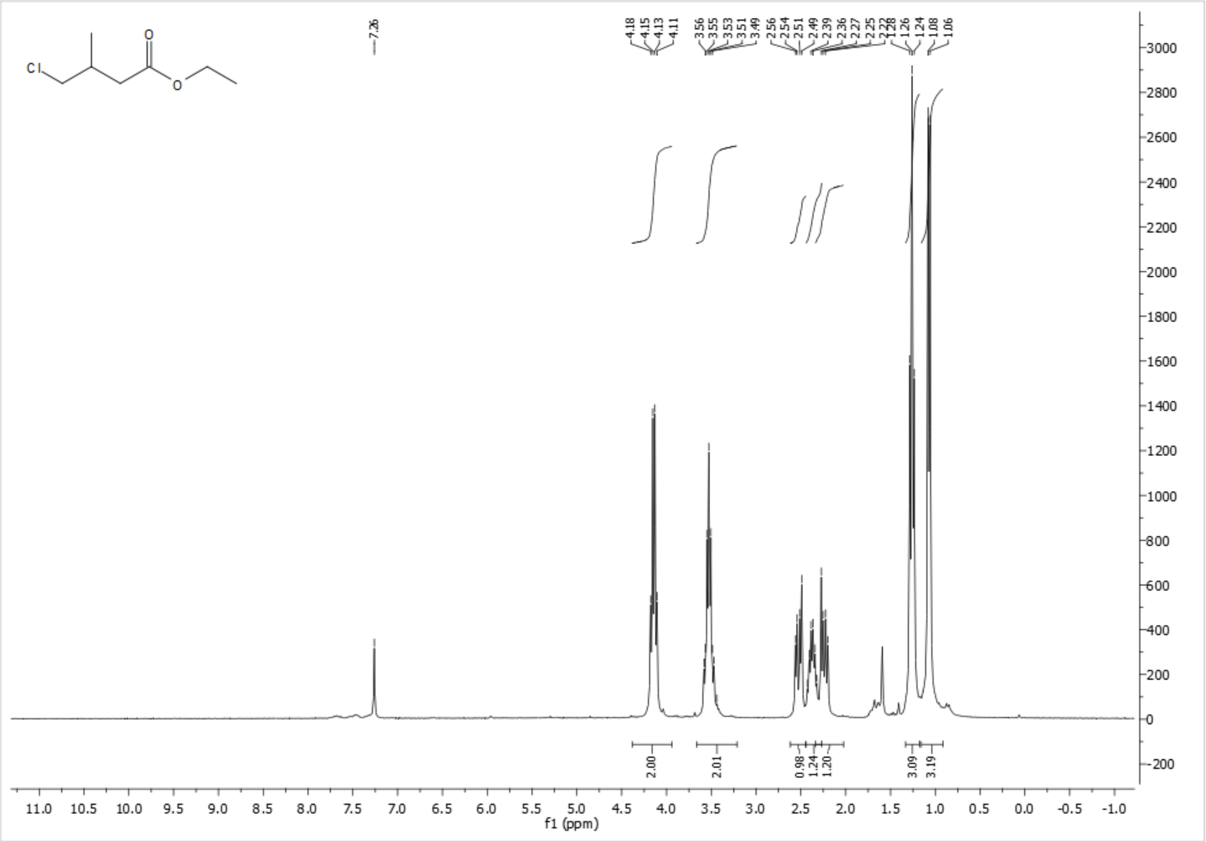

Figure S22: ^{1}H-NMR (300.36 MHz, $CDCl_3$) of compound **38**.

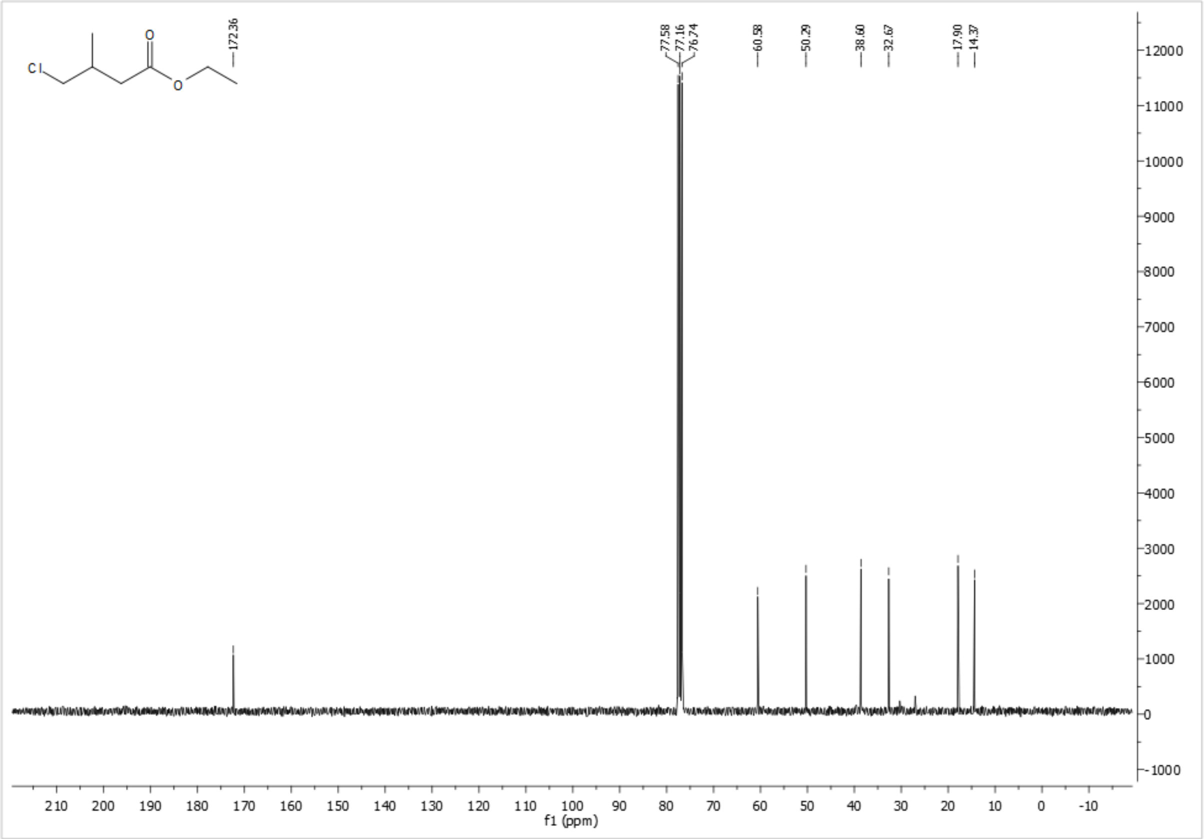

Figure S23:^{13}C-NMR (75.53 MHz, $CDCl_3$) of compound **38**.

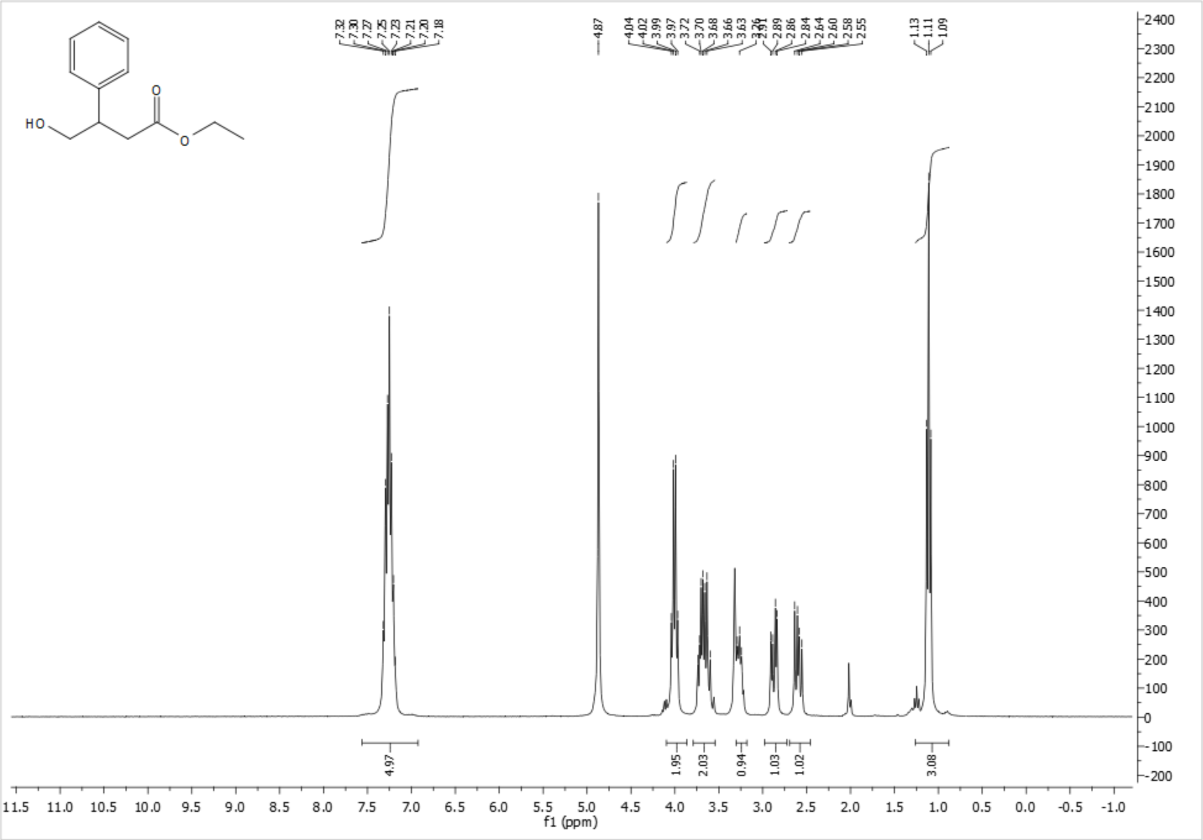

Figure S24: ^{1}H-NMR (300.36 MHz, MeOD) of compound **42**.

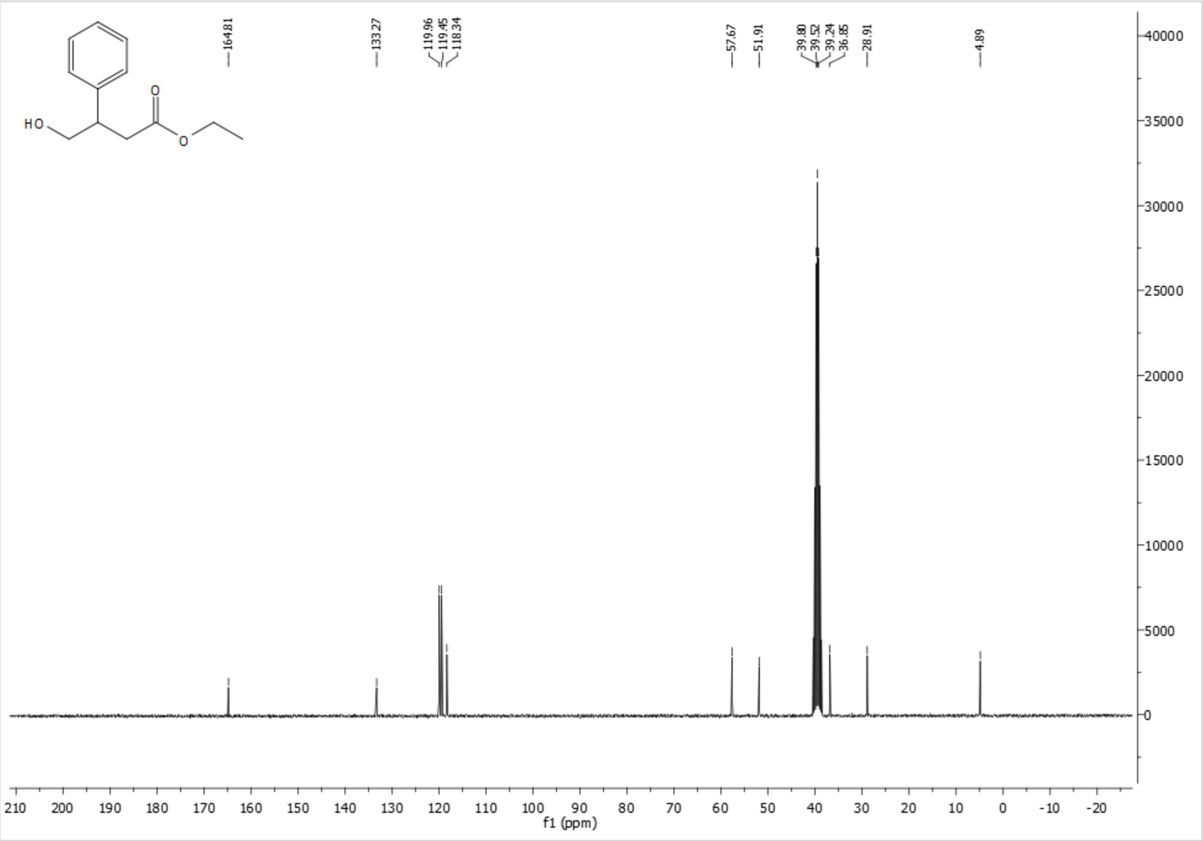

Figure S25: ^{13}C-NMR (75.53 MHz, MeOD) of compound **42**.

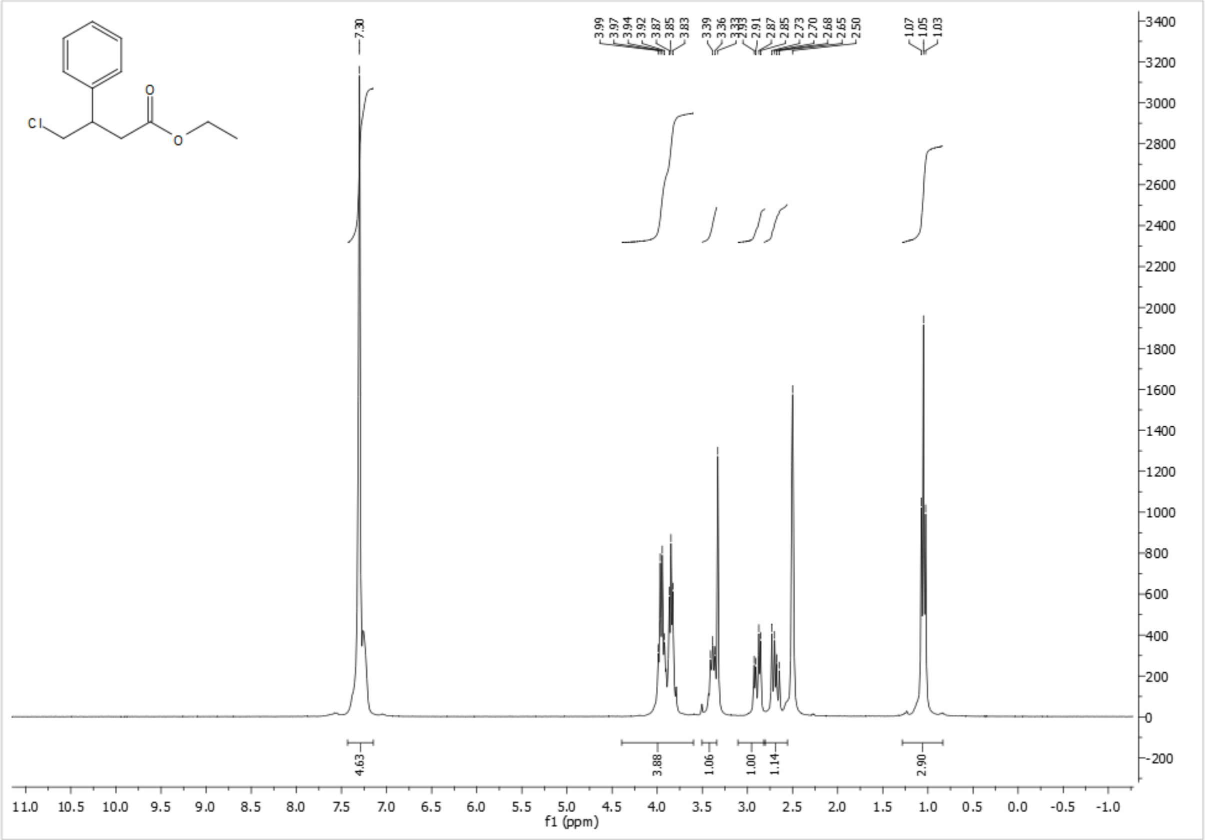

Figure S26: ^{1}H-NMR (300.36 MHz, DMSO-d$_6$) of compound **43**.

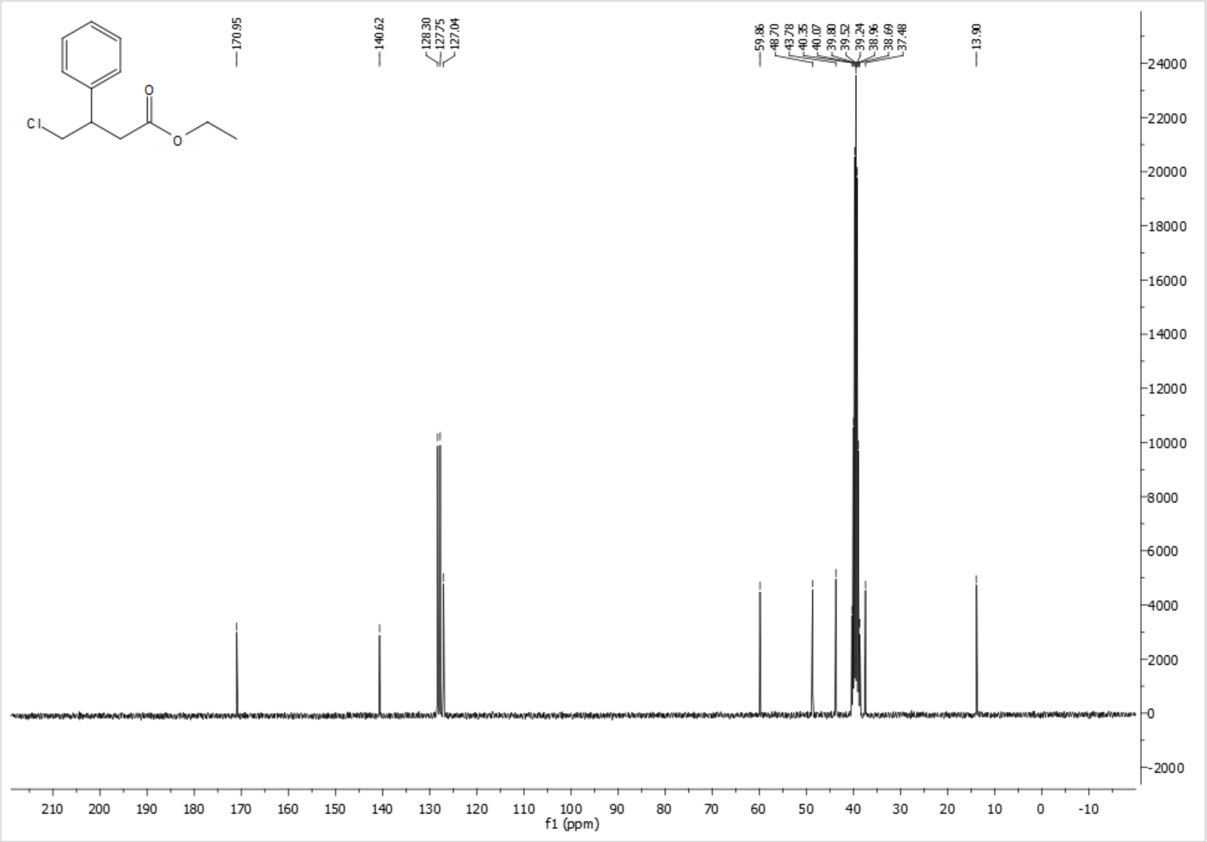

Figure S27: ^{13}C-NMR (75.53 MHz, DMSO-d$_6$) of compound **43**.

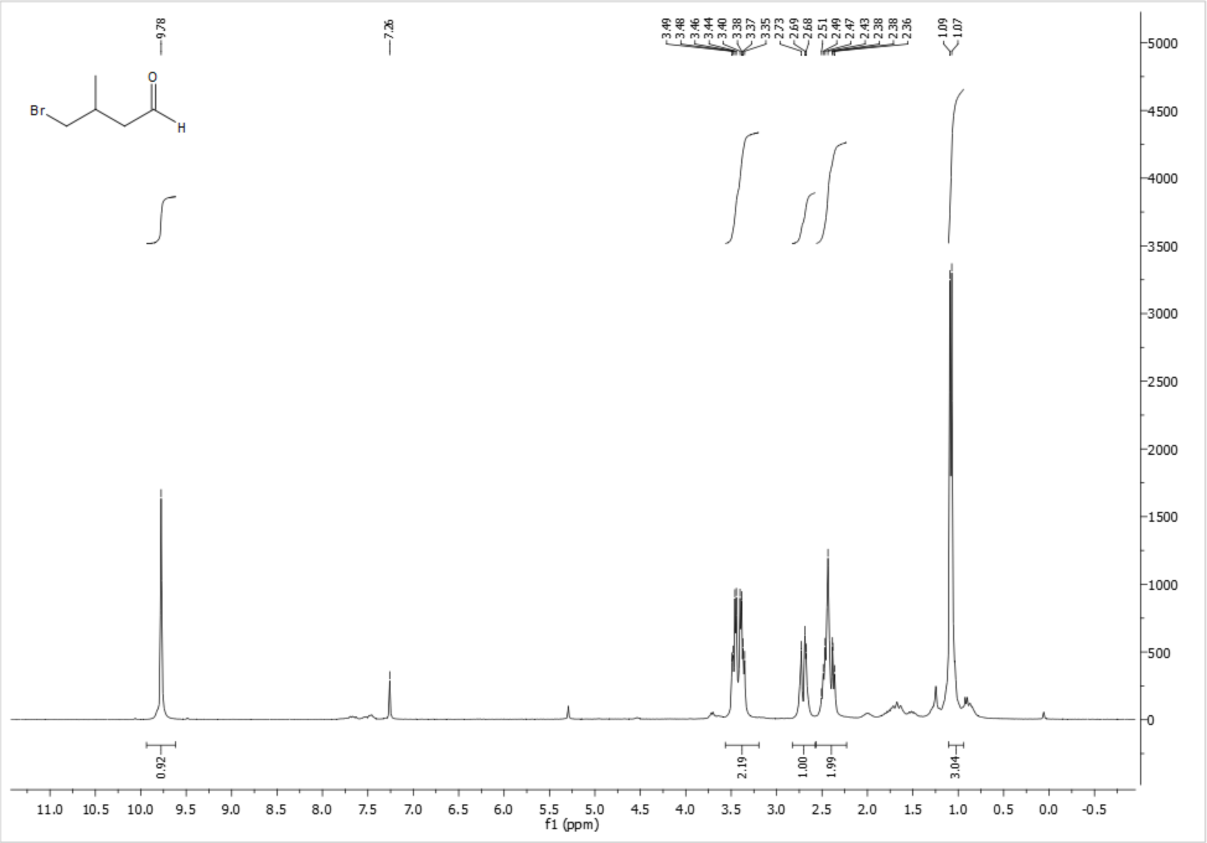

Figure S28: ^{1}H-NMR (300.36 MHz, $CDCl_3$) of compound **6-Br**.

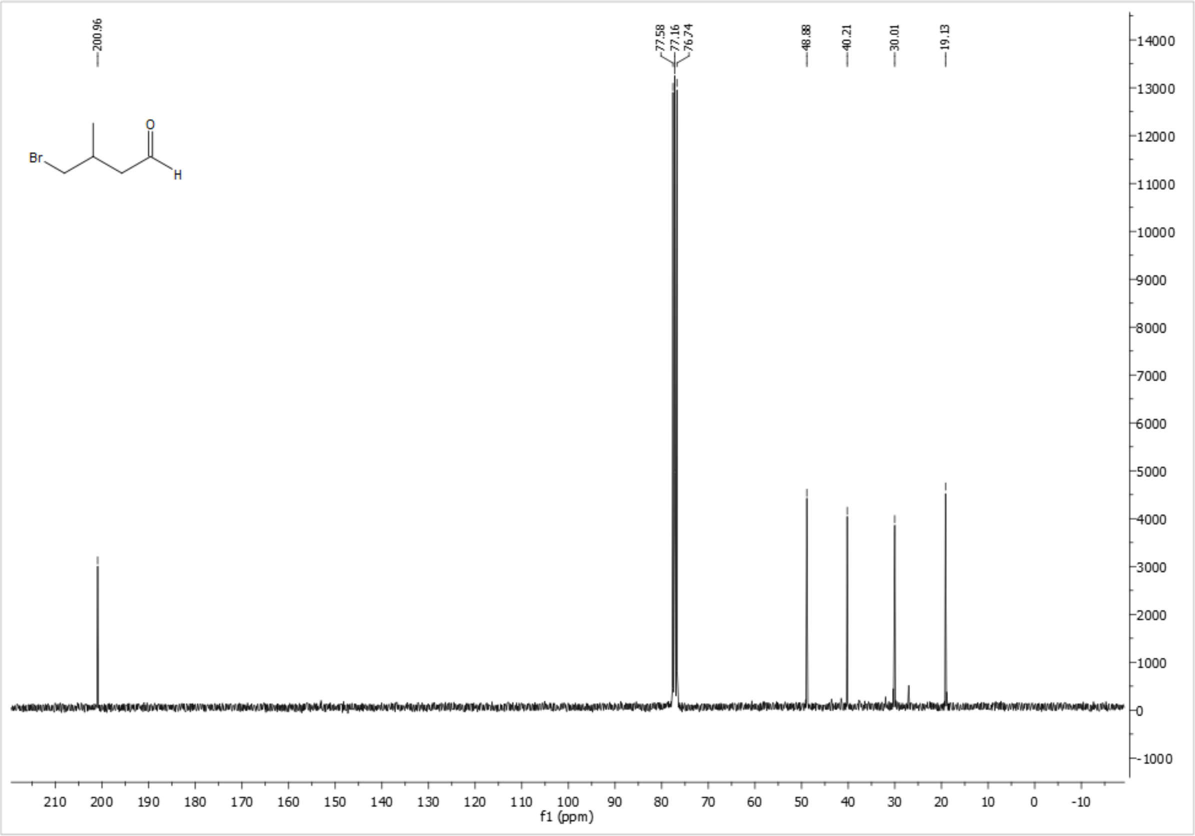

Figure S29: ^{13}C-NMR (75.53 MHz, $CDCl_3$) of compound **6-Br**.

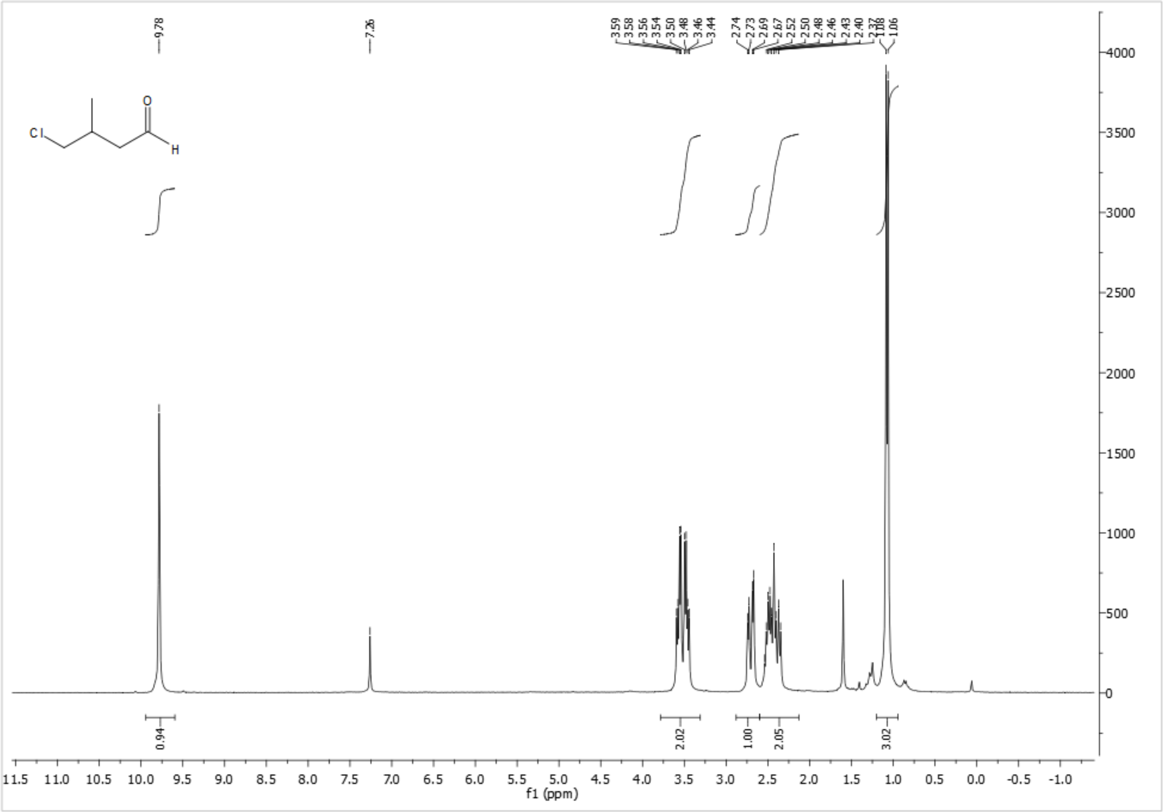

Figure S30: ^{1}H-NMR (300.36 MHz, $CDCl_3$) of compound **6-Cl**.

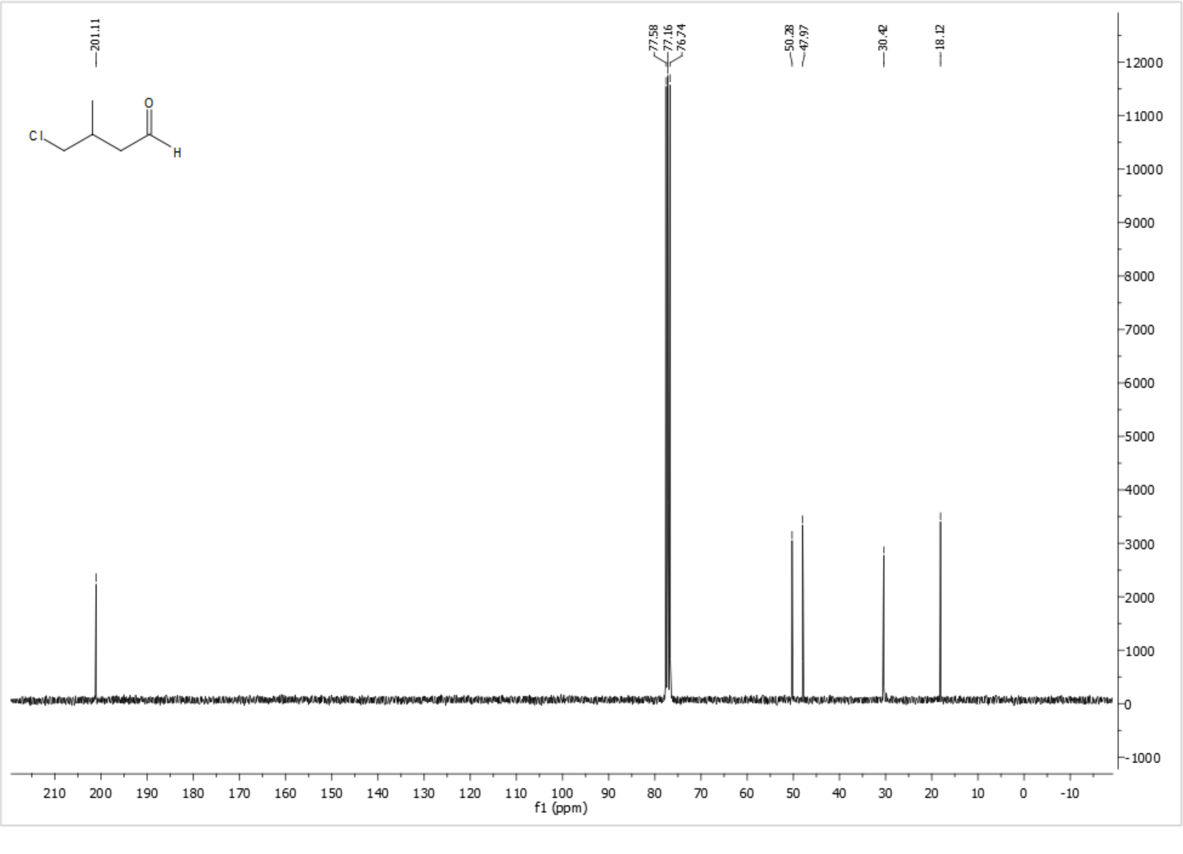

Figure S31: ^{13}C-NMR (75.53 MHz, $CDCl_3$) of compound **6-Cl**.

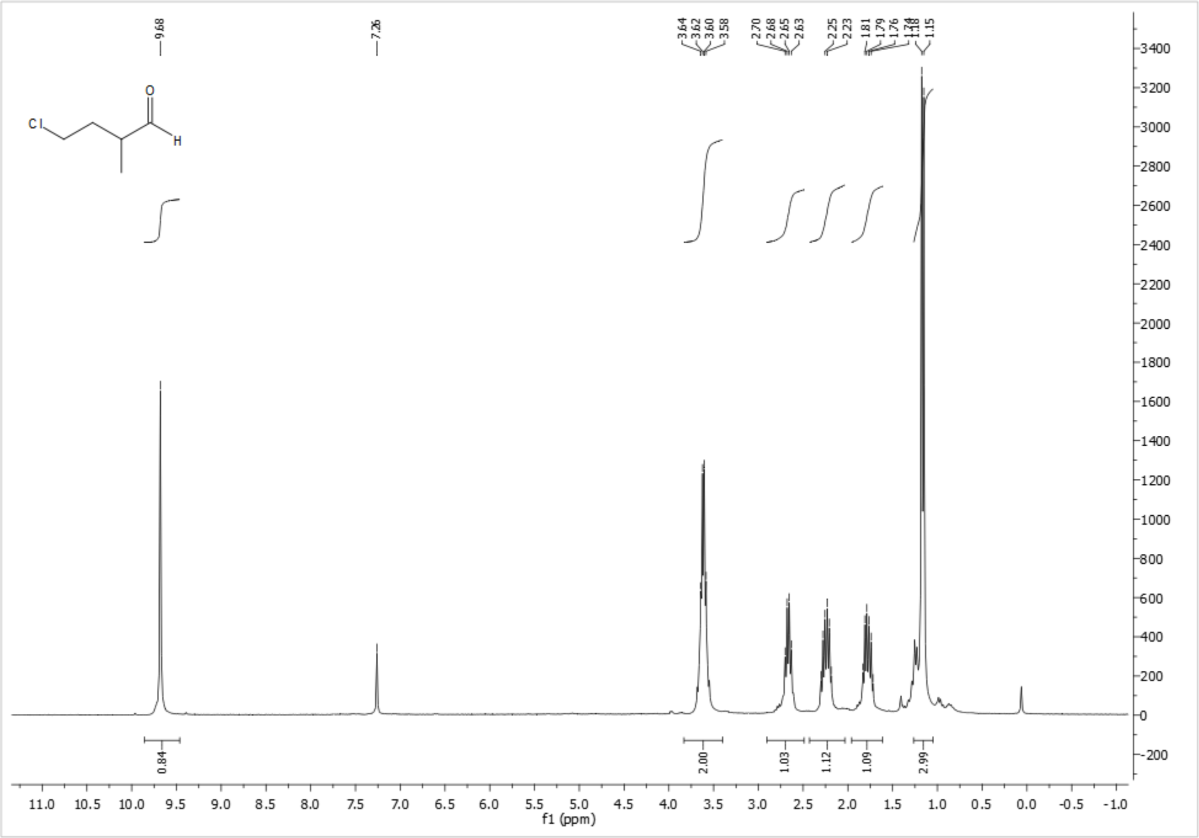

Figure S32: ^{1}H-NMR (300.36 MHz, $CDCl_3$) of compound **46**.

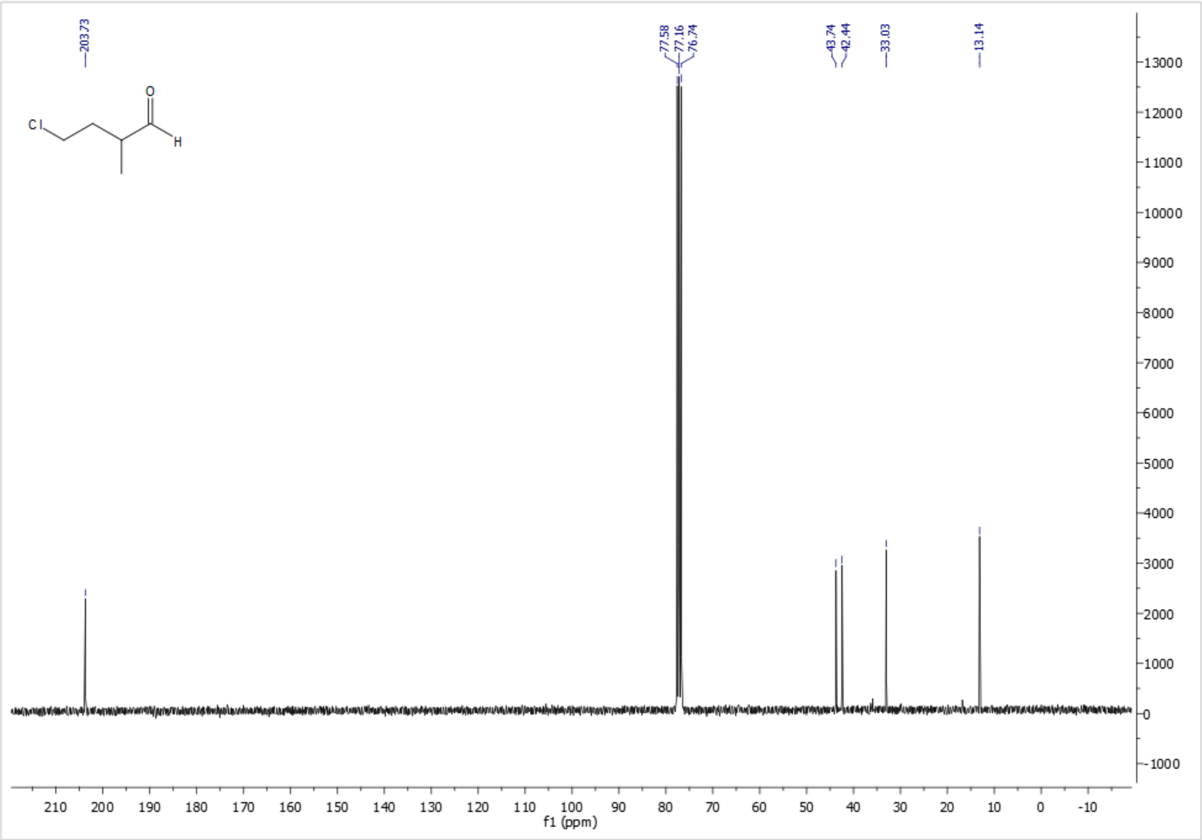

Figure S33: ^{13}C-NMR (75.53 MHz, $CDCl_3$) of compound **46**.

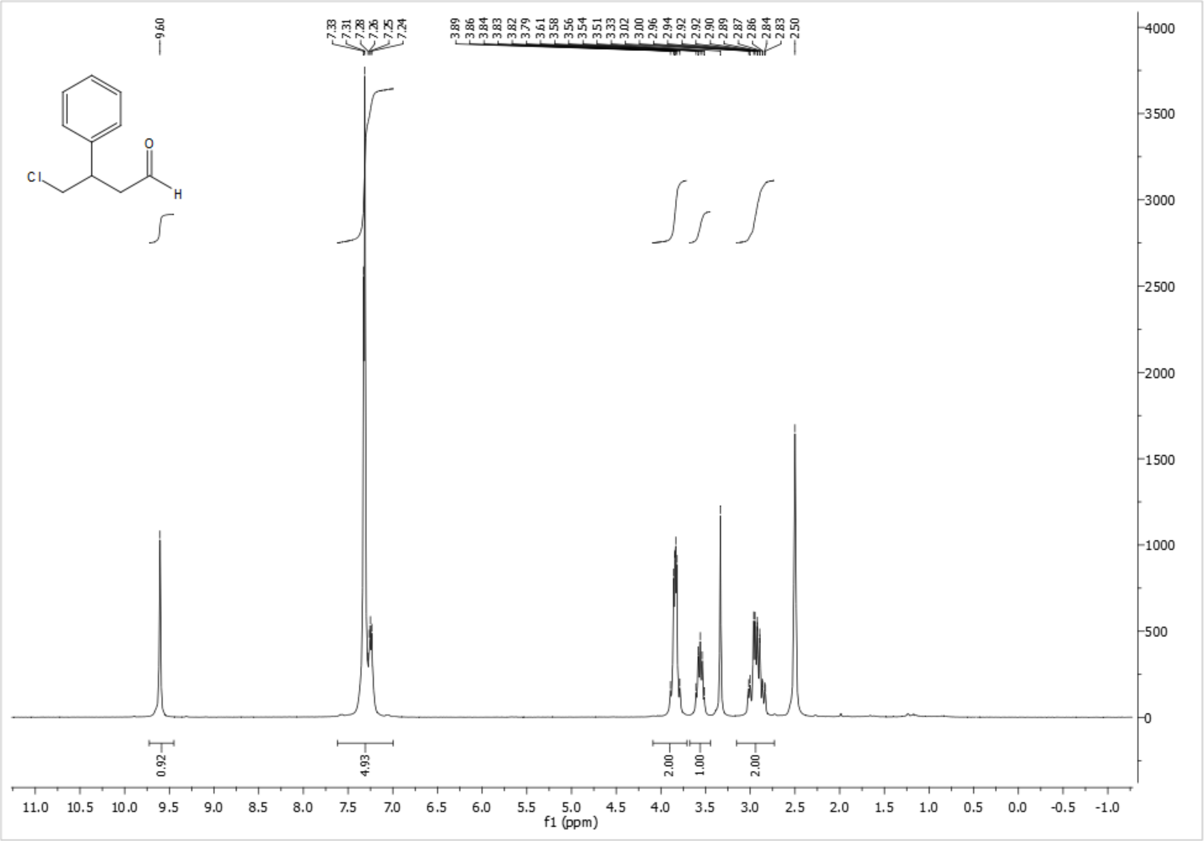

Figure S34: ^{1}H-NMR (300.36 MHz, DMSO-d_6) of compound **9**.

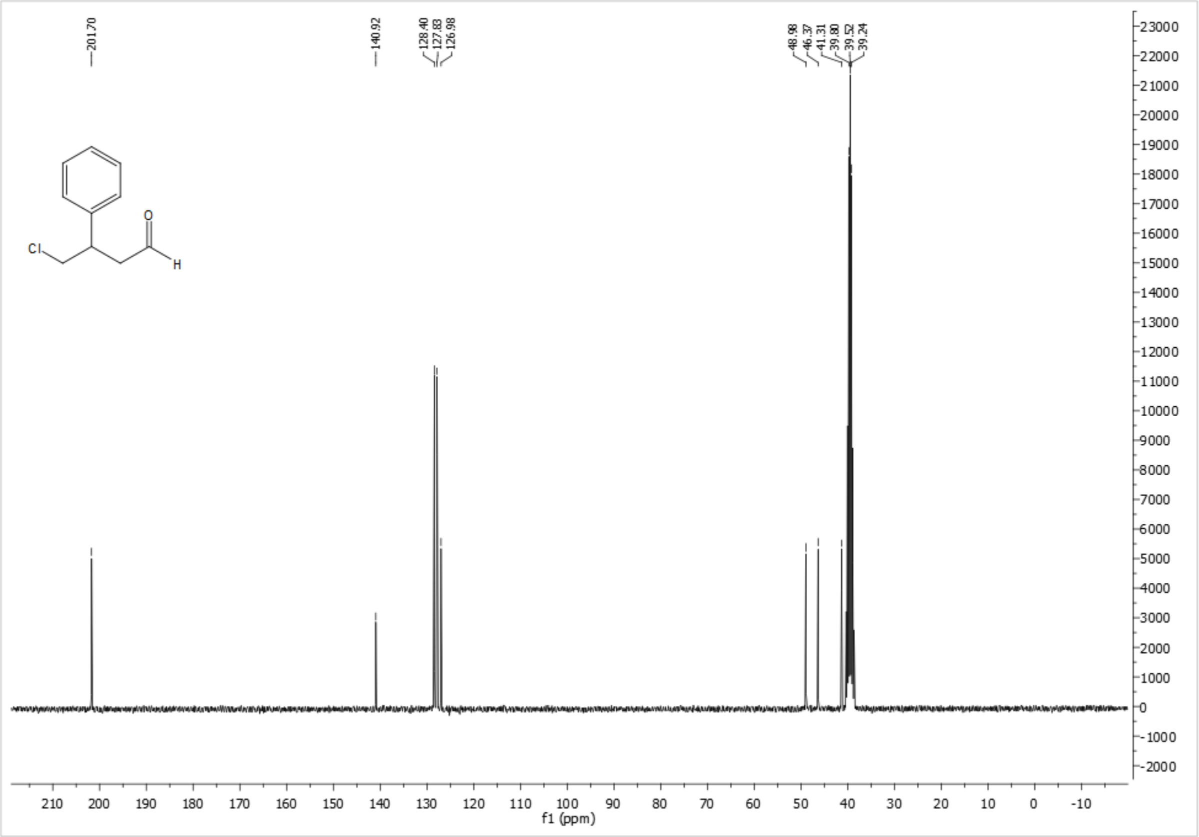

Figure S35: ^{13}C-NMR (75.53 MHz, DMSO-d_6) of compound **9**.

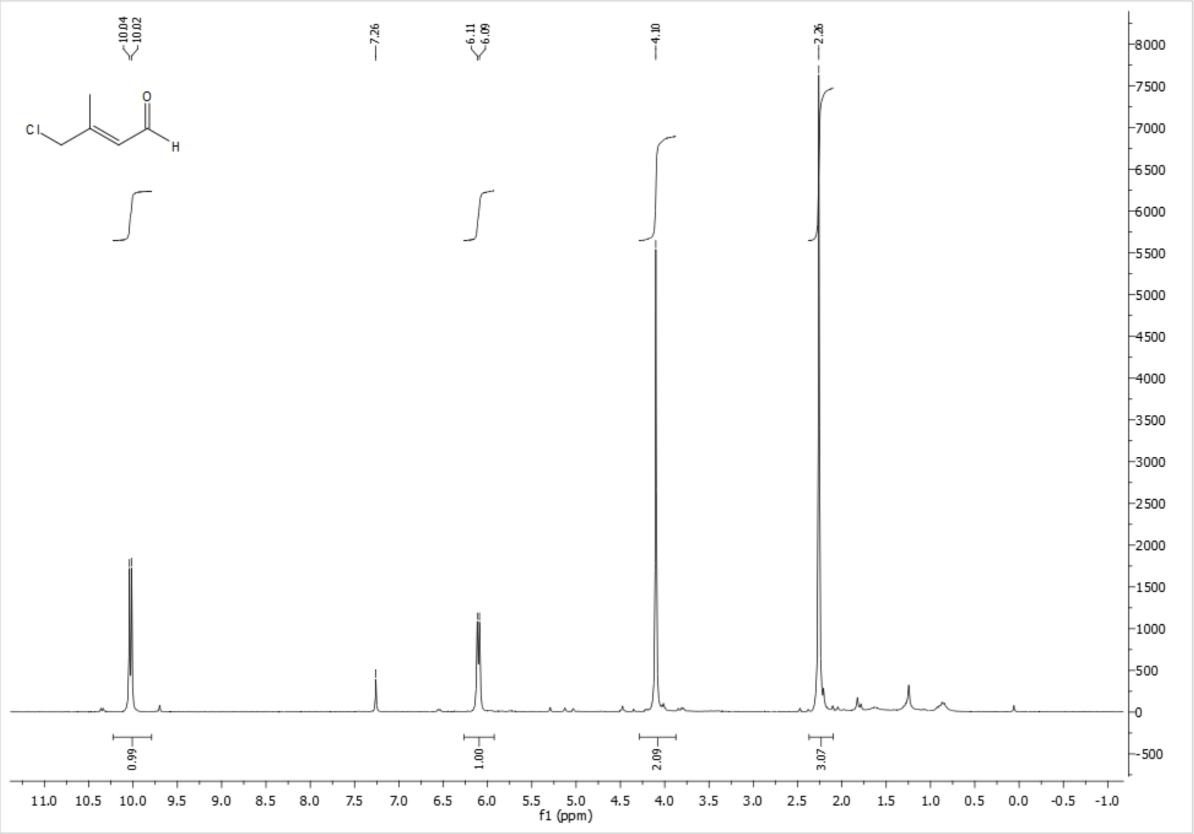

Figure S36: ^{1}H-NMR (300.36 MHz, $CDCl_3$) of compound **5-Cl**.

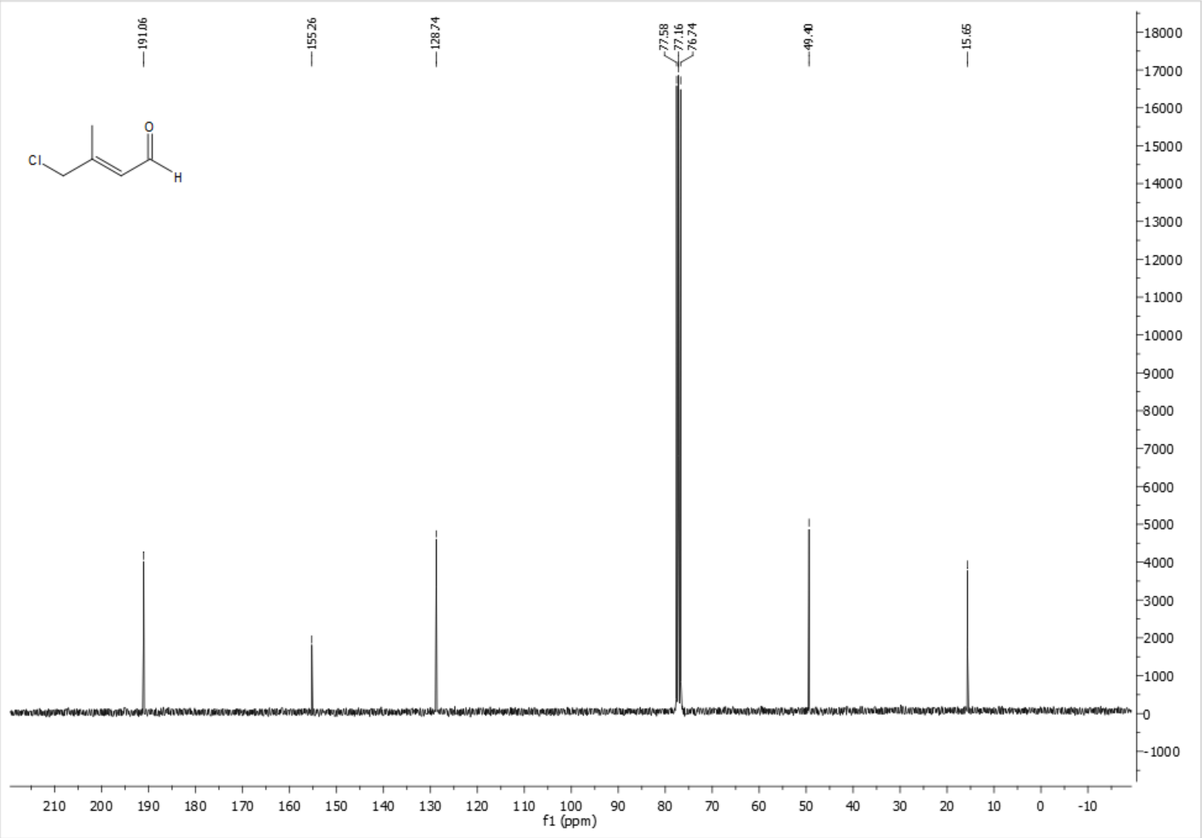

Figure S37: ^{13}C-NMR (75.53 MHz, $CDCl_3$) of compound **5-Cl**.

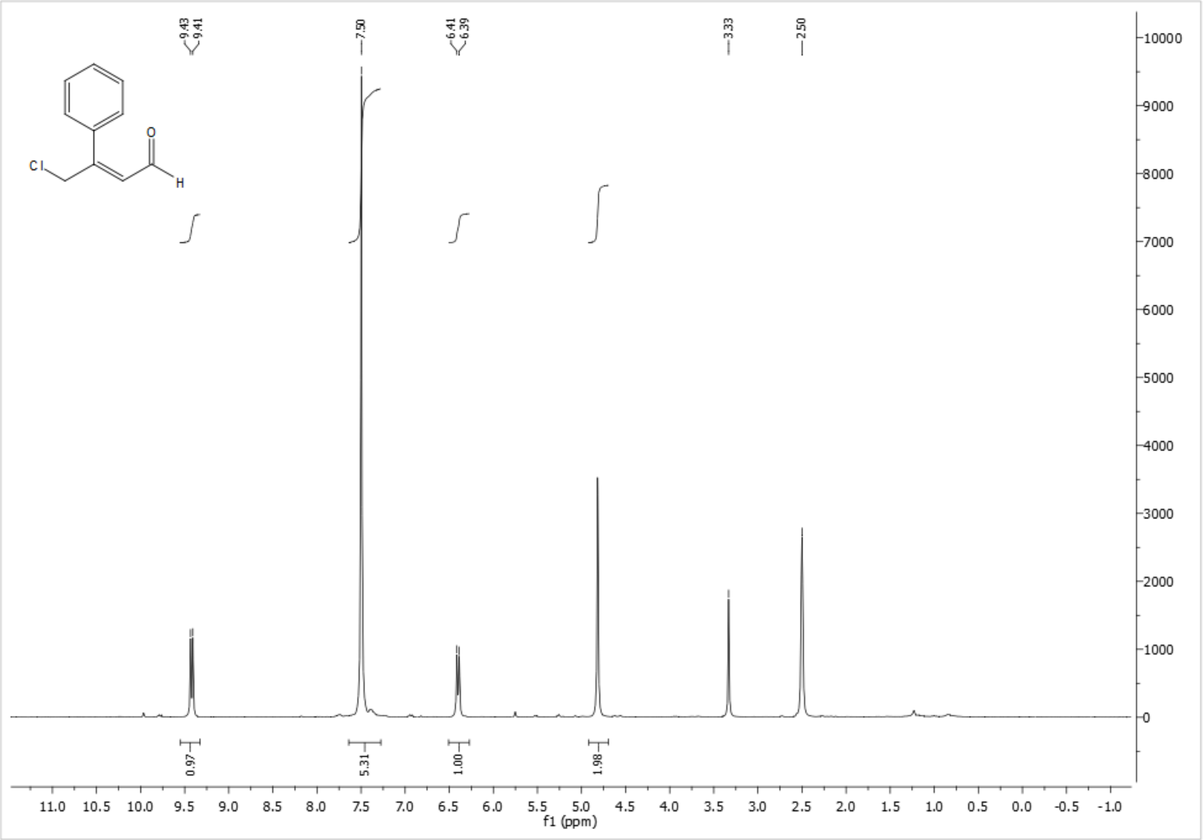

Figure S38: ^{1}H-NMR (300.36 MHz, DMSO-d_6) of compound **8**.

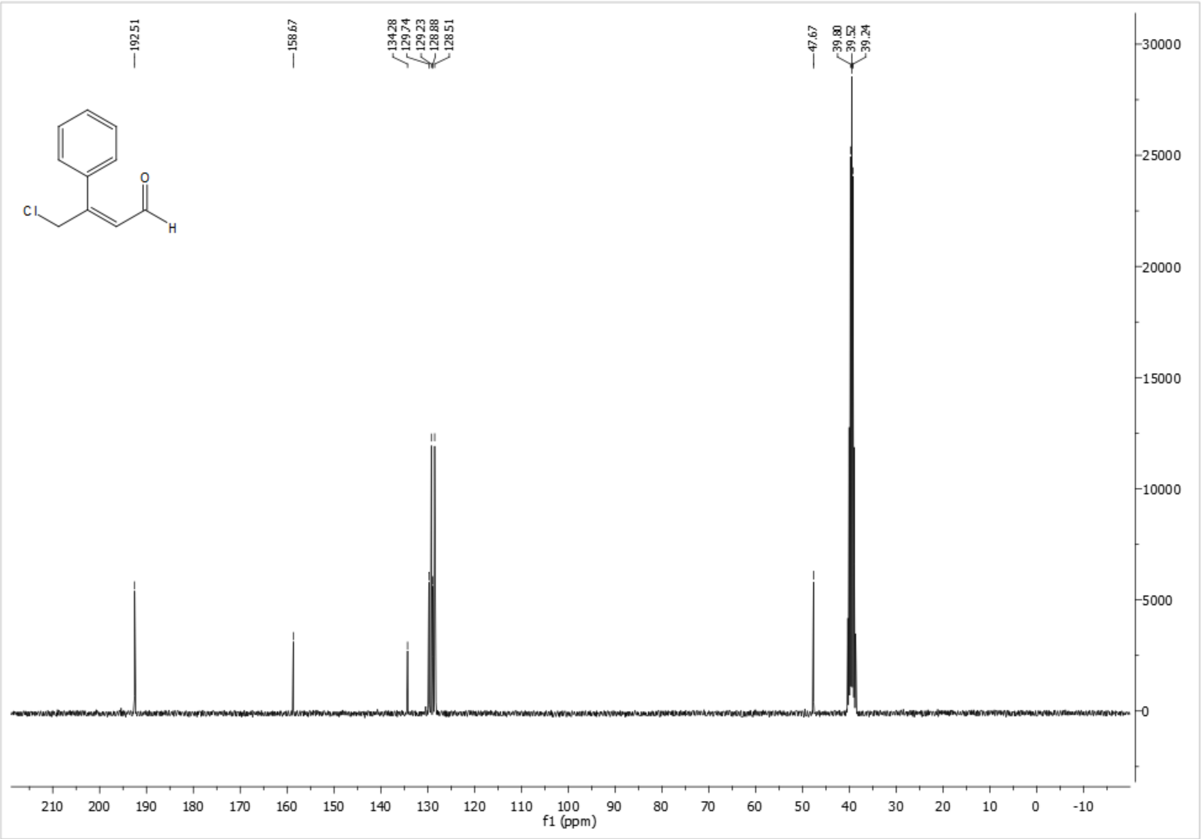

Figure S39: ^{13}C-NMR (75.53 MHz, DMSO-d_6) of compound **8**.

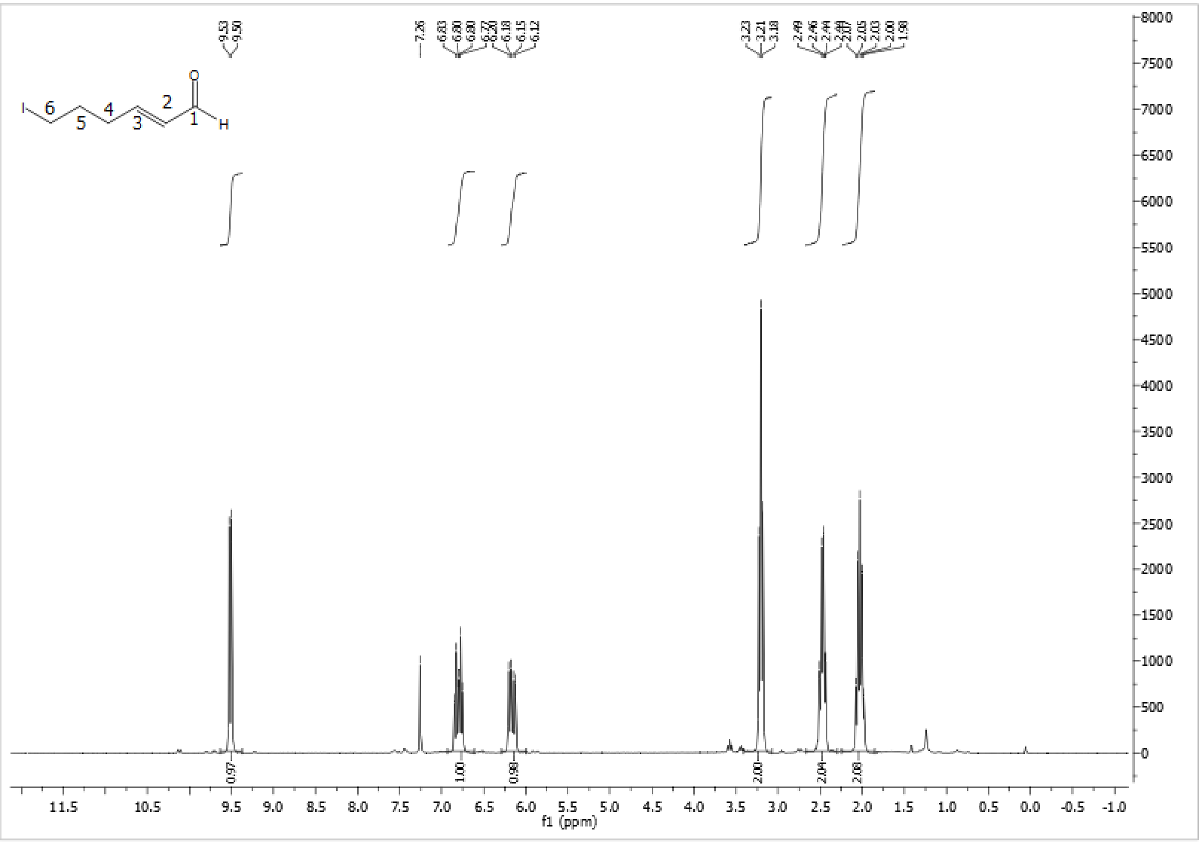

Figure S40: ^{1}H-NMR (300.36 MHz, $CDCl_3$) of compound **3a-I**.

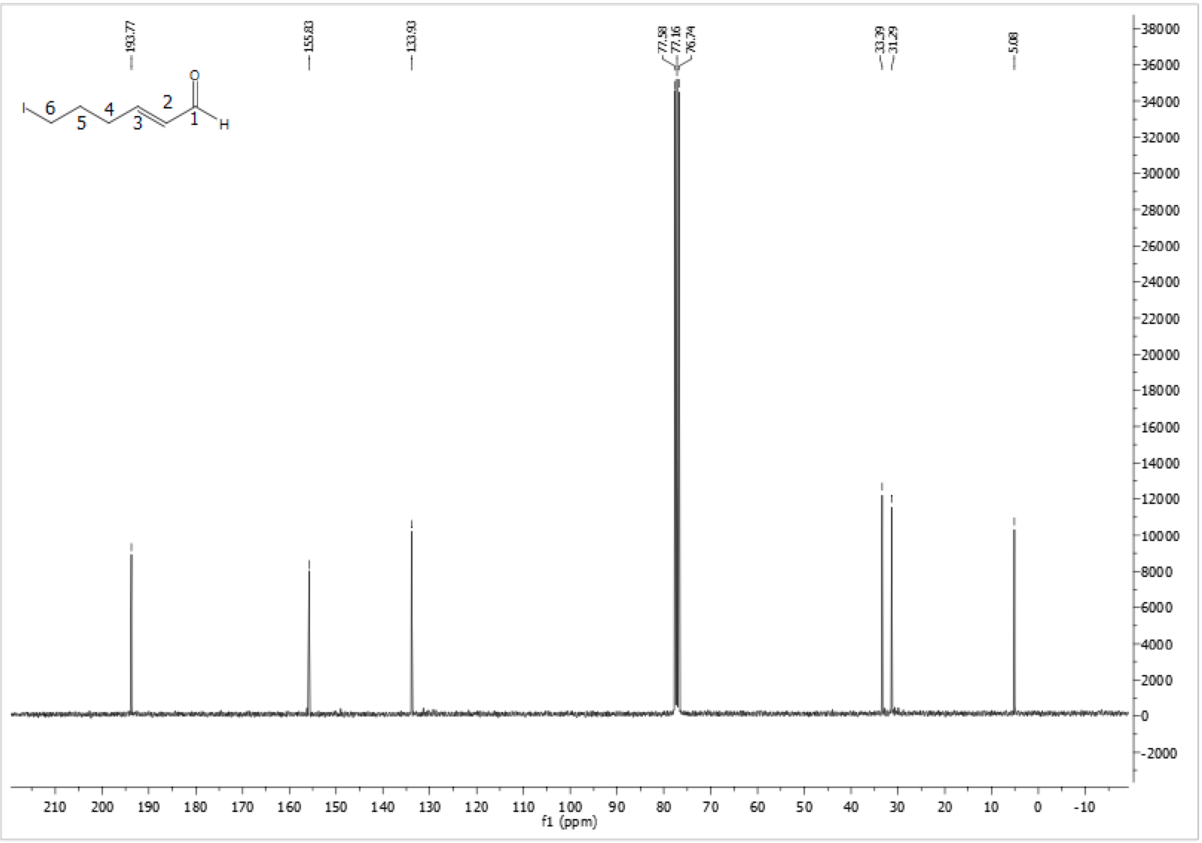

Figure S41: ^{13}C-NMR (75.53 MHz, $CDCl_3$) of compound **3a-I**.
